# LEARN: a multicentre, cross‐sectional evaluation of Urology teaching in UK medical schools

**DOI:** 10.1111/bju.15758

**Published:** 2022-05-26

**Authors:** Alexander Ng, Wai‐Shun Chan V, Aqua Asif, Alexander Light, Chon Meng Lam, Keerthanaa Jayaraajan, William A. Cambridge, Melissa Gillian Matthews, Meghana Kulkarni, Zhi Yang Ooi S, Arjun Nathan, Najma Ahmed, Shivali Gadhia, Naomi Morka, Zoe Hinchcliffe, Wentin Chen, Won Young Yoon, Kieran Das, Risata A. Kufuor, Kenal Patel, Ben Ayres, Jane Dacre, Chris Harding, Toby Page, Ian Pearce, Nikita R. Bhatt, Sinan Khadhouri, Veeru Kasivisvanathan, LEARN Study Group Collaborators, Ahaan Sanjay Gupta, Christopher Khoory, Owain Ellis, Maiar Elhariry, Lucia Harley, Viraj Shah, Hamza Umar, Reeshma Jameel, Jade Sangha, Miranda Ntorinkansah, Natalia Chilal, Anna Marshall, Balamrit Singh Sokhal, Vishal Chandanani, Caroline Jarman, Aishwarya Sharma, Hasti Tarzban, Maram Nabahin, Lewis Bonsell, Benjamin Langhorne, Prachi Agarwal, Rajwant Kaur, Alexander Hunt, Cecilia Cirelli, Natasha Alford, Nesta Baxter, Anneesa Malik, Abigail Harrison, Monty Matson, Ronak Shah, Cliona Meenan, Daanish Ghaffar, Katherine Wise, Lauren Gurr, Tahmeed Ahmed, Alice Jones, Ankur Singh, Chloe Stevens, Lien Salcedo, Michael Cooke, Sunwoo Lee, Amanda Godoi, Bethany Rose, Oluwajenrola Arawole, Sanjana Ilangovan, Alexander West, Alna Dony, Anoop Sumal, Ariadni Papadopoulou, Matan Bone, Omar Haque, Pooja Patel, Rawan Al Dehailan, Robert Grogan, Brishti Debnath, Carina Synn Cuen Pan, Isabelle Shaw, Katie Tsang, Marcus Graham, Milad Parsi, Nidhi Agarwal, Sarah Pengelly, Shabnam Tariq, Sumbal Bhatti, Greta Safoncik, Holly Thompson, Italia Rosa‐Leech, Maia Osborne‐Grinter, Matthew Hennessy, Ramisha Basharat, Rebecca Paterson, Sherie George, Shubham Gupta, Victoria Porter, Ashley Soloman, Attika Chaudhary, Briony Seden, Eimad Basit, Grace Kettyle, Jesvin Sunny, Madhumita Kolluri, Precious Jolugbo, Syme Bhopal, Tzvi Reich, Alexander Davies, Eleanor Kissane, Elena Missir, Gareth Hutchinson, Jonathan Chua, Nicole Wang, Olivia Pestrin, Ryan Faulder, Thomas McLelland, Vikash Patel, Aimee Wilkinson, Aliraza Syed, Amrit Mann, Christina Huon, Elina Stokolova, Elisa Lau, Ella Hobbs, George Garratt, George Higginbotham, Harriet Flashman, Hassan Ismahel, Ivana Homerova, Marcus Boyd, Rafi Abdullah, Rebecca Lim, Rebecca Vitarana, Sasha Quarrington, Vaishali Kiridaran, Ziad Zeidan, Alexis Adam, Ananya Nair, Bernice Johal, Brooke Gerrie, Christopher Gunn, Cora Lowe, Dhikshitha Nagaraj, Eleanor Deane, Jamie McGinn, Jennifer Luu, Karan Sagoo, Katie Sharman, Laura Cunningham, Megan Scotcher, Meshva Amin, Natasha Aghtarafi, Ruth Goh, Shi Pei Loo, Tatiana Hamakarim, Timothy Ho, Xinyu Ye, Zain Islam, Zoe Zagorac, Abdal Zafar, Alisha Kanani, Armin Nazari, Daniel Sescu, Inas Alsuhaibani, Isabel Munden, Jasmine Pattarukuzhyil Jose, Mariella Fortune‐Ely, Michaela Rogers, Pratyush Pradeep, Stephanos Ghobrial, Thomas Hall, Yasmin Motarjemi, Aaron Campbell, Allen Royal, Anna Kruczynska, Aqeeb Mahmood, Ateeq Jamil, Boris Wagner, Caitlin Murphy, Catriona Walker, Dilen Parmar, Isabelle Mayne, Jason Armstrong, Laura Inglis, Lily Waltham, Natalie Ko, Rhiannon Kirk, Roshni Johnson, Ryan Turner, Serena Patel, Sita Asi, Veena Sudarshan

**Affiliations:** ^1^ University College London (UCL) Medical School UCL London UK; ^2^ British Urology Researchers in Surgical Training (BURST) London UK; ^3^ School of Medicine, Faculty of Medicine and Health University of Leeds Leeds UK; ^4^ Leicester Medical School University of Leicester Leicester UK; ^5^ Department of Surgery and Cancer Imperial College London London UK; ^6^ Bronglais General Hospital Aberystwyth UK; ^7^ Faculty of Medicine Imperial College London London UK; ^8^ University of Edinburgh Edinburgh UK; ^9^ Hull York Medical School York UK; ^10^ Department of Urology Urology Centre, Guy’s and St Thomas’ NHS Foundation Trust London UK; ^11^ Cancer Imaging Department, School of Biomedical Engineering and Imaging Sciences King’s College London London UK; ^12^ Cardiff University School of Medicine University Hospital Wales Cardiff UK; ^13^ Division of Surgery and Interventional Science UCL London UK; ^14^ GKT School of Medicine, Faculty of Life Sciences and Medicine King’s College London London UK; ^15^ Sheffield Medical School University of Sheffield Sheffield UK; ^16^ School of Medicine Cardiff University Cardiff UK; ^17^ Birmingham Medical School, College of Medical and Dental Sciences University of Birmingham Birmingham UK; ^18^ School of Medical Sciences, Faculty of Biology, Medicine and Health University of Manchester Manchester UK; ^19^ Department of Bioengineering Imperial College London London UK; ^20^ School of Medicine University of Nottingham Nottingham UK; ^21^ School of Medicine, Faculty of Medicine Imperial College London UK; ^22^ Department of Urology St George’s University Hospitals NHS Trust London UK; ^23^ UCL Medical School London UK; ^24^ Department of Urology Freeman Hospital Newcastle upon Tyne UK; ^25^ Translational and Clinical Research Institute Newcastle University Newcastle upon Tyne UK; ^26^ Department of Urology Freeman Hospital, Newcastle upon Tyne Hospitals Trust Newcastle upon Tyne UK; ^27^ Manchester University NHS Foundation Trust Manchester UK; ^28^ Department of Urology Norfolk and Norwich University Hospital Norwich UK; ^29^ Health Science Research Unit, The School of Medicine, Medical Sciences and Nutrition, Aberdeen Royal Infirmary University of Aberdeen Aberdeen UK; ^30^ Division of Surgery and Interventional Science UCL London UK

**Keywords:** urology, undergraduate, education, medical students, teaching, clinical skills, urology curriculum, #Urology

## Abstract

**Objective:**

To evaluate the status of UK undergraduate urology teaching against the British Association of Urological Surgeons (BAUS) Undergraduate Syllabus for Urology. Secondary objectives included evaluating the type and quantity of teaching provided, the reported performance rate of General Medical Council (GMC)‐mandated urological procedures, and the proportion of undergraduates considering urology as a career.

**Subjects and Methods:**

The uroLogical tEAching in bRitish medical schools Nationally (LEARN) study was a national multicentre cross‐sectional evaluation. Year 2 to Year 5 medical students and Foundation Year (FY) 1 doctors were invited to complete a survey between 3 October and 20 December 2020, retrospectively assessing the urology teaching received to date. Results are reported according to the Checklist for Reporting Results of Internet E‐Surveys (CHERRIES).

**Results:**

In all, 7063/8346 (84.6%) responses from all 39 UK medical schools were included; 1127/7063 (16.0%) were from FY1 doctors who reported that the most frequently taught topics in undergraduate training were on urinary tract infection (96.5%), acute kidney injury (95.9%) and haematuria (94.4%). The most infrequently taught topics were male urinary incontinence (59.4%), male infertility (52.4%) and erectile dysfunction (43.8%). Male and female catheterisation on patients as undergraduates was performed by 92.1% and 73.0% of FY1 doctors respectively, and 16.9% had considered a career in urology. Theory‐based teaching was mainly prevalent in the early years of medical school, with clinical skills teaching, and clinical placements in the later years of medical school. In all, 20.1% of FY1 doctors reported no undergraduate clinical attachment in urology.

**Conclusion:**

The LEARN Study is the largest ever evaluation of undergraduate urology teaching. In the UK, teaching seemed satisfactory as evaluated against the BAUS undergraduate syllabus. However, many students report having no clinical attachments in Urology and some newly qualified doctors report never having inserted a catheter, which is a GMC mandated requirement. We recommend a greater emphasis on undergraduate clinical exposure to urology and stricter adherence to GMC mandated procedures.

**Abbreviations**
BURST: British Urology Researchers in Surgical Training
COVID‐19: Coronavirus disease 2019
FY: Foundation Year
GMC: General Medical Council
IQR: Interquartile range
LEARN: uroLogical tEAching in bRitish medical schools Nationally
MLA: Medical Licensing Assessment
OSCE: objective structured clinical examination
SARS‐CoV‐2: Severe acute respiratory syndrome coronavirus‐2

## Introduction

Urology is a prominent surgical specialty, comprising 9.7% of all surgical consultants across the NHS in England [1]. Urological conditions account for 10%–15% of GP appointments and 21.9% of acute surgical referrals, and one in five NHS hospital inpatients will have a urinary catheter inserted during their admission [2, 3, 4]. Urology is a common rotation amongst newly qualified doctors during Foundation Years (FYs), but there is concern that current teaching and exposure during medical school does not fully prepare them to manage basic urological conditions [5]. This has been an issue internationally, dating as far back as 1966 [6]. In Europe, urology teaching is mandatory in 76% of universities, whilst in the USA, there has been a decrease in the proportion of medical schools mandating a urology rotation from 99% in 1956, to 17% in 2010 [7, 8]. A 2001 UK study reported two medical schools did not require any urology exposure for students to graduate [9]; and in the USA 65% of medical schools do not have mandatory urological exposure [10].

The paradigm shift of the undergraduate curricula towards primary care and student‐selected components (SSCs) [2] has resulted in graduates receiving on average 1 week of clinical urological experience during medical school [11], despite previous reports suggesting that students should receive 2–3 weeks to reflect the prevalence of urological conditions [9]. It is difficult for students to attain necessary skills from clinical exposure and to consider urology as their future specialty given the absence or short duration of urological placements.

Whilst the standards and outcomes for medical education in the UK are regulated by the General Medical Council (GMC) [12], variability in the exposure to each specialty still exists [2]. In 2012, the BAUS released ‘An Undergraduate Syllabus for Urology’ [2]. Despite the publication of this national syllabus ten years ago, its national adherence remains unknown.

The uroLogical tEAching in bRitish medical schools Nationally (LEARN) Study was developed in recognition of this [5, 13]. Our aim was to assess undergraduate urology teaching across UK medical schools in line with the BAUS syllabus to be able to inform governing bodies, educational institutes and associations that provide guidance and regulations to medical schools.

## Subjects and Methods

### Study Design

We conducted a multicentre retrospective cross‐sectional study, co‐ordinated by the British Urology Researchers in Surgical Training (BURST) Research Collaborative [14, 15].

A web‐based survey was developed and included binomial, variable‐scale, visual analogue scale, and free‐text response options (Appendix S2). Study data were collected and managed using REDCap (Research Electronic Data Capture) hosted at University College London [16]. The survey was piloted by the LEARN Steering Committee prior to dissemination. Responses were collected over an 11‐week period (3 October to 20 December 2020). This time period was specifically chosen as it is the start of the academic year. The results were reported according to the Checklist for Reporting Results of Internet E‐Surveys (CHERRIES) (Appendix S3) [17].

The survey retrospectively assessed the urology teaching those individuals had received to date. For example, Year 2 students’ data reflected the teaching they received in Year 1 and Year 3 reflected teaching up to the end of Year 2. The responses from FY1 doctors reflected the teaching they received throughout their whole undergraduate education (Fig. S1). Students in an intercalated year provided data reflecting the teaching they had received up until the most recent year of completion. We considered the first year of graduate‐entry medical courses to equal the first 2 years of their undergraduate‐entry equivalent (Fig. S1). This enabled the differentiation between the early years (1–2) and clinical stages (3–5) of undergraduate education.

Collaborators at each medical school were recruited through a targeted advertising drive using social media, medical school societies, websites, and newsletters. Each collaborator recruited further survey respondents from their university to complete the survey. We uploaded each university’s specific curriculum to the survey, which participants were prompted to view before completing the survey.

As per UK NHS Health Research Authority guidance, NHS Research Ethics Committees review exemption applied. By partaking in the survey, participants consented to the use of their data for the purpose of the study. Participants had the option to withdraw their consent at any point by contacting the study team.

### Inclusion and Exclusion Criteria

Survey completion was voluntary and open to any medical student at any medical school in the UK, and any FY1 doctor who graduated from a UK medical school, that performs under the GMC’s and Medical School Council’s guidelines.

Year 1 students were excluded as they would have not yet received a full year of teaching. Students who studied or graduated from a medical school outside of the UK and FY2 doctors or more senior were also excluded. Two universities were excluded as they were new medical schools starting in the 2020–2021 year, and therefore only had a Year 1 cohort (Table S1). All responses were screened for inclusion eligibility by the LEARN Steering Committee.

### Outcomes

The primary outcome measure was the individual proportion of topics in the BAUS undergraduate syllabus covered by medical schools, per year group, across the UK. Secondary outcomes included the type and quantity of teaching provided, the reported performance rates of GMC‐mandated urological procedures, and the proportion of those who had considered a career in urology and the impact of the severe acute respiratory syndrome coronavirus 2 2019 (COVID‐19) pandemic on teaching. Quantity was determined per ‘teaching session’, which was defined as a lecture, objective structured clinical examination (OSCE) session, outpatient clinic, full theatre list, etc. Detailed secondary outcomes measured in this analysis are listed in Table S2.

### Statistical Analysis

Following data cleaning, the results were reported both quantitatively and qualitatively where appropriate. Descriptive comparison of outcomes were made between the FY1 year group and other year groups, to observe trends in the progression of teaching and exposure to urology. All statistical analyses were performed using Stata MP/16.0 (Stata Corp, College Station, TX, USA) and Microsoft Excel (Microsoft Corp., Redmond, WA, USA).

## Results

### Demographics

**Fig. 1:**
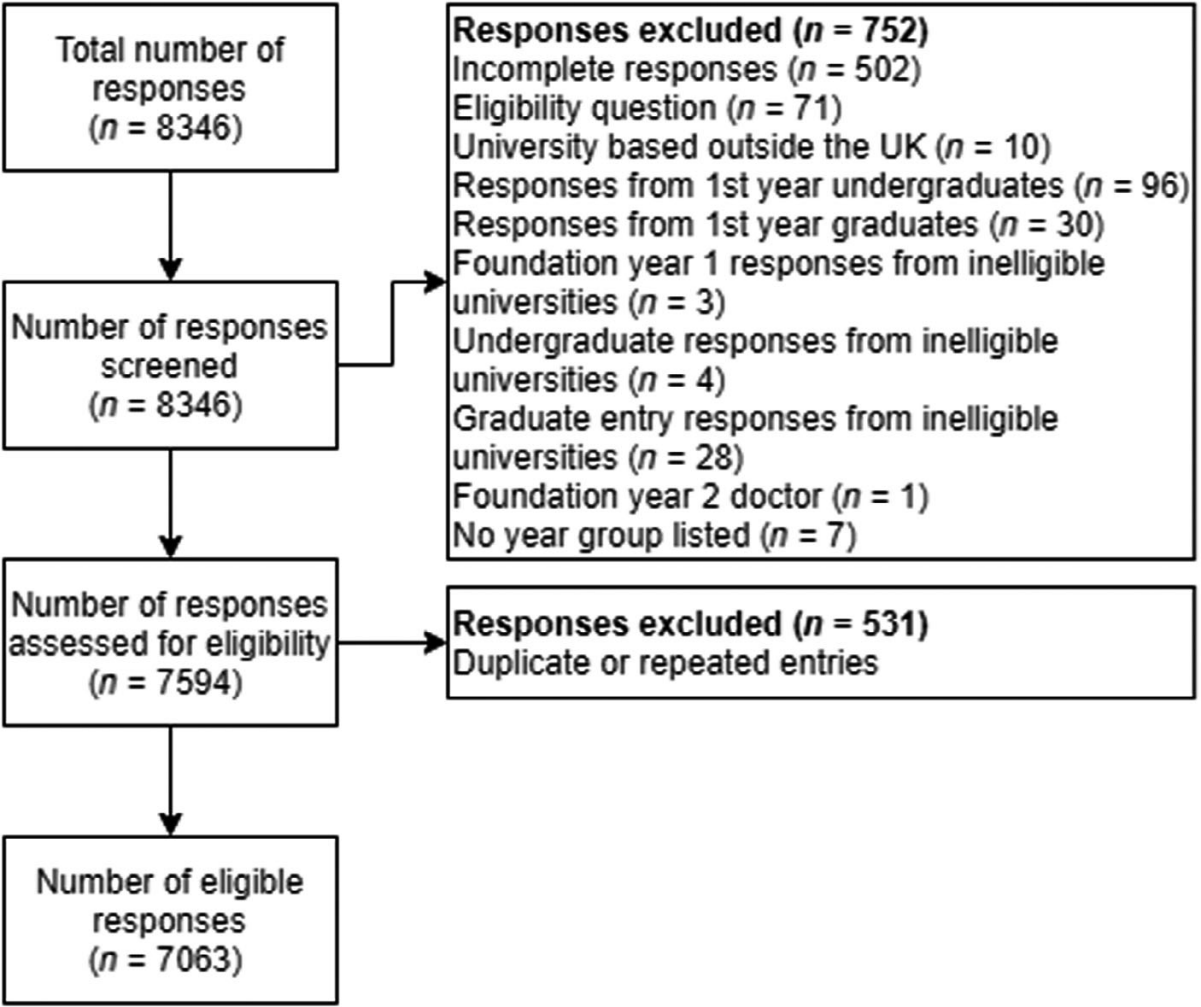
Cohort flow diagram.

Of an estimated 40 927 UK Year 2–Year 5 medical students and FY1 doctors, a total of 8346 survey responses were received, of which 7063 (84.6%) met the eligibility criteria (Fig. 1) [18]. Responses were received from all 39 eligible UK medical schools out of 41 nationally (Fig. S2). The median (interquartile range [IQR]) number of responses per medical school was 158 (90, 249). All year groups included in the study were well‐represented (Fig. S3). There were 1127/7063 (16.0%) FY1 responses.

### BAUS‐recommended Undergraduate Teaching Topics

Table 1 shows the proportion of BAUS‐recommended teaching topics covered in each year group. Amongst FY1 doctors, the three most taught topics were UTI (96.5%, 1088/1127), acute kidney injury (95.9%, 1081/1127) and haematuria (94.4%, 1064/1127), whilst the three least taught topics were male urinary incontinence (59.4%, 670/1127), male infertility (52.4%, 591/1127) and erectile dysfunction (43.8%, 494/1127). In all, 0.8% of FY1 doctors reported receiving no urology teaching during medical school. The proportion of teaching topics covered increased with progression through medical school, and generally peaked by Year 5.

**Table 1:** The reported rate of teaching topics covered, as recommended by the BAUS undergraduate syllabus, stratified by year group. The topics have been sorted from highest to lowest percentage within the FY1 year. Percentage (%) corresponds to the ‘Yes’ value. *N* is the number of students reporting in that year.

Teaching topic, %	Year 2 (*n* = 1343)	Year 3 (*n* = 1791)	Year 4 (*n* = 1870)	Year 5 (*n* = 932)	FY1 (*n* = 1127)
UTI	61.4	88.2	95.6	98.8	96.5
Acute kidney injury	51.2	84.8	94.3	96.6	95.9
Haematuria	49.7	79.6	93.7	96.0	94.4
Male LUTS	34.4	72.0	90.2	93.3	92.9
Acute renal tract stone disease	31.0	58.1	84.3	91.8	92.4
Urological cancer	26.7	58.4	80.1	92.5	89.4
Female LUTS	34.3	70.6	88.0	90.3	89.0
Female urinary incontinence	29.3	55.8	69.2	88.9	85.6
Scrotal swelling and pain	16.0	43.3	70.7	83.3	84.2
Paediatric urology conditions	12.8	29.9	49.5	78.1	80.6
Abdominal pain referable to the urinary tract	39.2	64.6	80.4	81.5	77.4
Male urinary incontinence	30.9	58.6	66.7	67.6	59.4
Male infertility	20.5	40.0	38.8	53.5	52.4
Erectile dysfunction	16.2	37.4	43.1	50.5	43.8
None	19.2	3.5	1.2	0.6	0.8

### BAUS‐recommended Undergraduate Observed Surgical Procedures

The overall reported rates of observed urological procedures were low across all year groups (Table 2). The reported rates of undergraduate observed urological procedures were highest for laparotomy (51.6%, 582/1127), flexible cystoscopy (50.8%, 572/1127) and TURP (35.8%, 403/1127) for FY1 doctors (Table 2), whilst the lowest reported rates were scrotal surgery (17.7%, 199/1127), CT urogram (9.9%, 112/1127) and IVU (7.5%, 85/1127). The proportion of students having observed urological procedures increased with progression through medical school, peaking by Year 5.

**Table 2:** The reported rate of observed urological procedures, as recommended by the BAUS undergraduate syllabus, stratified by year group. The procedures have been sorted from highest to lowest percentage within the FY1 year.

Urological procedure, %	Year 2 (*n* = 1343)	Year 3 (*n* = 1791)	Year 4 (*n* = 1870)	Year 5 (*n* = 932)	FY1 (*n* = 1127)
Laparotomy – observed	5.0	11.4	28.8	49.5	51.6
Flexible cystoscopy – observed	2.1	9.2	28.9	53.4	50.8
TURP – observed	0.9	3.9	17.0	35.9	35.8
Rigid cystoscopy – observed	1.2	4.3	13.5	32.3	29.3
TURBT – observed	1.1	3.5	12.1	26.2	25.1
Urodynamics – observed	1.9	3.5	9.0	24.5	24.8
CT urogram – interpreted and discussed with supervision	5.3	7.7	17.0	23.3	23.7
Ureteroscopy – observed	1.4	4.0	12.8	24.7	20.9
Voiding flow rate – interpreted with supervision	1.3	3.0	9.2	22.2	20.8
TRUS ± prostate biopsy – observed	1.7	3.4	11.4	22.9	20.4
Circumcision – observed	1.9	3.1	7.2	15.6	18.6
Suprapubic catheter insertion or change – observed	2.5	4.6	8.1	11.5	18.5
Scrotal surgery – observed	1.0	2.1	8.0	16.2	17.7
CT urogram – observed	8.9	7.7	11.1	11.8	9.9
IVU – interpreted and discussed with supervision	2.4	1.6	6.6	10.4	7.5

iTURBT, transurethral resection of bladder tumour. Percentage (%) corresponds to the ‘Yes’ value. *N* is the number of students reporting in that year.

### BAUS‐recommended Undergraduate Observed and Performed Clinical Skills Procedures

In their undergraduate training, 79.9% (901/1127) of FY1 doctors reported having observed a male genital examination with 65.2% (735/1127) reporting having performed one on a patient, and 11.5% (130/1127) reporting having never performed one on either a model or a patient (Table 3).

**Table 3:** The reported observation and performance rates of practical procedures, as recommended by the BAUS undergraduate syllabus, stratified by year group.

Practical procedure, %	Year 2 (*n* = 1343)	Year 3 (*n* = 1791)	Year 4 (*n* = 1870)	Year 5 (*n* = 932)	FY1 (*n* = 1127)
Male genital examination – observed	12.43	19.04	40.43	71.78	79.95
Male genital examination – never performed	83.32	73.93	47.81	25.11	11.54
Male genital examination – performed only on a model	16.60	23.51	40.43	37.77	23.25
Male genital examination – performed only on a patient	0.07	0.95	3.48	8.15	11.54
Male genital examination – performed on both a model and a patient	0.00	1.62	8.29	28.97	53.68
DRE – observed	20.70	35.68	68.93	90.67	96.81
DRE – never performed	78.78	55.50	18.18	6.33	0.98
DRE – performed only on a model	20.63	39.98	56.79	35.94	4.79
DRE – performed only on a patient	0.30	1.40	2.19	4.51	6.48
DRE – performed on both a model and a patient	0.30	3.13	22.83	53.22	87.76
Male catheterisation – observed	13.55	27.81	57.70	84.44	96.63
Male catheterisation – never performed	95.46	74.26	19.84	5.36	0.89
Male catheterisation – performed only on a model	4.02	22.50	62.89	40.88	7.01
Male catheterisation – performed only on a patient	0.45	0.67	0.70	2.04	2.93
Male catheterisation – performed on both a model and a patient	0.07	2.57	16.58	51.72	89.17
Female catheterisation – observed	9.61	17.87	44.65	80.58	86.96
Female catheterisation – never performed	97.54	82.02	40.37	12.23	6.83
Female catheterisation – performed only on a model	2.16	16.25	45.78	29.18	20.14
Female catheterisation – performed only on a patient	0.22	0.11	2.30	9.44	8.16
Female catheterisation – performed on both a model and a patient	0.07	1.62	11.55	49.14	64.86

Percentage (%) corresponds to the ‘Yes’ value. *N* is the number of students reporting in that year.

Overall, 96.8% (1091/1127) of FY1 doctors reported having observed a DRE, 94.2% (1062/1127) reported having performed one on a patient, but 1.0% (11/1127) reported having never performed one on either a model or a patient (Table 3).

As a percentage of FY1 doctors, 96.6% (1089/1127) and 87.0% (980/1127) had observed male and female catheterisation respectively, 92.1% (1038/1127) and 73.0% (823/1127) had performed one on a male and female patient respectively, whilst 0.9% (10/1127) and 6.8% (77/1127) had never performed male and female catheterisation respectively, on either a model or patient (Table 3).

The median (IQR) number of male and female catheterisations performed by FY1 doctors was 4 (2, 6) and 2 (1, 3), respectively (Figs S4 and S5). The number of catheterisations performed increased with progression, peaking by FY1 for male catheterisation, and by Year 4 for female catheterisation. By the end of medical school, the median number of female catheterisations performed was half of the median number of male catheterisations performed.

### Type and Quantity of Theory‐based Teaching

FY1 doctors reported receiving theory‐based teaching through lectures (92.6%, 1044/1127), small group tutorials (59.7%, 673/1127), anatomy demonstration/dissecting room (54.1%, 610/1127) and problem‐based learning groups (35.0%, 395/1127) (Fig. S6). Overall, 2.1% (149/7063) reported receiving theory‐based teaching on clinical placements, in operating theatres, and clinical skills sessions, as well as through self‐directed teaching.

The median (IQR) number of FY1 reported cumulative theory‐based teaching sessions (defined as a lecture, small group tutorial or problem‐based learning session) delivered in medical school was 8 (5, 12) (Fig. S7). This did not increase beyond Year 3.

### Type and Quantity of Clinical Skills Teaching

As a percentage of FY1 doctors, the most commonly reported methods of undergraduate clinical skills teaching were OSCE (65.0%, 732/1127), patient‐bedside teaching (57.5%, 648/1127), and tutorials/video‐based teaching (45.6%, 514/1127) (Fig. S8). Overall, 0.4% (31/7063) reported having received clinical skills teaching through simulation using models, lectures, clinical placement observation, and in the laboratory setting. Overall, 11.9% (134/1127) of FY1 doctors reported not having received any urology‐specific clinical skills teaching. The proportion of students receiving clinical skills teaching increased sharply after they commenced the clinical phase of medical school and peaked by Year 5.

The median (IQR) number of cumulative clinical skills sessions (defined as an OSCE session or being supervised practising a clinical skills examination on a patient) reported by FY1 doctors was 3 (2, 5) (Fig. S9), an increase from a median of 2 in Years 2 and 3.

### Undergraduate Clinical Attachment in Urology

Amongst FY1 doctors, the most common types of clinical exposure in urological attachments were ward‐based (64.2%, 723/1127), followed by outpatients (50.4%, 568/1127), main operating theatres (43.7%, 493/1127), and day case surgery (36.1%, 407/1127) (Fig. S10). Overall, 0.6% (40/7,063) reported receiving urology training during their attachment in other settings, such as primary care, the emergency department, and medical elective placements. Overall, 20.1% (227/1127) of FY1 doctors reported not having had any form of clinical attachment in urology whilst in medical school. Nearly 70% reported not receiving a clinical attachment in urology in the first 2 years. The proportion of students receiving a clinical attachment increased with progression through medical school, and generally peaked by Year 5.

The median (IQR) number of urology clinical attachment sessions (defined as an outpatient clinic, teaching ward round or urology operating list session) reported by FY1 doctors was 6 (3, 10) (Fig. S11), increasing from a median of 3 in Year 3.

### Reported Urological Confidence

**Fig. 2:**
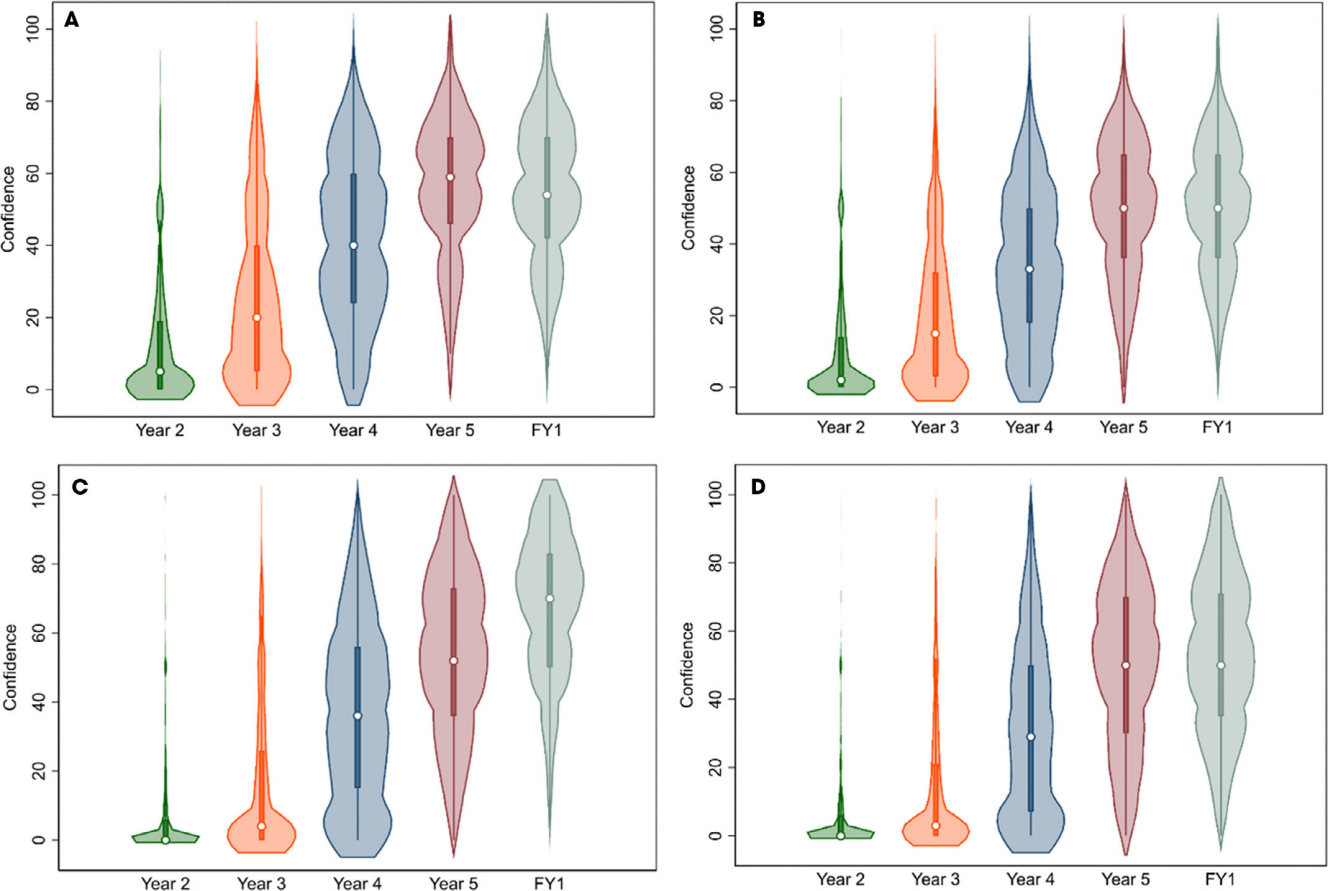
Self‐perceived confidence in (**A**) clerking and (**B**) managing a urology patient as would be expected of an FY1 doctor, and in inserting a (**C**) male and (**D**) female catheter, stratified by year group. Percentage (%) corresponds to the ‘Yes’ value. [Colour figure can be viewed at wileyonlinelibrary.com]

On a scale of zero to 100 (with 100 being extremely confident, 50 being neutral and zero being not confident at all), the median (IQR) self‐reported confidence by FY1 doctors in clerking a urological patient (history and examination) as would be expected of an FY1 doctor was 54 (43, 70) (Fig. 2A), in initiating management for common urological conditions was 50 (37, 65) (Fig. 2B), in inserting a male catheter was 70 (50, 83) (Fig. 2C), and in inserting a female catheter, 50 (35, 71) (Fig. 2D). It was observed that the median confidence in inserting a female catheter was consistently lower than that of a male catheter across all year groups.

### Impact of the COVID‐19 Pandemic

Overall, 35.1% (2481/7063) of students across all year groups reported their urology teaching to have been impacted by the SARS‐CoV‐2 (COVID‐19) pandemic. The least disruption in urology teaching was reported by FY1 doctors (15.8%, 178/1127) (Fig. S12). Of those impacted, clinical attachment and clinical skills teaching were most affected in clinical years, with the greatest disruption in Year 4 (92.0%, 743/808 and 79.2%, 640/808, respectively). Theory‐based teaching was most disrupted in Year 2 (72.4%, 393/543) but improved incrementally in more senior years (60.1% FY1, 107/178) (Fig. S13).

Of the original anticipated urology timetable, the median reported percentage delivered during the pandemic, in those affected, was 50% across all year groups. The greatest disruption was to Year 4 students where the median (IQR) reported percentage of teaching provided was 43% (25%, 52%) (Fig. S14). In all, 22.3% (554/2,481) of those affected reported not receiving any of their anticipated urology teaching during the pandemic (Fig. S15). The most utilised teaching modality across all year groups during the pandemic was E‐learning (67.0%, 1663/2481), followed by on‐line assignments (30.0%, 719/2481).

On a scale of zero to 100 (where 100 is extremely satisfied, 50 being neutral and zero is not at all satisfied), the self‐reported satisfaction of urology teaching provided during the pandemic across all year groups was 49 (Fig. S16). The lowest satisfaction was amongst FY1 doctors with a median (IQR) of 38 (20, 52).

### Self‐selected Urology Modules

Overall, 9.0% (637/7063) of students reported having taken a self‐selected urology module, of which the median (IQR) number across all year groups was 1 (1, 2) (Fig. S17).

### Postgraduate Career in Urology

**Fig. 3:**
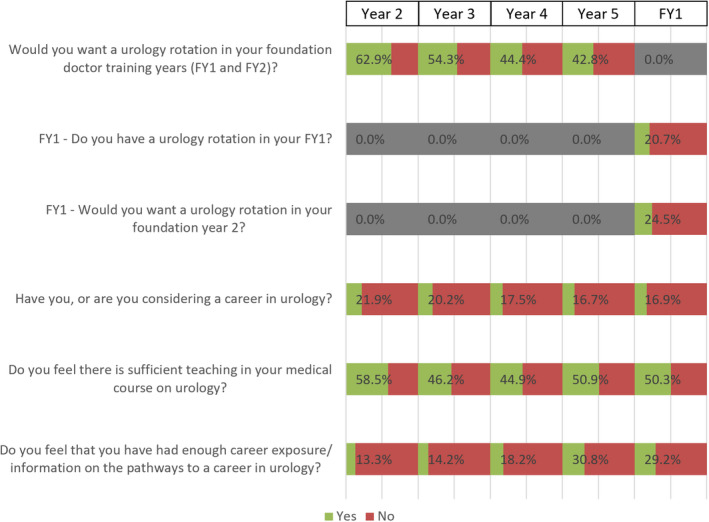
Postgraduate career and exposure, stratified by year group. Percentage (%) corresponds to the ‘Yes’ value. [Colour figure can be viewed at wileyonlinelibrary.com]

With progression through the years, there appeared to be a declining interest in urology as a career (Fig. 3). Overall, 62.9% (845/1343) of Year 2 students reported wanting a urology rotation during their FY programme, decreasing to 42.8% in Year 5 (399/932); 21.9% (294/1343) of Year 2 students reported having considered a career in urology, decreasing to 16.9% (190/1127) of FY1 doctors. During their foundation programme, 20.7% (233/1127) of FY1 doctors had a urology rotation during their FY1, and 24.5% (276/1127) wanted a urology rotation during their FY2. Overall, 50.3% (567/1127) of FY1 doctors stated that there was sufficient teaching in their medical course on urology; and 29.2% (329/1127) stated that they had enough career exposure/information on the pathways to a career in urology.

### Overall Comparison Between Medical Schools

The variation in achieving some of the key teaching topics taught, and urological procedures observed and performed between medical schools can be found in the supplementary appendix (Fig. S18).

## Discussion

Our study has shown that students become more competent and confident in urology knowledge and skills during their senior years of training. Theory‐based and clinical skills teaching are mainly delivered during early years (1–2), whilst clinical attachments are mainly undertaken during the clinical years. However, 7.9% and 27.0% of FY1 doctors report not having performed catheterisation on male and female patients respectively, a basic urological skill mandated by the GMC. Poorly taught topics included male urinary incontinence, male infertility, and erectile dysfunction, whilst students were well taught in UTI, acute kidney injury and haematuria. These results reflect the perceived confidence of students to manage urological conditions as a junior doctor. Other opportunities like urology self‐selected modules were rare.

The key strength of our study includes its multicentre nature and large sample size, including a response from 16.4% of all newly qualified FY1 doctors (2020 graduates, 1127/6889), and 17.3% of all Year 2 to Year 5 medical students and FY1 doctors collectively (7063/40 927). In addition, representation was achieved across all 39 eligible UK medical schools. This enabled us to capture the heterogeneity in placement exposure, both across and within UK medical schools. To our knowledge, this is the largest study on specialty‐specific undergraduate teaching. We did not find any comparable large‐scale evidence on urology education during medical school in other countries. This makes the LEARN the only study worldwide to investigate this and serves as a good baseline for other countries to investigate their undergraduate urology exposure. Additionally, there was no strong evidence to suggest significant variation in results between medical schools based on the number of responses (Fig. S18). Medical schools with a greater number of responses tended to fall within a reasonable difference to the national average, whilst medical schools with a lower number of responses were at risk of over‐estimating urological exposure.

A national curriculum is essential to guide medical schools in providing a minimum standard and quality of undergraduate urology teaching. Whilst no European‐wide undergraduate curriculum currently exists, there is the AUA Medical Students Curriculum, BAUS Undergraduate Syllabus for Urology, and incoming GMC Medical Licensing Assessment (MLA) syllabus [5, 19]. In addition, work is underway to address this on a European‐wide scale via the European Association of Urology.

We observed that a wide variety of teaching topics were covered across all year groups; however, some doctors are still graduating without being taught in many common urological presentations such as UTI, acute kidney injury and haematuria. This is concerning considering the prevalence of urological conditions that present in primary and secondary care.

There is a broad consensus from national governance bodies such as the BAUS, GMC and Association of Surgeons in Training that newly‐qualified doctors should be competent in both male and female catheterisation, with the GMC mandating that ‘newly qualified doctors will have performed the procedure on real patients during medical school’ under direct supervision [2, 12, 20]. Our data shows that performance of catheterisation in our cohort was higher than previously reported UK performance rates of 60% and 36%–40% for male and female catheterisation, respectively [21, 22]. However, performance rates of female catheterisation remain consistently low compared to male catheterisation, perhaps reflecting the lower perceived confidence in performing female catheterisation that we found. This suggests that a substantial proportion of newly qualified doctors have not met this clinical competency required by the GMC, and this lack of experience may contribute to catheter‐associated iatrogenic injury, urethral stricture disease, and poor patient experience [23]. Furthermore, the management of a patient with acute urinary retention is a common presentation that all doctors will face, regardless of future specialty. However, it should be noted that female catheterisation is performed more commonly by nursing staff, and as medical students are more likely to be observing doctors, they are less likely to observe, and therefore perform female catheterisation.

Our results have identified areas of urology that are under‐represented in urology teaching. The lower observation and performance rates of male genital examination across all year groups is concerning as the acute painful scrotum is a surgical emergency. We found a higher performance rate (94.2%) of DRE by FY1 doctors than previously reported (86%) [22]. Whilst neither a DRE or male genital examination are GMC‐mandated requirements, these examinations, and knowledge of common presentations such as male urinary incontinence, erectile dysfunction and prostate cancer are important examinations and presentations within Primary Care [24]. However, it should be noted that the lower performance rates might be the result of male genial examination and DRE both being intimate examinations, and therefore reluctance on the part of students and patients to participate.

We have identified a need for a greater emphasis on clinical exposure to urology. Overall, 20.1% of FY1 doctors report not having had a clinical attachment, thereby reducing the opportunity to observe surgical procedures and perform practical skills on patients. Furthermore, the incoming GMC MLA includes urological conditions and presentations within it. Increasing the number of clinical skills sessions may help to improve confidence in practical skills such as catheterisation.

It has been suggested that important influencing factors in pursuing a future career in urology include early introduction of the specialty, the duration of clinical exposure and teaching, and the time spent conducting practical procedures [8]. It has also been reported that students receive on average 1 week of clinical urology experience [9], and this may contribute to our finding of self‐reported dissatisfaction at the duration of urology placements. Other evidence from UK studies report 90.7% of junior doctors felt their undergraduate teaching in urology was suboptimal, 68.9% stated there should be more dedicated urology teaching time in medical school, and 87.5% felt they had not been exposed to enough urology as an undergraduate [21, 25]. Our data show some improvement from these, with 49.7% of FY1 doctors reporting their urology teaching was insufficient and 70.8% reporting insufficient career exposure. However, with limited clinical urological exposure, interest rates in urology remain low. Our findings show the overall interest rate in urology (16.9% amongst FY1 doctors and 18.8% across all participants) is similar to previous studies reported at 14%–15.6% [11, 21, 26]. This may contribute to the declining competition ratios for specialty training in urology from nearly 18:1 in 2007 to just over 2:1 in 2020, with urology presenting the second lowest competition ratio amongst all the surgical specialties in 2020 [27, 28]. Additionally, the opportunities to pursue an interest in urology during medical school may be limited, with only 9.0% of all students reporting having undertaken a urology SSC, despite 18.8% of all participants expressing an interest in urology.

The COVID‐19 pandemic has had a profound effect on medical education. Almost all clinical attachments were cancelled in the first wave, and most undergraduate urology teaching was therefore necessarily delivered in an on‐line format. The apprenticeship model only resumed when in‐person placements restarted. We found that most theory‐based teaching sessions are delivered in the early years, whilst clinical exposure is delivered during the clinical years. It follows therefore that a greater proportion of early year students reported disruption to their theory‐based teaching, whilst a greater proportion of clinical year students reported disruption to their clinical skills teaching and clinical attachment. This may also reflect the FY1 doctor cohort reporting the least disruption to their training during the pandemic, yet the greatest dissatisfaction with the teaching provided during the pandemic. This may reflect that theory‐based teaching is provided earlier on in their course, but also the need to perform clinical procedures during their later and final years in preparation for OSCE examinations and in meeting clinical skills competencies. Whilst the reported satisfaction of teaching provided during the pandemic remains neutral, we are unable to comment on how this compares to the satisfaction of teaching provided pre‐pandemic. Parallels can be drawn between the effects of the pandemic on undergraduate training and on training for urology trainees. Both cohorts have experienced significant cancellations and a shift towards virtual teaching and consultations [29], which has affected opportunities for individuals to meet their required competencies.

Limitations of the study include the retrospective nature of the evaluation invoking recall bias in participants. To minimise this, data were collected at the start of the academic year and participants were prompted to view their curriculum before completing the survey. Further limitations included selection bias, as our survey respondents were likely to be inherently interested in surgery or urology, although we observed that only 18.8% of all respondents have considered a career in urology. This may influence the results by overestimating the urological exposure that medical students may have had. Additionally, the impact of COVID‐19 on placement exposure may have affected the proportion of those considering urology as a career.

The LEARN Study provides the most thorough national evaluation of urology teaching to date. It has identified areas where urology teaching is satisfactory, and areas for improvement, such as in teaching of common presentations, and in practical skills. It has also identified that overall, exposure to urology and interest in the specialty is better than previous UK reports.

Our data have identified areas in which the BAUS syllabus may be updated. It has identified procedures, such as IVU, which may now be outdated and infrequently used in clinical practice. Additionally, with the advancements in minimally invasive surgery in urology over the past decade [30], observation of laparoscopic and robotic procedures could be included in an updated syllabus. There are now plans to collaborate with BAUS to create an updated undergraduate syllabus, which will be used to lobby medical school curriculum leads to update their Urological teaching.

Future work should focus on identifying reasons for the differences in urology exposure, e.g., why some students report not having the opportunity to catheterise. Further qualitative research is required to explore these factors, and subsequently allow students to identify how best to optimise their urology exposure during medical school.

Whilst the medical school curriculum is already overloaded, and with each specialty competing for increased exposure, there are many aspects of urology that should be deemed crucial, including catheterisation. Placement exposure and experience should reflect the prevalence of urology as a specialty, and of urological conditions: one in five FY1 doctors have a urology rotation; one in five acute surgical referrals are due to a urological condition; and one in five NHS hospital inpatients will have a urinary catheter inserted at any given time [3, 4].

## Conclusion

To our knowledge, this study is the largest evaluation of urology teaching ever performed. Overall, urology teaching seemed satisfactory as evaluated by the BAUS undergraduate syllabus and seemed to have improved compared to previous reports. However, we have identified areas needing development, such as performed catheterisation rates, where procedural experience on patients is a GMC requirement. The COVID‐19 pandemic seemed to have negatively affected urology teaching across all year groups. The results of this study should promote engagement with medical schools to support changes to the curriculum and enable a re‐evaluation of the BAUS syllabus, considering changes to practice over the past decade and more recent changes in on‐line delivery of medical education.

## Author Contributions

Sinan Khadhouri and Veeru Kasivisvanathan were responsible for the study idea. Keerthanaa Jayaraajan, Alexander Ng, Vinson Wai‐Shun Chan, Aqua Asif, Nikita R. Bhatt, and Sinan Khadhouri developed the concept. Alexander Ng, Vinson Wai‐Shun Chan, Aqua Asif, Nikita R. Bhatt, Sinan Khadhouri, and Veeru Kasivisvanathan were responsible for the study design. Alexander Ng, Vinson Wai‐Shun Chan, Aqua Asif, Alexander Light, Chon Meng Lam, Keerthanaa Jayaraajan, William A. Cambridge, Meghana Kulkarni, Nikita R. Bhatt, Sinan Khadhouri, and Veeru Kasivisvanathan were responsible for co‐ordinating the study. Alexander Ng, Vinson Wai‐Shun Chan, Aqua Asif, Nikita R. Bhatt, Sinan Khadhouri, and Veeru Kasivisvanathan were responsible for data quality assurance. Alexander Ng, Vinson Wai‐Shun Chan and Aqua Asif were involved in data cleaning and statistical analysis. Alexander Ng wrote the first draft of the manuscript. All mainline authors were involved in the interpretation, editing, critical review, and final approval of the manuscript.

## Disclosure of Interest

The authors of this manuscript have no conflicts of interest to declare.

## Supporting information


**Figure S1** Retrospective analysis.
**Figure S2** The number of responses, stratified by medical school and year of study.
**Figure S3** Distribution of responses, stratified by year group.
**Figure S4** Cumulative number of male catheterisations performed, stratified by year group.
**Figure S5** Cumulative number of female catheterisations performed, stratified by year group.
**Figure S6** Type of theory‐based teaching, stratified by year group.
**Figure S7** Cumulative number of theory‐based teaching sessions, stratified by year group.
**Figure S8** Type of clinical skills teaching, stratified by year group.
**Figure S9** Cumulative number of clinical skills sessions, stratified by year group.
**Figure S10** Type of clinical attachment, stratified by year group.
**Figure S11** Cumulative number of clinical attachment sessions, stratified by year group.
**Figure S12** Impact of COVID‐19 since March 2020 on urology teaching, stratified by year group.
**Figure S13** Of those impacted by COVID‐19, the area of impacted urology teaching, stratified by year group.
**Figure S14** Of those impacted by COVID‐19, the reported percentage of original anticipated urology timetable before COVID‐19 delivered during the pandemic, stratified by year group.
**Figure S15** Of those impacted by COVID‐19, how the impacted urology teaching was provided during the COVID‐19 pandemic, stratified by year group.
**Figure S16** Of those impacted by COVID‐19, the reported satisfaction of impacted urology teaching provided during the COVID‐19 pandemic by year group.
**Figure S17** Number of self‐selected urology modules (e.g. special study components/ modules) completed by students (*n* = 637) who undertook one during medical school, stratified by year group.
**Figure S18** Cross medical school variation in selected procedures performed, key topics taught and key urological procedures observed.
**Table S1** Inclusion and exclusion criteria.
**Table S2** Secondary outcomes in the study.
**Appendix S1** PubMed Indexed Collaborators (BURST Collaborative LEARN Study Group).
**Appendix S2** LEARN Questionnaire.
**Appendix S3** Checklist for Reporting Results of Internet E‐Surveys (CHERRIES).
**Appendix S4** LEARN Protocol.
**Appendix S5** List of Survey Respondents.

## Appendix A

PubMed Indexed Collaborators (BURST Collaborative LEARN Study Group)

Ahaan Sanjay Gupta, Christopher Khoory, Owain Ellis, Maiar Elhariry, Lucia Harley, Viraj Shah, Hamza Umar, Reeshma Jameel, Jade Sangha, Miranda Ntorinkansah, Natalia Chilal, Anna Marshall, Balamrit Singh Sokhal, Vishal Chandanani, Caroline Jarman, Aishwarya Sharma, Hasti Tarzban, Maram Nabahin, Lewis Bonsell, Benjamin Langhorne, Prachi Agarwal, Rajwant Kaur, Alexander Hunt, Cecilia Cirelli, Natasha Alford, Nesta Baxter, Anneesa Malik, Abigail Harrison, Monty Matson, Ronak Shah, Cliona Meenan, Daanish Ghaffar, Katherine Wise, Lauren Gurr, Tahmeed Ahmed, Alice Jones, Ankur Singh, Chloe Stevens, Lien Salcedo, Michael Cooke, Sunwoo Lee, Amanda Godoi, Bethany Rose, Oluwajenrola Arawole, Sanjana Ilangovan, Alexander West, Alna Dony, Anoop Sumal, Ariadni Papadopoulou, Matan Bone, Omar Haque, Pooja Patel, Rawan Al Dehailan, Robert Grogan, Brishti Debnath, Carina Synn Cuen Pan, Isabelle Shaw, Katie Tsang, Marcus Graham, Milad Parsi, Nidhi Agarwal, Sarah Pengelly, Shabnam Tariq, Sumbal Bhatti, Greta Safoncik, Holly Thompson, Italia Rosa‐Leech, Maia Osborne‐Grinter, Matthew Hennessy, Ramisha Basharat, Rebecca Paterson, Sherie George, Shubham Gupta, Victoria Porter, Ashley Soloman, Attika Chaudhary, Briony Seden, Eimad Basit, Grace Kettyle, Jesvin Sunny, Madhumita Kolluri, Precious Jolugbo, Syme Bhopal, Tzvi Reich, Alexander Davies, Eleanor Kissane, Elena Missir, Gareth Hutchinson, Jonathan Chua, Nicole Wang, Olivia Pestrin, Ryan Faulder, Thomas McLelland, Vikash Patel, Aimee Wilkinson, Aliraza Syed, Amrit Mann, Christina Huon, Elina Stokolova, Elisa Lau, Ella Hobbs, George Garratt, George Higginbotham, Harriet Flashman, Hassan Ismahel, Ivana Homerova, Marcus Boyd, Rafi Abdullah, Rebecca Lim, Rebecca Vitarana, Sasha Quarrington, Vaishali Kiridaran, Ziad Zeidan, Alexis Adam, Ananya Nair, Bernice Johal , Brooke Gerrie, Christopher Gunn, Cora Lowe, Dhikshitha Nagaraj, Eleanor Deane, Jamie McGinn, Jennifer Luu, Karan Sagoo, Katie Sharman, Laura Cunningham, Megan Scotcher, Meshva Amin, Natasha Aghtarafi, Ruth Goh, Shi Pei Loo, Tatiana Hamakarim, Timothy Ho, Xinyu Ye, Zain Islam, Zoe Zagorac, Abdal Zafar, Alisha Kanani, Armin Nazari, Daniel Sescu, Inas Alsuhaibani, Isabel Munden, Jasmine Pattarukuzhyil Jose, Mariella Fortune‐Ely, Michaela Rogers, Pratyush Pradeep, Stephanos Ghobrial, Thomas Hall, Yasmin Motarjemi, Aaron Campbell, Allen Royal, Anna Kruczynska, Aqeeb Mahmood, Ateeq Jamil, Boris Wagner, Caitlin Murphy, Catriona Walker, Dilen Parmar, Isabelle Mayne, Jason Armstrong, Laura Inglis, Lily Waltham, Natalie Ko, Rhiannon Kirk, Roshni Johnson, Ryan Turner, Serena Patel, Sita Asi, Veena Sudarshan.
