# Supplementary material for: LEARN: a multicentre, cross‐sectional evaluation of Urology teaching in UK medical schools
Source: BJU Int. 2022 May 26;130(5):676–87. doi: 10.1111/bju.15758 (PMC9796355; doi:10.1111/bju.15758)
Supplement: Supplementary file 1 — Figure S1 Retrospective analysis. Figure S2 The number of responses, stratified by medical school and year of study. Figure S3 Distribution of responses, stratified by year group. Figure S4 Cumulative number of male catheterisations performed, stratified by year group. Figure S5 Cumulative number of female catheterisations performed, stratified by year group. Figure S6 Type of theory‐based teaching, stratified by year group. Figure S7 Cumulative number of theory‐based teaching sessions, stratified by year group. Figure S8 Type of clinical skills teaching, stratified by year group. Figure S9 Cumulative number of clinical skills sessions, stratified by year group. Figure S10 Type of clinical attachment, stratified by year group. Figure S11 Cumulative number of clinical attachment sessions, stratified by year group. Figure S12 Impact of COVID‐19 since March 2020 on urology teaching, stratified by year group. Figure S13 Of those impacted by COVID‐19, the area of impacted urology teaching, stratified by year group. Figure S14 Of those impacted by COVID‐19, the reported percentage of original anticipated urology timetable before COVID‐19 delivered during the pandemic, stratified by year group. Figure S15 Of those impacted by COVID‐19, how the impacted urology teaching was provided during the COVID‐19 pandemic, stratified by year group. Figure S16 Of those impacted by COVID‐19, the reported satisfaction of impacted urology teaching provided during the COVID‐19 pandemic by year group. Figure S17 Number of self‐selected urology modules (e.g. special study components/ modules) completed by students (n = 637) who undertook one during medical school, stratified by year group. Figure S18 Cross medical school variation in selected procedures performed, key topics taught and key urological procedures observed. Table S1 Inclusion and exclusion criteria. Table S2 Secondary outcomes in the study. Appendix S1 PubMed Indexed Collaborators (BURST Collaborative LEARN Study Group). Append [file BJU-130-676-s001.pdf]

Supplementary Appendix

This appendix has been provided by the authors to give readers additional information about their work.

Contents

Supplementary material 2
Figure S1: Retrospective analysis 2
Figure S2: The number of responses, stratified by medical school and year of study 3
Figure S3: Distribution of responses, stratified by year group 4
Figure S4: Cumulative number of male catheterisations performed, stratified by year group 5
Figure S5: Cumulative number of female catheterisations performed, stratified by year group 6
Figure S6: Type of theory-based teaching, stratified by year group 7
Figure S7: Cumulative number of theory-based teaching sessions, stratified by year group 8
Figure S8: Type of clinical skills teaching, stratified by year group 9
Figure S9: Cumulative number of clinical skills sessions, stratified by year group 10
Figure S10: Type of clinical attachment, stratified by year group 11
Figure S11: Cumulative number of clinical attachment sessions, stratified by year group 12
Figure S12: Impact of Covid-19 since March 2020 on urology teaching, stratified by year group 13
Figure S13: Of those impacted by Covid-19, the area of impacted urology teaching, stratified by year group 14
Figure S14: Of those impacted by Covid-19, the reported percentage of original anticipated urology timetable before Covid-19 delivered during the pandemic, stratified by year group 15
Figure S15: Of those impacted by Covid-19, how the impacted urology teaching was provided during the Covid-19 pandemic, stratified by year group 16
Figure S16: Of those impacted by Covid-19, the reported satisfaction of impacted urology teaching provided during the Covid-19 pandemic by year group 17
Figure S17: Number of self-selected urology modules (e.g. special study components/ modules) completed by students ($n = 637$) who undertook one during medical school, stratified by year group 18
Figure S18: Cross medical school variation in selected procedures performed, key topics taught and key urological procedures observed 19
Table S1: Inclusion and exclusion criteria 20
Table S2: Secondary outcomes in the study 21
Appendix 1: PubMed Indexed Collaborators (BURST Collaborative LEARN Study Group) 22
Appendix 2: LEARN Questionnaire 23
Appendix 3: Checklist for Reporting Results of Internet E-Surveys (CHERRIES) 35
Appendix 4: LEARN Protocol 37
Appendix 5: List of Survey Respondents 53

Copyright ©2022 BURST Research Collaborative

Supplementary material

Figure S1: Retrospective analysis

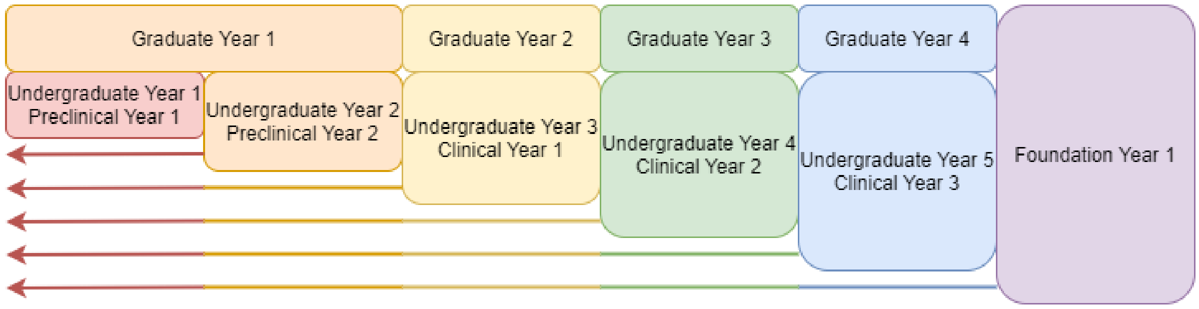

The survey retrospectively assessed the urology teaching that individuals had received to date. As such, Year 2 students' data reflect the teaching they received in Year 1, and Year 3 reflected teaching up to the end of Year 2, for example. The responses from FY1 doctors reflect the teaching they received throughout their whole undergraduate education. Students in an intercalated year provided data reflecting the teaching they had received up until the most recent year of completion. We considered the first year of graduate-entry medical courses to equal the first 2 years of their undergraduate-entry equivalent. This enabled the differentiation between he pre-clinical and clinical stages of undergraduate education.

Figure S2: The number of responses, stratified by medical school and year of study

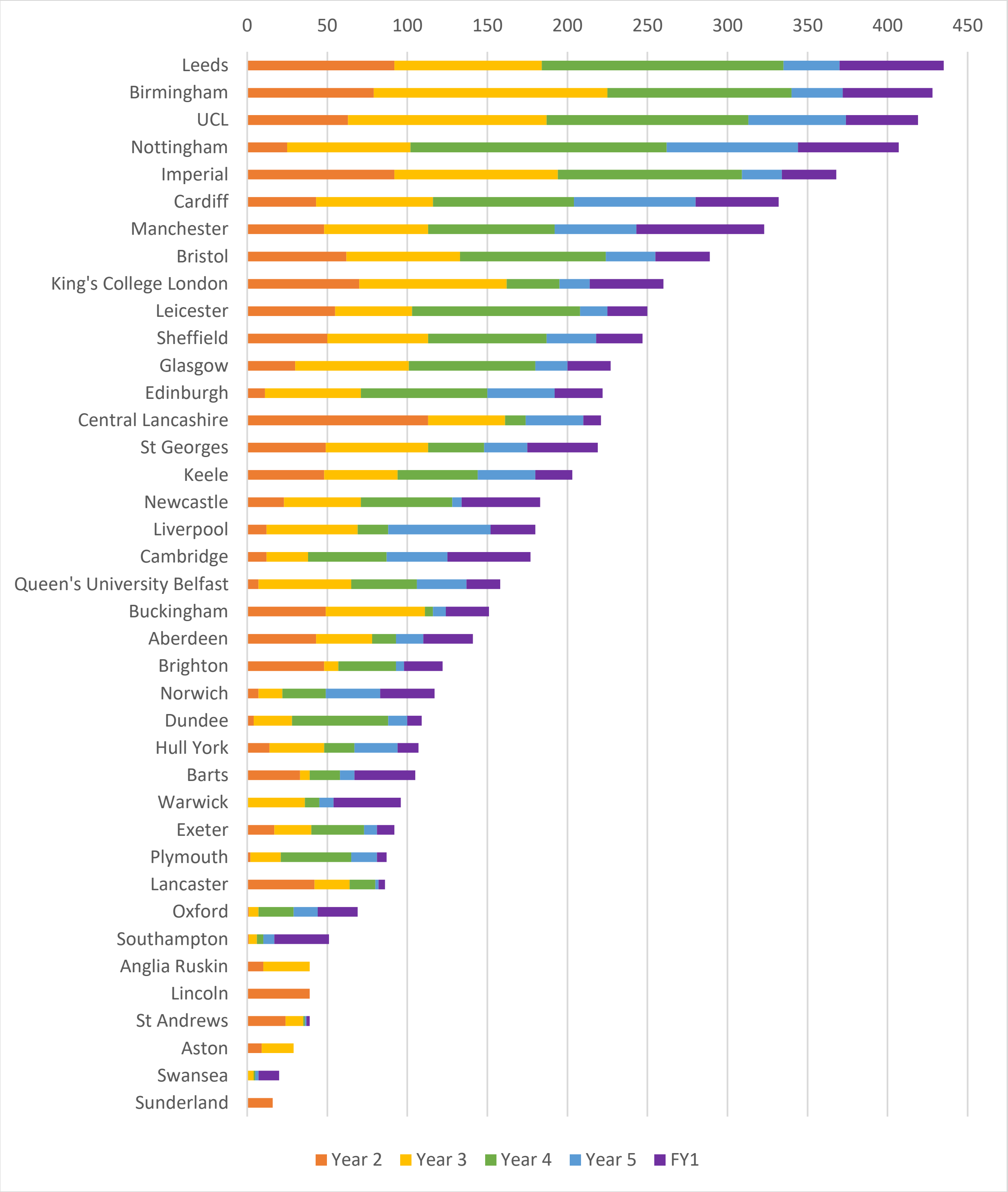

Copyright ©2022 BURST Research Collaborative

Figure S3: Distribution of responses, stratified by year group

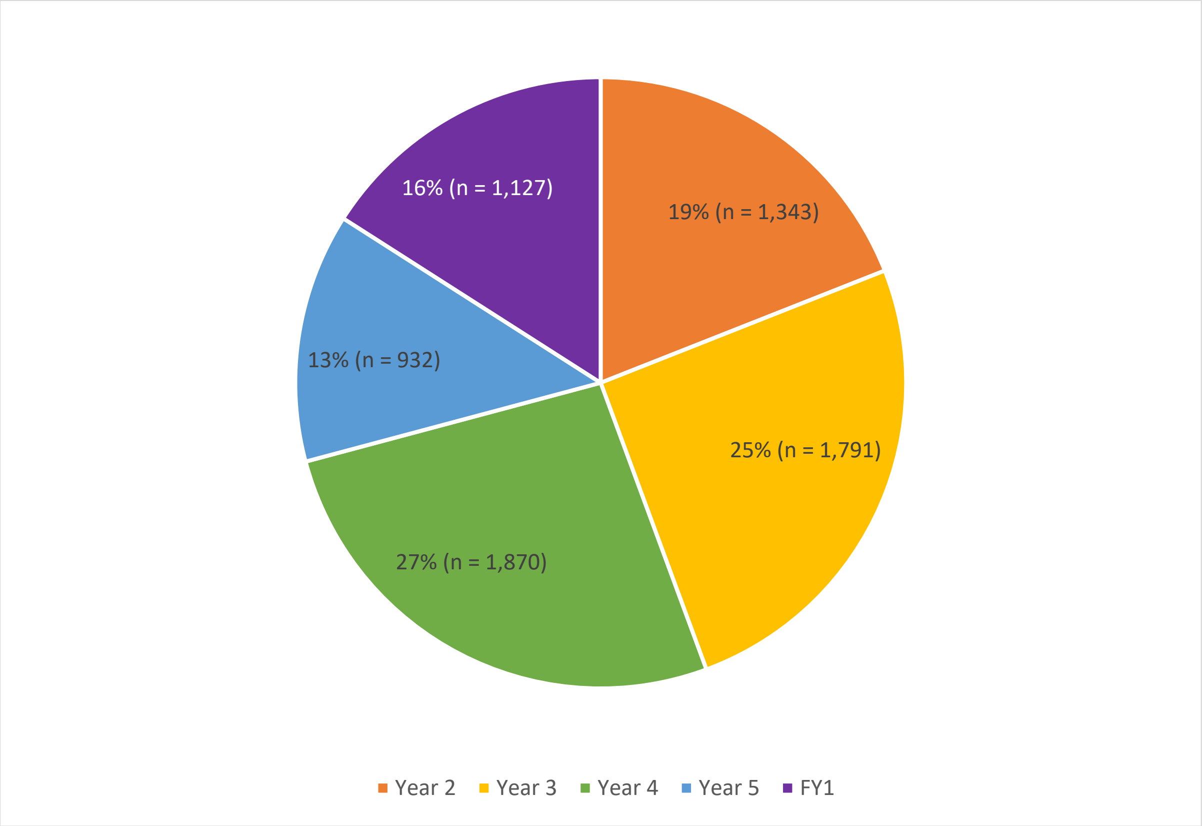

Figure S4: Cumulative number of male catheterisations performed, stratified by year group

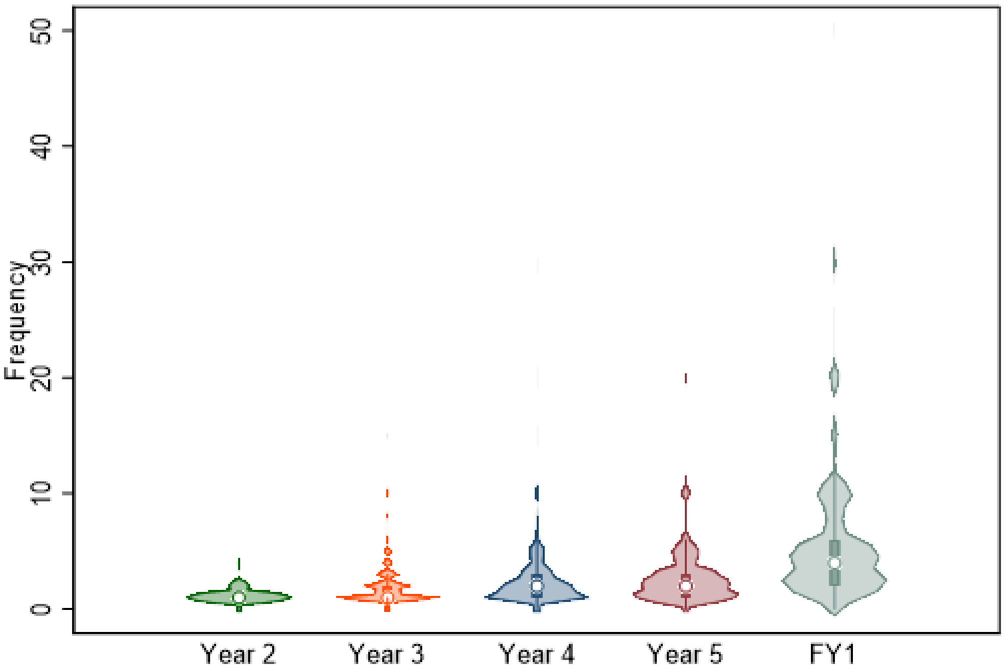

The white dot represents the median value. The coloured bars either side of the median represent the interquartile range (IQR). The vertical lines stretched from the IQR represent the lower and upper adjacent values, defined as $1st\ quartile - 1.5(IQR)$ and $3rd\ quartile + 1.5(IQR)$ respectively. Values lying beyond the lower and upper adjacent values are considered outliers. The width of each respective plot represents the density plot of the population data.

Copyright ©2022 BURST Research Collaborative

Figure S5: Cumulative number of female catheterisations performed, stratified by year group

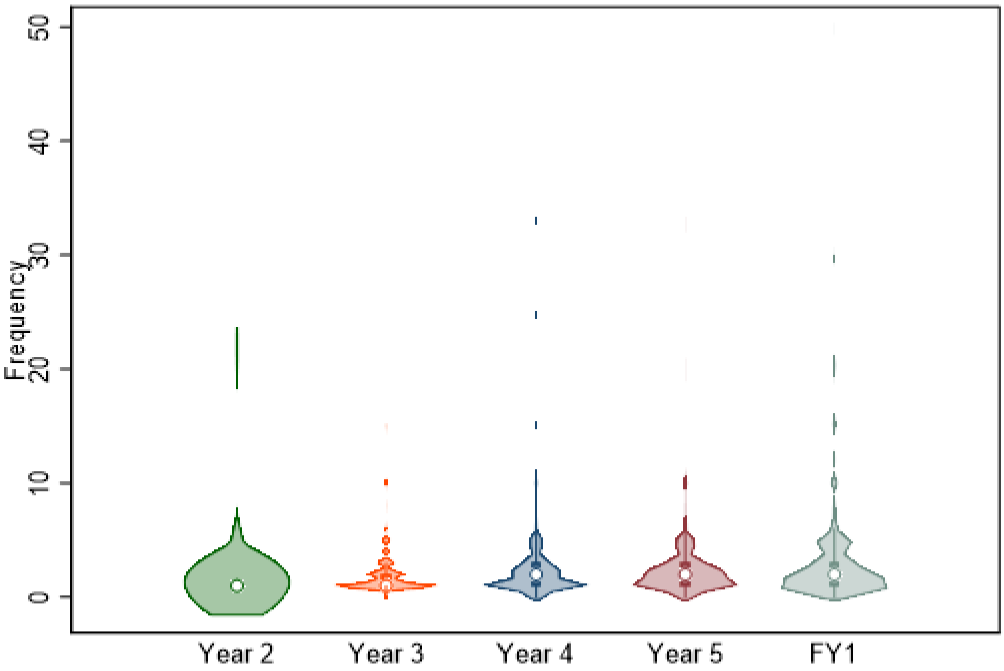

The white dot represents the median value. The coloured bars either side of the median represent the interquartile range (IQR). The vertical lines stretched from the IQR represent the lower and upper adjacent values, defined as $1st\ quartile - 1.5(IQR)$ and $3rd\ quartile + 1.5(IQR)$ respectively. Values lying beyond the lower and upper adjacent values are considered outliers. The width of each respective plot represents the density plot of the population data.

Figure S6: Type of theory-based teaching, stratified by year group

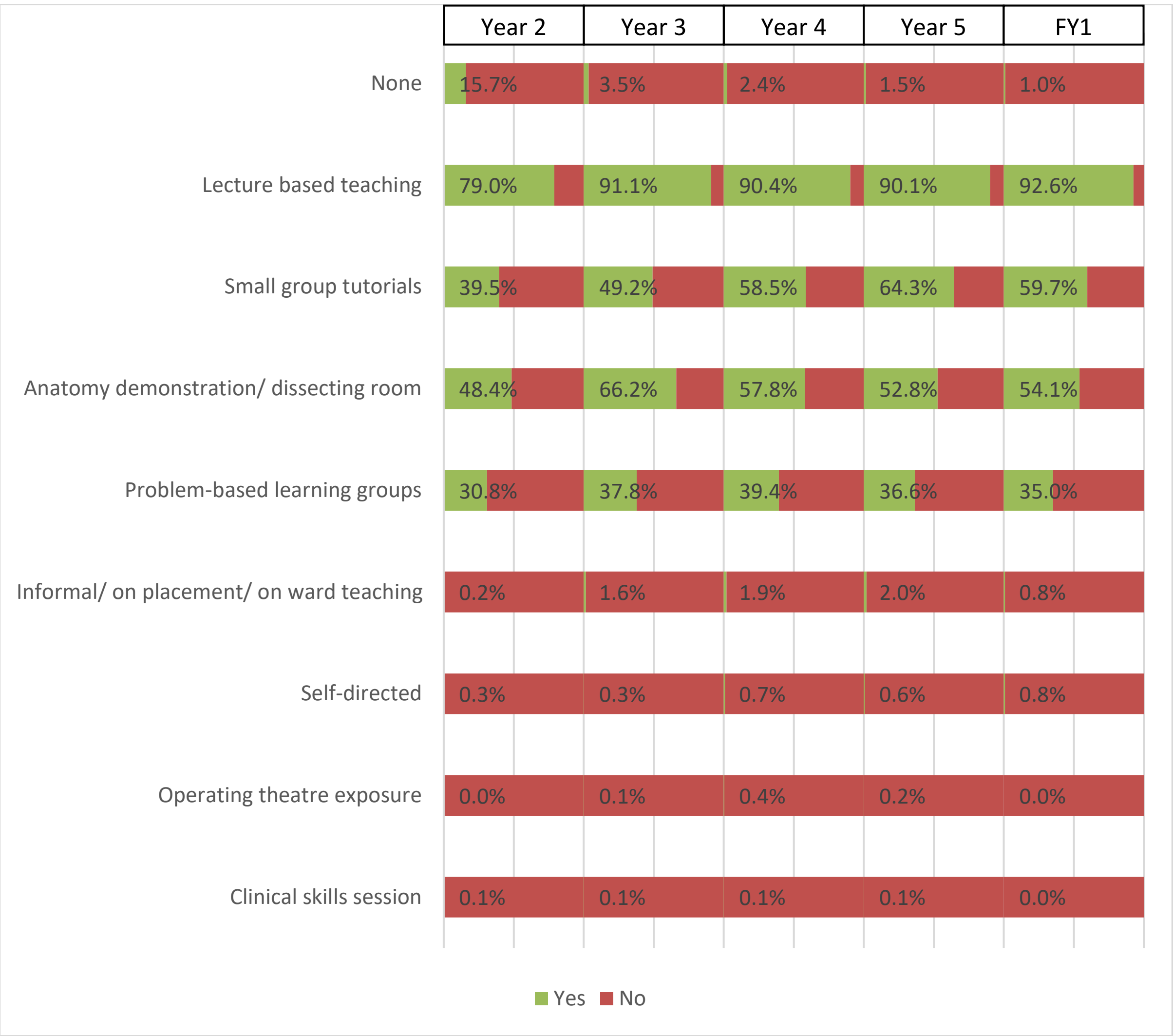

Percentage (%) corresponds to the 'Yes' value.

Figure S7: Cumulative number of theory-based teaching sessions, stratified by year group

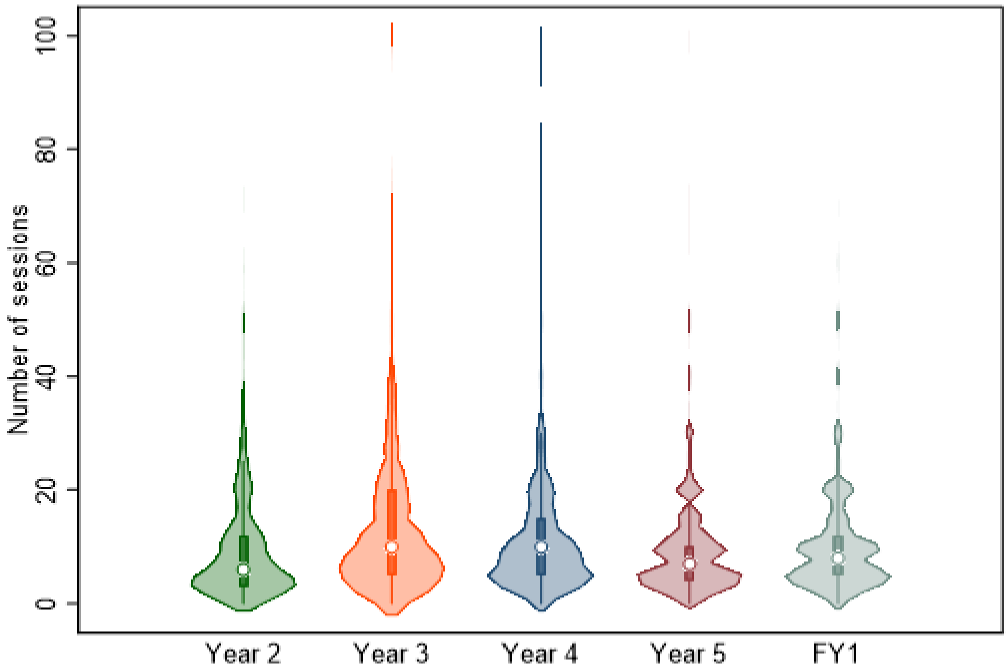

The white dot represents the median value. The coloured bars either side of the median represent the interquartile range (IQR). The vertical lines stretched from the IQR represent the lower and upper adjacent values, defined as $1st\ quartile - 1.5(IQR)$ and $3rd\ quartile + 1.5(IQR)$ respectively. Values lying beyond the lower and upper adjacent values are considered outliers. The width of each respective plot represents the density plot of the population data.

Figure S8: Type of clinical skills teaching, stratified by year group

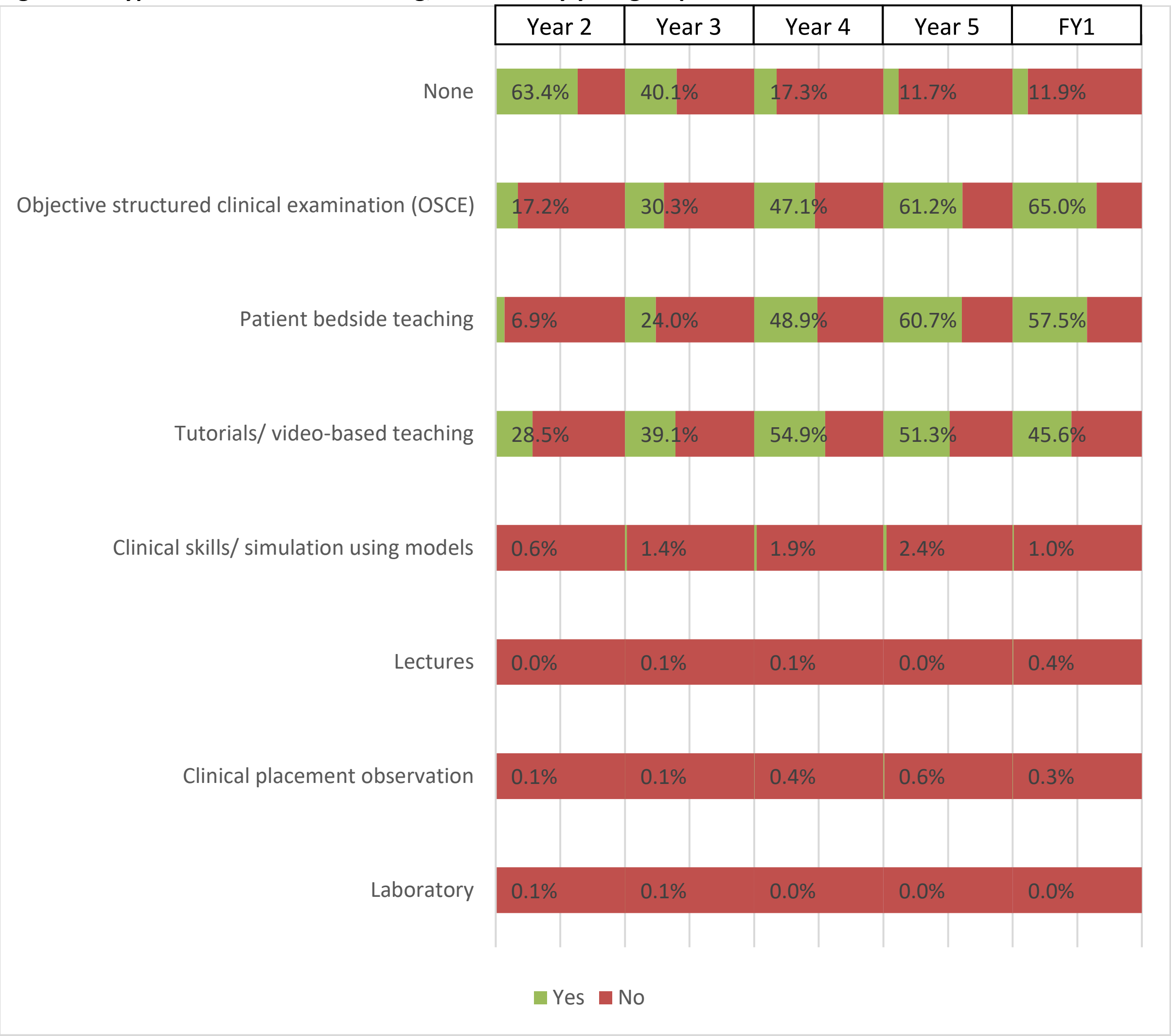

Percentage (%) corresponds to the ‘Yes’ value.

Copyright ©2022 BURST Research Collaborative

Figure S9: Cumulative number of clinical skills sessions, stratified by year group

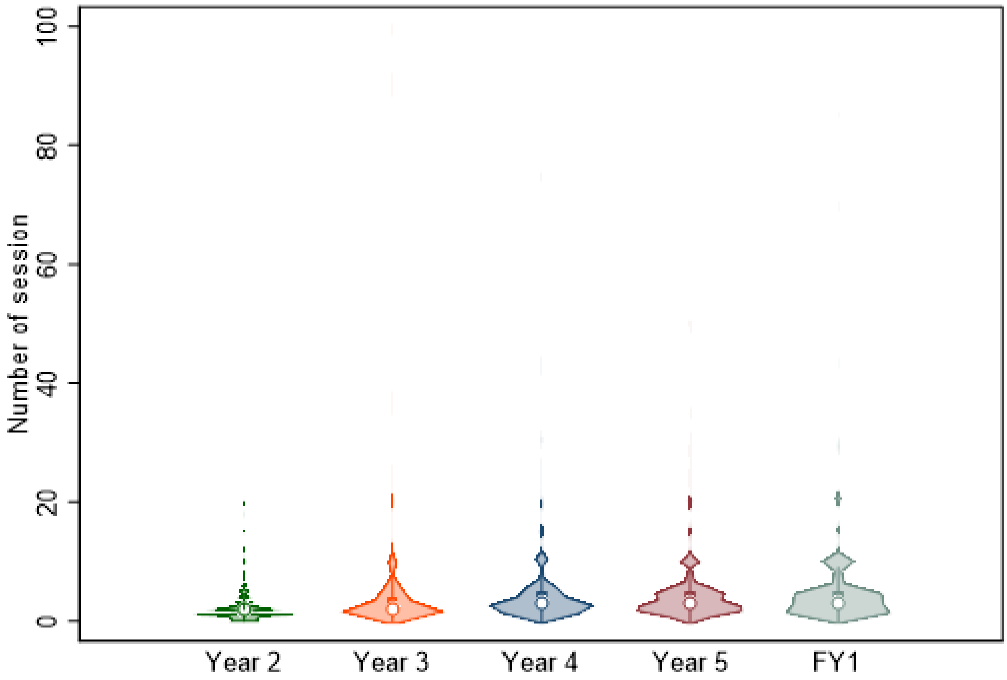

The white dot represents the median value. The coloured bars either side of the median represent the interquartile range (IQR). The vertical lines stretched from the IQR represent the lower and upper adjacent values, defined as $1st\ quartile - 1.5(IQR)$ and $3rd\ quartile + 1.5(IQR)$ respectively. Values lying beyond the lower and upper adjacent values are considered outliers. The width of each respective plot represents the density plot of the population data.

Copyright ©2022 BURST Research Collaborative

Figure S10: Type of clinical attachment, stratified by year group

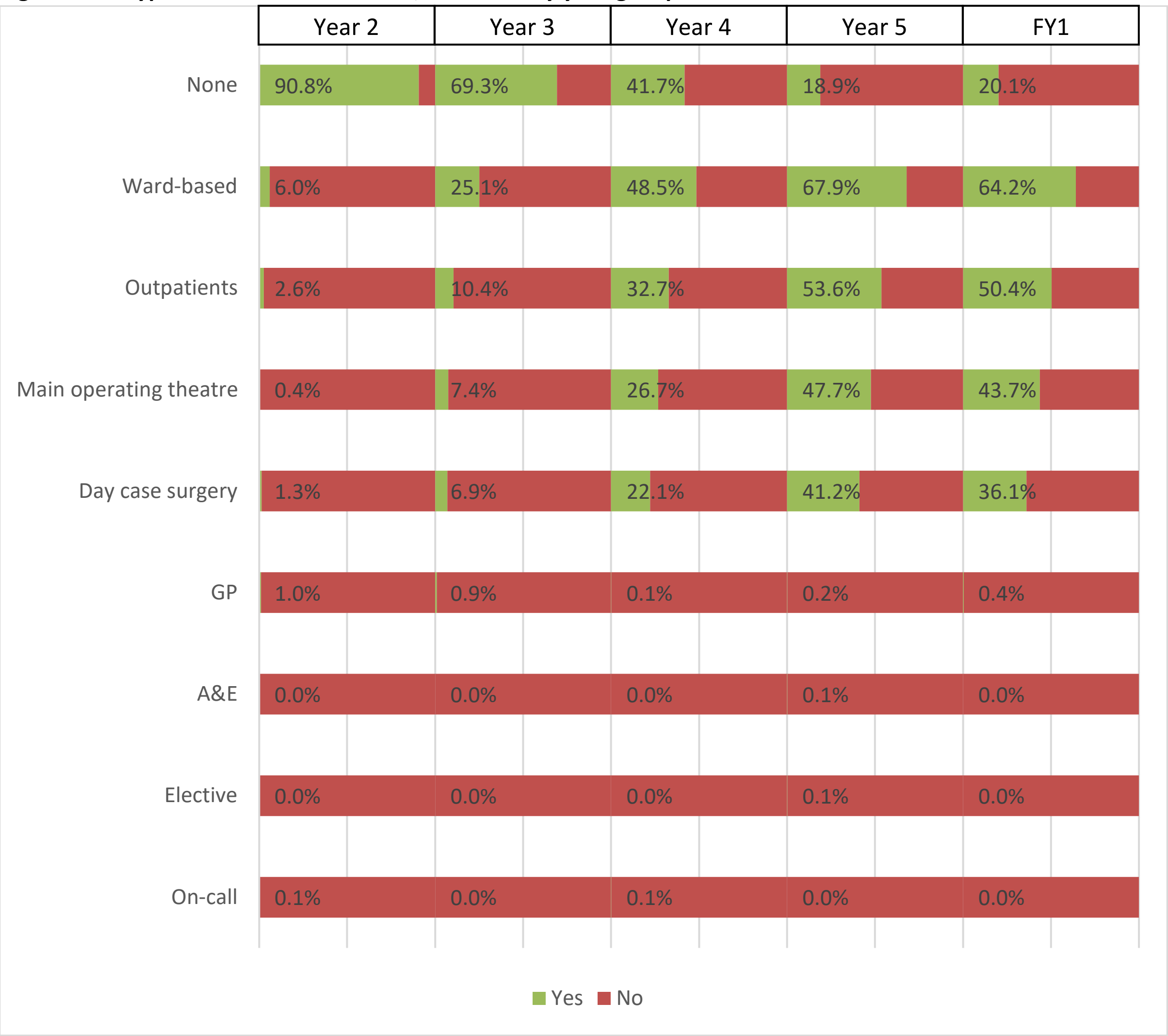

Percentage (%) corresponds to the 'Yes' value.

Copyright ©2022 BURST Research Collaborative

Figure S11: Cumulative number of clinical attachment sessions, stratified by year group

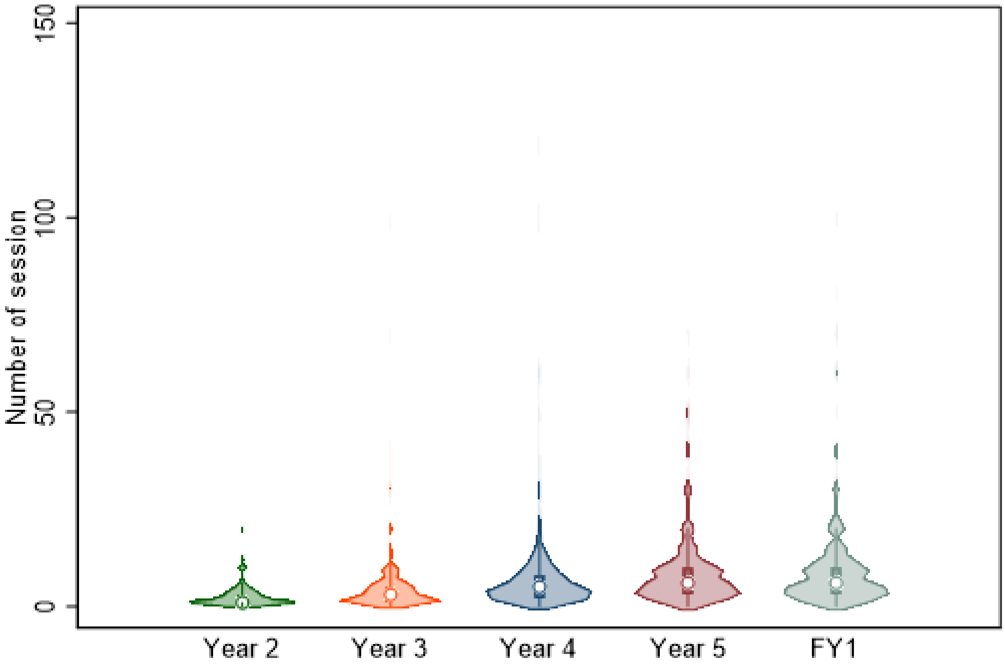

The white dot represents the median value. The coloured bars either side of the median represent the interquartile range (IQR). The vertical lines stretched from the IQR represent the lower and upper adjacent values, defined as $1st\ quartile - 1.5(IQR)$ and $3rd\ quartile + 1.5(IQR)$ respectively. Values lying beyond the lower and upper adjacent values are considered outliers. The width of each respective plot represents the density plot of the population data.

Copyright ©2022 BURST Research Collaborative

Figure S12: Impact of Covid-19 since March 2020 on urology teaching, stratified by year group

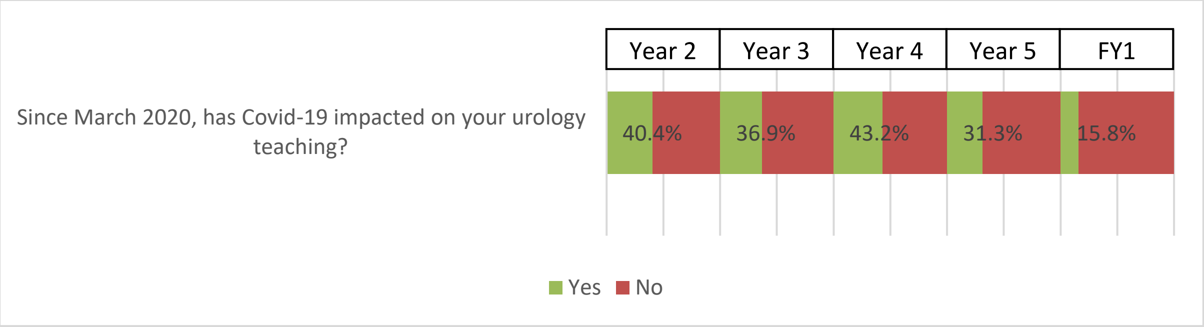

Percentage (%) corresponds to the 'Yes' value.

Figure S13: Of those impacted by Covid-19, the area of impacted urology teaching, stratified by year group

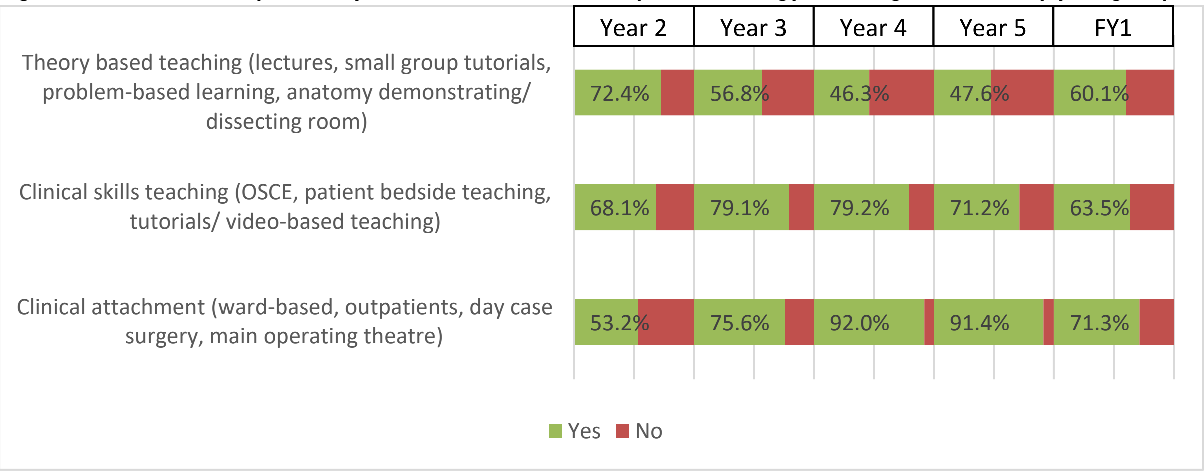

Percentage (%) corresponds to the 'Yes' value.

Copyright ©2022 BURST Research Collaborative

Figure S14: Of those impacted by Covid-19, the reported percentage of original anticipated urology timetable before Covid-19 delivered during the pandemic, stratified by year group

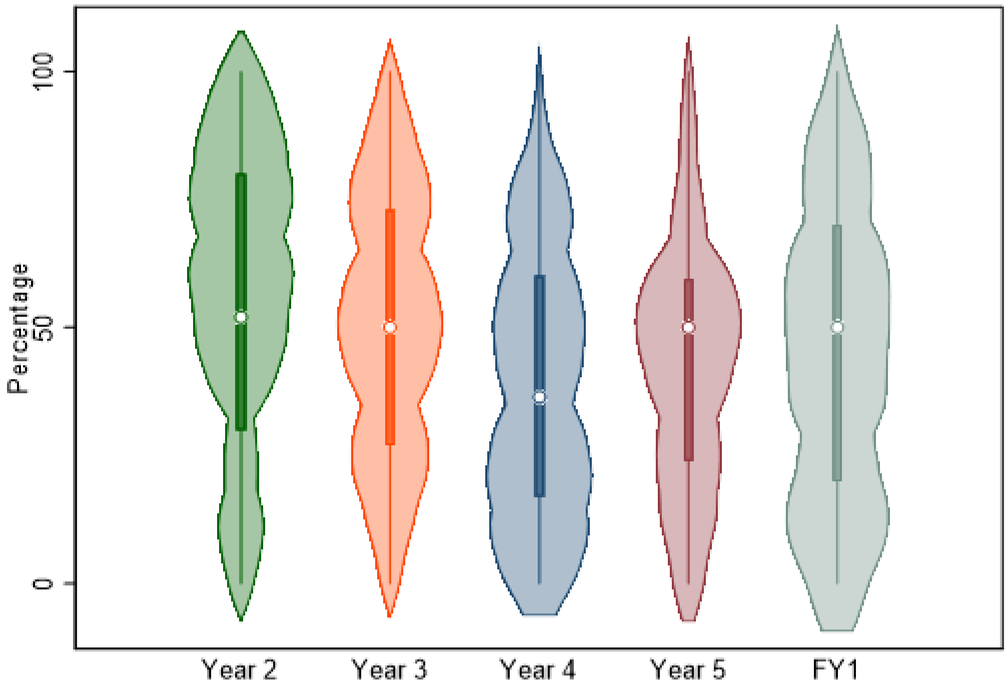

The white dot represents the median value. The coloured bars either side of the median represent the interquartile range (IQR). The vertical lines stretched from the IQR represent the lower and upper adjacent values, defined as $1st\ quartile - 1.5(IQR)$ and $3rd\ quartile + 1.5(IQR)$ respectively. Values lying beyond the lower and upper adjacent values are considered outliers. The width of each respective plot represents the density plot of the population data.

Copyright ©2022 BURST Research Collaborative

Figure S15: Of those impacted by Covid-19, how the impacted urology teaching was provided during the Covid-19 pandemic, stratified by year group

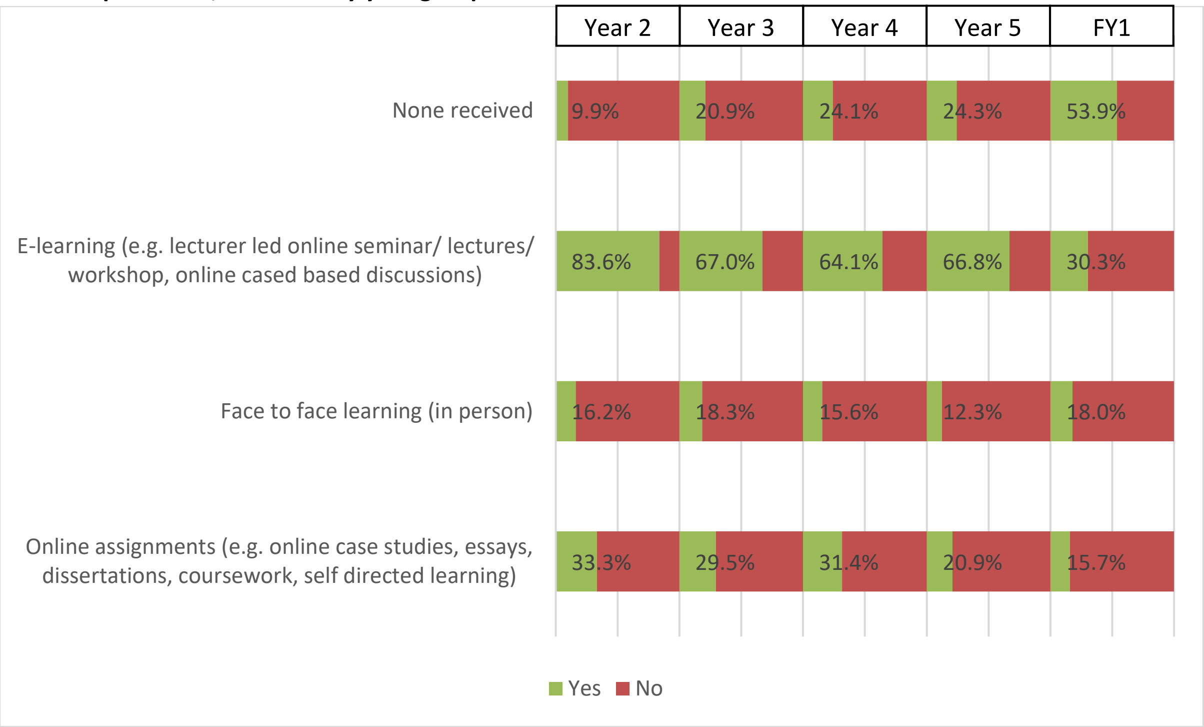

Percentage (%) corresponds to the ‘Yes’ value.

Figure S16: Of those impacted by Covid-19, the reported satisfaction of impacted urology teaching provided during the Covid-19 pandemic by year group

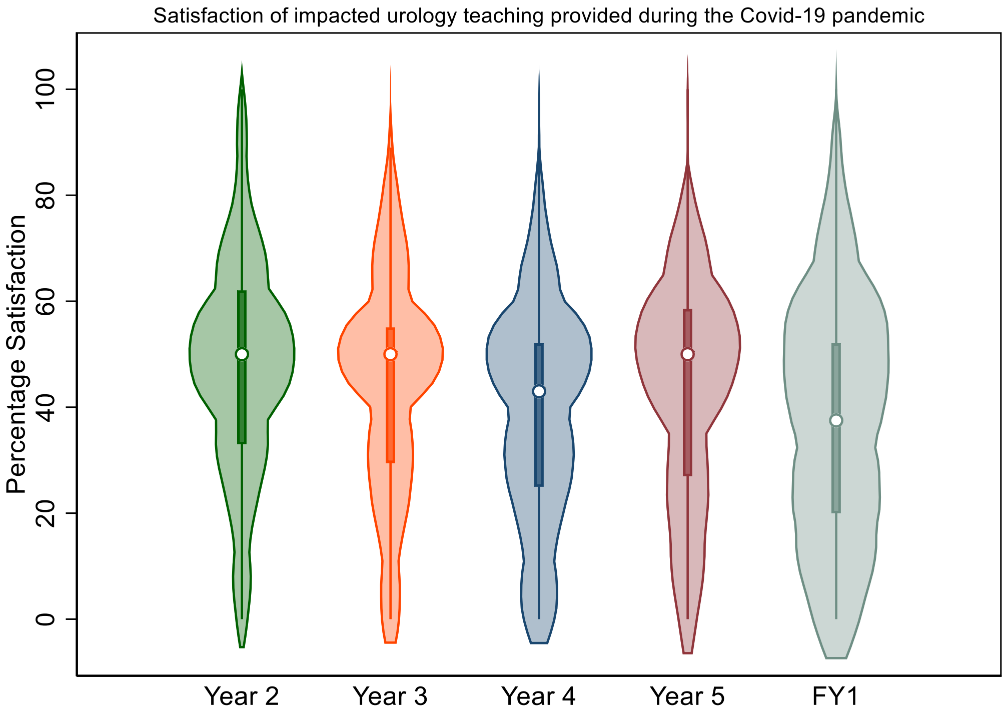

The white dot represents the median value. The coloured bars either side of the median represent the interquartile range (IQR). The vertical lines stretched from the IQR represent the lower and upper adjacent values, defined as $1st\ quartile - 1.5(IQR)$ and $3rd\ quartile + 1.5(IQR)$ respectively. Values lying beyond the lower and upper adjacent values are considered outliers. The width of each respective plot represents the density plot of the population data.

Figure S17: Number of self-selected urology modules (e.g. special study components/ modules) completed by students ($n = 637$) who undertook one during medical school, stratified by year group

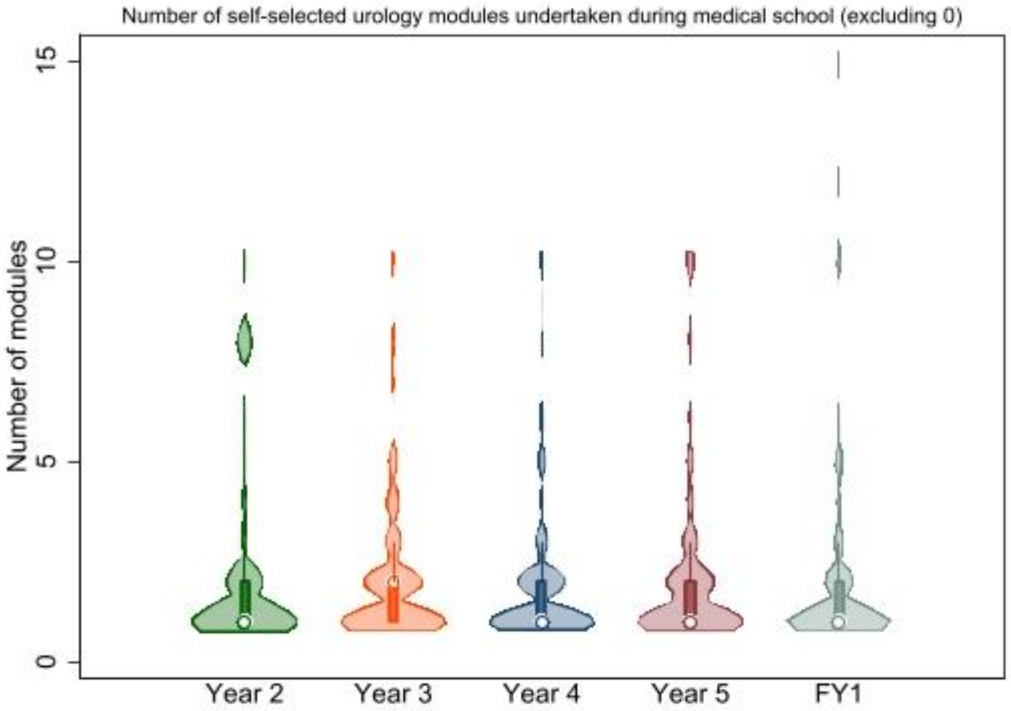

The white dot represents the median value. The coloured bars either side of the median represent the interquartile range (IQR). The vertical lines stretched from the IQR represent the lower and upper adjacent values, defined as $1st\ quartile - 1.5(IQR)$ and $3rd\ quartile + 1.5(IQR)$ respectively. Values lying beyond the lower and upper adjacent values are considered outliers. The width of each respective plot represents the density plot of the population data.

Figure S18: Cross medical school variation in selected procedures performed, key topics taught and key urological procedures observed

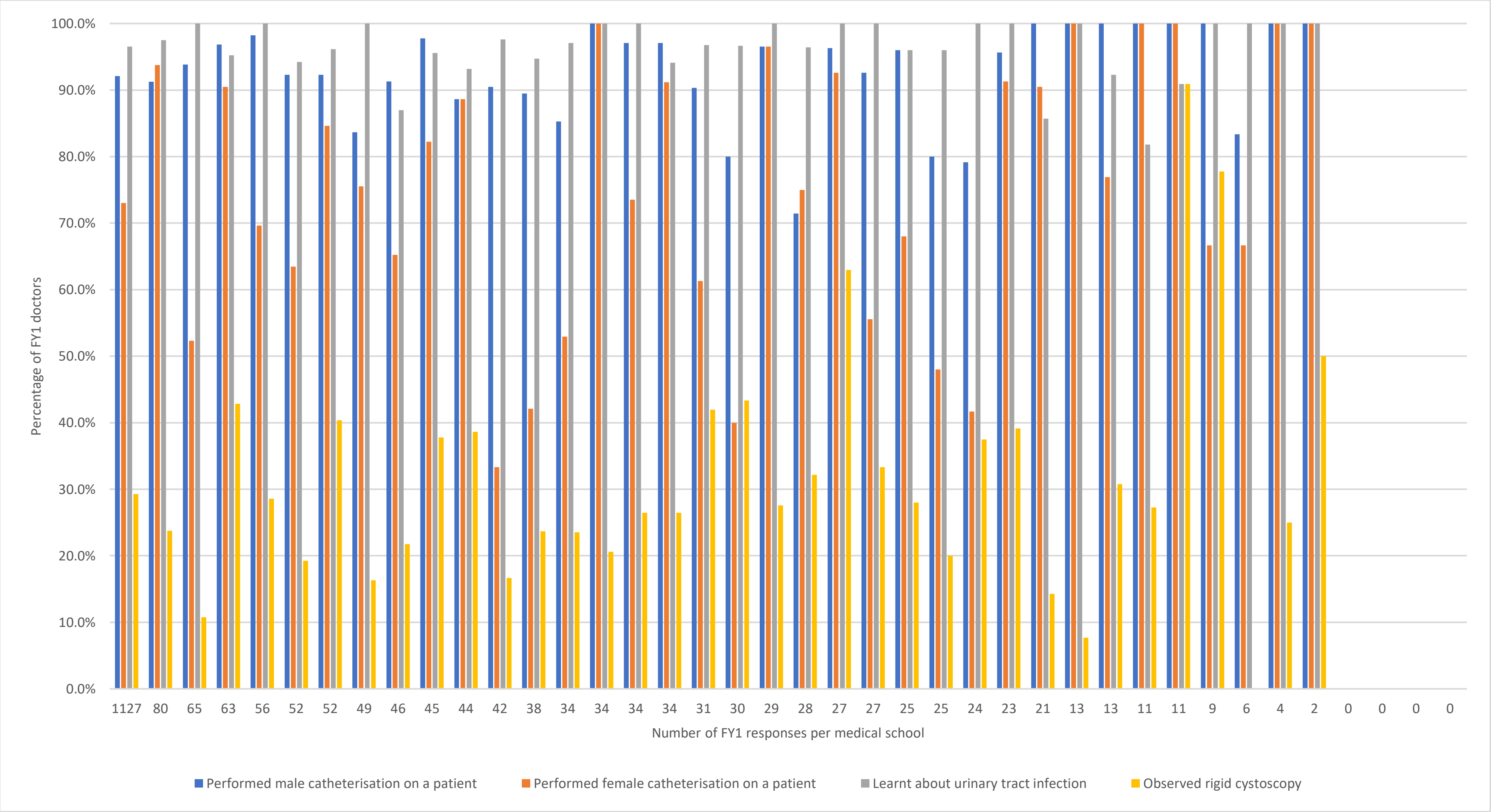

Here are selected topics based on the steering group's impressions on procedures and teaching topics important to the average doctor's urological experience. The 39 medical schools are ordered by number of FY1 doctor responses, with the first columns (n=1,127) representing the overall average. Please note that 4 medical schools are new and therefore do not have a FY1 cohort eligible for the study.

Table S1: Inclusion and exclusion criteria

**Inclusion criteria**	
	• Students between their second and final year enrolled in a medicine course at a UK university which performs under the General Medical Council's and Medical Schools Council's guidelines.
	• Foundation Year 1 doctors that have graduated from a UK university which performs under the General Medical Council's and Medical Schools Council's guidelines.
**Exclusion criteria**	
	• Students in their first year, enrolled in a medicine course at a UK university which performs under the General Medical Council's and Medical Schools Council's guidelines.
	• Foundation Year 2 doctors or more senior.
	• Students at medical schools outside of the UK.

Table S2: Secondary outcomes in the study

Outcome	
	• The number of sessions of theory-based teaching delivered as lecture-based teaching, small group, problem-based teaching and anatomy teaching.
	• The number of sessions of clinical skills teaching delivered as objective structured clinical examination (OSCE), bedside teaching and tutorial-based learning.
	• The number of sessions undertaken during a clinical attachment in urology.
	• The perceived confidence of students in urological examinations and in the management of urological conditions at the competence level expected of a Foundation Year 1 doctor.
	• The perceived confidence of students in performing male and female urethral catheterisation
	• Impact of Covid-19 on urology teaching during the pandemic.
	• The number of extra self-selected urology modules undertaken by medical students.
	• The proportion of medical students who would consider doing a urology placement during their foundation training.
	• The proportion of students who have considered urology as a future career option.

Copyright ©2022 BURST Research Collaborative

Appendix 1: PubMed Indexed Collaborators (BURST Collaborative LEARN Study Group)

LEARN Study Group PubMed Indexed Collaborators*

Ahaan Sanjay Gupta[2], Christopher Khoory[1], Owain Ellis[2], Maiar Elhariry[2], Lucia Harley[2], Viraj Shah[2], Hamza Umar[2], Reeshma Jameel[2], Jade Sangha[2], Miranda Ntorinkansah[1], Natalia Chilal[2], Anna Marshall[2], Balamrit Singh Sokhal[1], Vishal Chandanani[2], Caroline Jarman[2], Aishwarya Sharma[2], Hasti Tarzban[2], Maram Nabahin[2], Lewis Bonsell[3], Benjamin Langhorne[2], Prachi Agarwal[2], Rajwant Kaur[2], Alexander Hunt[2], Cecilia Cirelli[2], Natasha Alford[1], Nesta Baxter[2], Anneesa Malik[2], Abigail Harrison[2], Monty Matson[1], Ronak Shah[2], Cliona Meenan[2], Daanish Ghaffar[2], Katherine Wise[3], Lauren Gurr[2], Tahmeed Ahmed[3], Alice Jones[2], Ankur Singh[2], Chloe Stevens[2], Lien Salcedo[2], Michael Cooke[2], Sunwoo Lee[2], Amanda Godoi[2], Bethany Rose[2], Oluwajenrola Arawole[2], Sanjana Ilangovan[2], Alexander West[3], Alna Dony[2], Anoop Singh Sumal[2], Ariadni Papadopoulou[2], Matan Bone[2], Omar Haque[2], Pooja Patel[2], Rawan Al Dehailan[2], Robert Grogan[2], Brishti Debnath[1], Carina Synn Cuen Pan[2], Isabelle Shaw[2], Katie Tsang[2], Marcus Graham[2], Milad Parsi[2], Nidhi Agarwal[2], Sarah Pengelly[2], Shabnam Tariq[2], Sumbal Bhatti[3], Greta Safoncik[2], Holly Thompson[3], Italia Rosa-Leech[2], Maia Osborne-Grinter[2], Matthew Hennessy[2], Ramisha Basharat[2], Rebecca Paterson[2], Sherie George[2], Shubham Gupta[2], Victoria Porter[2], Ashley Solomon[3], Attika Chaudhary[2], Briony Seden[2], Eimad Basit[2], Grace Kettyle[2], Jesvin Sunny[1], Madhumita Kolluri[2], Precious Jolugbo[2], Syme Bhopal[2], Tzvi Reich[2], Alexander Davies[2], Eleanor Kissane[2], Elena Missir[2], Gareth Hutchinson[2], Jonathan Chua[3], Nicole Wang[2], Olivia Pestrin[2], Ryan Faulder[1], Thomas McLelland[3], Vikash Patel[3], Aimee wilkinson[2], Aliraza Syed[2], Amrit Mann[1], Christina Huon[2], Elina Stokolova[1], Elisa Lau[2], Ella Hobbs[2], George Garratt[2], George Higginbotham[3], Harriet Flashman[2], Hassan Ismahel[2], Ivana Homerova[2], Marcus Boyd[2], Rafi Abdullah[2], Rebecca Lim[2], Rebecca Vitarana[2], Sasha Quarrington[2], Vaishali Kiridaran[2], Ziad Zeidan[2], Alexis Adam[3], Ananya Nair[2], Bernice Johal[2], Brooke Gerrie[2], Christopher Gunn[3], Cora Lowe[2], Dhikshitha Nagaraj[2], Eleanor Deane[1], Jamie McGinn[2], Jennifer Luu[2], Karan Sagoo[1], Katie Sharman[2], Laura Cunningham[3], Megan Scotcher[3], Meshva Amin[3], Natasha Aghtarafi[3], Ruth Goh[2], Shi Pei Loo[2], Tatiana Hamakarim[2], Timothy Ho[2], Xinyu Ye[2], Zain Islam[2], Zoe Zagorac[2], Abdal Zafar[3], Alisha Kanani[2], Armin Nazari [2], Daniel Sescu[1], Inas Alsuhaibani[2], Isabel Munden[3], Jasmine Pattarukuzhyil Jose[2], Mariella Fortune-Ely[3], Michaela Rogers[2], Pratyush Pradeep[2], Stephanos Ghobrial[3], Thomas Hall[3], Yasmin Motarjemi[2], Aaron Campbell[2], Allen Royal[1], Anna Kruczynska[3], Aqeeb Mahmood[2], Ateeq Jamil[2], Boris Wagner[2], Caitlin Murphy[2], Catriona Walker[3], Dilen Parmar[2], Isabelle Mayne[1], Jason Armstrong[2], Laura Inglis[2], Lily Waltham[3], Natalie Ko[2], Rhiannon Kirk[2], Roshni Johnson[2], Ryan Turner[2], Serena Patel[2], Sita Asi[2], Veena Sudarshan[2].

* Collaborators are listed in order of number of survey respondents recruited. Where multiple collaborators have recruited the same number of survey respondents, they are listed in alphabetical order.

[1] Medical School Lead

The roles of a Medical School Lead were to recruit a minimum of 15 medical student survey respondents, to recruit year group leads for each year of their university, and to source the curriculum for each year of their university.

[2] Year Group Lead

The roles of a Year Group Lead were to recruit a minimum of 15 medical student survey respondents, and to liaise with their medical school lead.

[3] Foundation Year 1 Lead

The role of a Foundation Year 1 Lead was to recruit a minimum of 15 Foundation Year 1 survey respondents.

Appendix 2: LEARN Questionnaire

Confidential

LEARN

Please complete the survey below.

Please fill in every question. It should not take longer than 10 minutes.

Thank you!

PLEASE ONLY PARTICIPATE IN THIS SURVEY ONCE

Participation in this study is completely voluntary.

The data collected is completely anonymous. Should you choose to include your name, this will only be used to acknowledge your participation in the study, and to provide you with a certificate, and will not be used for any other purpose.

Access to the data is only granted to the main authors. The data will be used for academic publications, and will be stored in a password-protected account.

LEARN is a national audit, and does not involve access to or collection of private or sensitive data. According to the Health Research Authority (HRA) toolkit, this study does not require an NHS Research Ethics Committee (REC) review.

By clicking "Submit", you give consent for us to use your anonymised data for the purpose of the LEARN study.

If you would like to withdraw your consent at any point, please email learn@bursturology.com. Your data will then be deleted.

Demographics

Please give your full name as you would like it published	______________________________
If you would like a certificate of participation please enter your email address here	______________________________
Please give the name of the lead collaborator who informed you about this survey	______________________________
I confirm that I am studying medicine/have graduated from a UK medical school that is based within the UK, and is not an overseas campus affiliated to a UK medical school.	◯ Yes<br>◯ No

05/04/2021 2:01pm projectredcap.org REDCap

Copyright ©2022 BURST Research Collaborative

Please select your medical school (current medical school if you are a student, or the medical school you graduated from if you are a foundation year 1 doctor)	◯ University of Aberdeen School of Medicine and Dentistry<br>◯ Anglia Ruskin University School of Medicine<br>◯ Aston University Medical School<br>◯ Barts and the London School of Medicine and Dentistry<br>◯ University of Birmingham College of Medical and Dental Sciences<br>◯ Brighton and Sussex Medical School<br>◯ University of Bristol Medical School<br>◯ University of Buckingham Medical School<br>◯ University of Cambridge School of Clinical Medicine<br>◯ Cardiff University School of Medicine<br>◯ University of Dundee School of Medicine<br>◯ Edge Hill University Medical School<br>◯ The University of Edinburgh Medical School<br>◯ University of Exeter Medical School<br>◯ University of Glasgow School of Medicine<br>◯ Hull York Medical School<br>◯ Imperial College London Faculty of Medicine<br>◯ Keele University School of Medicine<br>◯ Kent and Medway Medical School<br>◯ King's College London GKT School of Medical Education<br>◯ Lancaster University Medical School<br>◯ University of Leeds School of Medicine<br>◯ University of Leicester Medical School<br>◯ University of Liverpool School of Medicine<br>◯ London School of Hygiene & Tropical Medicine<br>◯ University of Manchester Medical School<br>◯ Newcastle University School of Medical Education<br>◯ Norwich Medical School<br>◯ University of Nottingham School of Medicine<br>◯ University of Nottingham - Lincoln Medical School<br>◯ University of Oxford Medical Sciences Division<br>◯ Plymouth University Peninsula Schools of Medicine and Dentistry<br>◯ Queen's University Belfast School of Medicine<br>◯ University of Sheffield Medical School<br>◯ University of Southampton School of Medicine<br>◯ University of St Andrews School of Medicine<br>◯ St Georges University of London<br>◯ University of Sunderland School of Medicine<br>◯ Swansea University Medical School<br>◯ University of Central Lancashire School of Medicine<br>◯ University College London Medical School<br>◯ University of Warwick Medical School<br>◯ Other
Other	________________________________
Are you an undergraduate student, graduate student (4 year course) or Foundation Year 1 doctor?	◯ Undergraduate (5-6 year course)<br>◯ Graduate (4 year course)<br>◯ Foundation year 1 doctor
Are you currently in an intercalated year?	◯ Yes<br>◯ No

Copyright ©2022 BURST Research Collaborative

What year of your medical degree are you currently in?

(if you are currently in an intercalated year please select the clinical year you would have progressed to. For example if you are intercalating after 3rd year select 4th year on the survey)

◯ 1st year
◯ 2nd year
◯ 3rd year
◯ 4th year
◯ 5th (final) year

What year of your medical degree are you currently in?

(if you are currently in an intercalated year please select the clinical year you would have progressed to. For example if you are intercalating after 3rd year select 4th year on the survey)

◯ 1st year
◯ 2nd year
◯ 3rd year
◯ 4th (final) year

YOUR MEDICAL SCHOOL'S CURRICULUM

Please click on the attachment to view your curriculum as an aide memoire for this study.

[Attachment: "UoA LEARN Instructions.pdf"]

Please click on the link to view your curriculum as an aide memoire for this study

[Attachment: "Anglia Ruskin Medical School.docx"]

Please access your medical school curriculum as an aide memoire for this study

1. Login to Blackboard with your student details (https://vle.aston.ac.uk/webapps/login/)
2.Go to courses and select the MS1000 (Fundamentals of Medicine) folder
3.Select 'Course Content', then select either 'Teaching Period 1' or the 'Teaching Period 2' folder
4.In the TP1/TP2 folder, blocks of learning material are further divided into folders, please select the respective block folder, then click into 'block sessions' to access the block curriculum.
5. Alternatively for the block overview, select 'Core Content Overview' after clicking into the block folders, then select 'Summary or Core Content

Please access your medical school curriculum as an aide memoire to this study

1st year: https://compas.smd.qmul.ac.uk/outcomes/module/id/42#291
2nd year: https://compas.smd.qmul.ac.uk/outcomes/module/id/43
3rd year:
https://2019.qmplus.qmul.ac.uk/pluginfile.php/594962/mod_resource/content/7/Met%203B%20Handbook%202019-20.pdf
4th year: NIL
5th year: https://qmplus.qmul.ac.uk/mod/page/view.php?id=443740 and
https://qmplus.qmul.ac.uk/mod/page/view.php?id=435518

Please refer to your medical school curriculum as an aide memoire to this study

Year 1
> MBChB Year 1 (Canvas page)
> Foundations 1 Overview AND Foundations 2 Overview
> Learning Outcomes
Year 2
> MBChB Year 2 (Canvas page)
> Foundations 3 Overview
> Module Learning Outcomes OR Renal and Urinary System (REN) > Learning Outcomes / Handbook
Year 3
> MBChB Year 3 (Canvas page)
> Clinical Core 2 handbook
Year 4
> MBChB Year 4 (Canvas page)
> Specialty Medicine AND Surgery & Peri-Operative Care
> SPM Handbook AND SPC Handbook
Year 5
> MBChB Year 5 (Canvas page)

Copyright ©2022 BURST Research Collaborative

> AIP
> Handbook
GEC
> MBChB GEC
> Fuels AND Loss and Renewal
> Module Handbook

Please click on the attachment to view the curriculum outline as an aide memoire for this study.

[Attachment: "BM BS Undergraduate Curriculum August 2016.pdf"]

Please copy and paste the link to access your medical school curriculum as an aide memoire to this study

https://drive.google.com/drive/folders/1wN7WShEXlngOii03dPRk1gy4XGsHmL3u?usp=sharing

---

Please copy and paste the link to access your medical school curriculum as an aide memoire to this study

https://drive.google.com/drive/folders/1NT9ATSqxpUOlnXBwNjtZOldnsiwEJ5gX?usp=sharing

---

Please copy and paste the link to access your medical school curriculum as an aide memoire to this study

https://drive.google.com/drive/folders/1DddgSWI7jA7Ay49Ys2FwMK6axLP2znhO?usp=sharing

---

Please copy and paste the link to access your medical school curriculum as an aide memoire to this study

https://drive.google.com/drive/folders/10R5CUzYHfyhtjGjDeglzDV5HGsOuXLfJ?usp=sharing

---

Please access your medical school curriculum as an aide memoire to this study

Year 2
https://medblogs.dundee.ac.uk/renal-sip/teaching-materials/renal-block-overview/
Year 4/ Year 3 GEM
https://medblogs.dundee.ac.uk/urology-pip/block-information/ninewells-information/
Year 5
Acute care
https://medblogs.dundee.ac.uk/acute-care-pip/learning-outcomes/
Medicine and surgery
https://medblogs.dundee.ac.uk/5th-year-programmes-pip/teaching-programmes/foundation-assistantships/fa-medicine-surgery/

Please access your medical school curriculum as an aide memoire for this study

(Urology only taught in Y2 and Y5)

Please go to learn.ed.ac.uk to view the curriculum.

Follow links to either year 2 or year 5 renal/urology modules to view the core content.
Year 5 page link:
https://www.learn.ed.ac.uk/webapps/blackboard/content/listContent.jsp?course_id=_82763_1&content_id=_5015277_1

Please open the attachment to remind yourself of the urology curriculum in your medical school

[Attachment: "University of Exeter Urology Curriculm .docx"]

Please open this attachment with the links to your medical school's curriculum as an aide memoire to this study.

[Attachment: "Glasgow Cirriculum Links.docx"]

Please open the attachment as an aide memoire to your medical school's curriculum for this study

[Attachment: "MBBS Curriculum Map 2019-20.pdf"]

Please open the attachment as an aide memoire of your curriculum for this study.

[Attachment: "urology curriculum imperial.docx"]

Please copy and paste the link to access your medical school curriculum as an aide memoire for this study

https://drive.google.com/drive/folders/1w7X4TEH0ovn1Ld_eWY6KavkcjwJfGceO?usp=sharing

---

Please copy and paste the link for access to your medical school curriculum as an aide memoire for this study

https://drive.google.com/drive/folders/1KFuevJFFi3z0153e5YpTgM3hhVhC6kON?usp=sharing

---

Please see instructions below to access your medical school curriculum as an aide memoire for this study

Log into Student Portal
Click on Student Record
Clink on Course Module Enrolment
Curriculum can be also accessed on the following link:
https://portal.lancaster.ac.uk/portal/mpc/programmes/016838/000119

Please copy and paste the link to access your medical school curriculum as an aide memoire for this study

https://drive.google.com/drive/folders/13MPy8iN1oSd8lg6EGaZBZ4o9NgKkOC7Z?usp=sharing

---

Please open the attachment as an aide memoire of your curriculum for this study.

[Attachment: "Curriculum.pdf"]

Please open the attachment as an aide memoire of your curriculum for this study.

[Attachment: "Urology Objectives.docx"]

Please copy and paste the link to access your medical school curriculum as an aide memoire for this study

https://drive.google.com/drive/folders/16epGzpxgq-EDtzKsT_xuRr3Yn-ltKxt5?usp=sharing

---

Please follow the instructions and links to access your medical school curriculum as an aide memoire for this study

Curriculum Mapping Tool (CMT) for MB ChB Medicine at The University of Manchester (Intranet)
https://cmt.mhs.man.ac.uk/ (All years, Years 1 -5)
Overview of the MB ChB Curriculum on the Official University of Manchester Website
https://www.manchester.ac.uk/study/undergraduate/courses/2021/01428/mbchb-medicine/course-details/#course-profile

Copyright ©2022 BURST Research Collaborative

Please follow instructions below to access your medical school curriculum as an aide memoire for this study

Log onto the MLE at https://mle.ncl.ac.uk/
Click the road icon in the top left corner to access content from your previous year
Either look at relevant cases from the year or use the curriculum search tool to look for relevant learning outcomes or conditions.

Please copy and paste the link to access your medical school curriculum as an aide memoire for this study

https://drive.google.com/drive/folders/1EmGA0sDAcaYIDpZOHkOPVH6XtXuuLJne?usp=sharing

---

Please copy and paste the link for access to your medical school curriculum as an aide memoire for this study

https://drive.google.com/drive/folders/1eAqLg49LajQVpatx9rr4IqoxhKZiCmNq?usp=sharing

---

Please copy and paste the link for access to your medical school curriculum as an aide memoire for this study

https://drive.google.com/drive/folders/1Nn5q9bKq35xZRuOA88eLb9e0fLAQ25Iq?usp=sharing

---

Please use the attachment for access to your medical school curriculum as an aide memoire for this study

[Attachment: "Integrated curriculum in medicine and surgery 22019-20.pdf"]

Please open the attachment as an aide memoire of your curriculum for this study.

[Attachment: "BURST - Curriculum.xlsx"]

Please copy and paste the link for access to your medical school curriculum as an aide memoire for this study

https://drive.google.com/drive/folders/19ffmnusyjN7zMA36lPo1GJIN_NPT3vv_?usp=sharing

---

Please copy and paste the link for access to your medical school curriculum as an aide memoire for this study

https://drive.google.com/drive/folders/1Eq5Ua8b5m7GlCPE0eFQFWPArXarVXw2h?usp=sharing

---

Please copy and paste the link for access to your medical school's Curriculum as an aide memoire for this study

Undergraduate Medicine curriculum:
https://www.st-andrews.ac.uk/subjects/medicine/medicine-bsc-a100/#76830

Scottish Graduate Entry Medicine curriculum:
https://www.st-andrews.ac.uk/subjects/medicine/scotgem-mbchb/#77037

Please copy and paste this link to access your medical school's curriculum as an aide memoire for this study

https://drive.google.com/drive/folders/1FokCS-p7Idn99FnIMEejdehD_R5gaIls?usp=sharing

---

Please copy and paste the link for access to your medical school's curriculum as an aide memoire for this study

https://drive.google.com/drive/folders/1I3NTu__P6NVUo4D8C6PxP9PZM2K2oXOX?usp=sharing

Copyright ©2022 BURST Research Collaborative

Please copy and paste the link for your medical school's curriculum map as an aide memoire for this study

Curriculum map
https://www.ucl.ac.uk/medical-school/current-mbbs-students/curriculum-map

Please copy and paste the link to access your medical school's curriculum as an aide memoire for this study

https://drive.google.com/drive/folders/1XZb4RboENnwZcJE359vOFUEgpCqx9VDS?usp=sharing

Urology teaching - topics

Please select all topics covered in your urology teaching during medical school so far

- ☐ Acute renal tract stone disease
- ☐ Acute (or chronic) abdominal pain referable to the urinary tract
- ☐ Lower urinary tract symptoms (LUTS) in male patients
- ☐ Lower urinary tract symptoms (LUTS) in female patients
- ☐ Haematuria
- ☐ Urinary tract infection (UTI)
- ☐ Scrotal swelling and pain
- ☐ Urinary incontinence in male patients
- ☐ Urinary incontinence in female patients
- ☐ Urological cancer (including kidney, bladder, prostate, penis and testis)
- ☐ Male infertility
- ☐ Erectile dysfunction
- ☐ Paediatric urology conditions (e.g. UTI, undescended testis, phimosis and acute scrotal pain/swelling)
- ☐ Acute kidney injury
- ☐ None

Type of urology teaching method - theory based teaching

What type of theory based teaching in urology have you had so far in medical school?

(select all that apply)

- ☐ None
- ☐ Lecture based teaching
- ☐ Small group tutorials
- ☐ Problem-based learning groups
- ☐ Anatomy demonstration/Dissecting room
- ☐ Other

Please explain 'other'

____

What is the total number of sessions that have been dedicated to theory based teaching in urology?

(an example of a session is a morning or afternoon lecture, a small group tutorial, a problem based learning group session, or an anatomy demonstration)

____
(Please insert a whole number)

Copyright ©2022 BURST Research Collaborative

Type of urology teaching method - clinical skills teaching

What type of clinical skills teaching in urology have you had so far in medical school?

(select all that apply)

☐ None
☐ Objective structured clinical examination (OSCE)
☐ Patient bedside teaching
☐ Tutorials/video-based teaching
☐ Other

Please explain 'other'

____

What is the total number of sessions that have been dedicated to clinical skills teaching in urology?

(an example of a session is a morning or afternoon OSCE session, being supervised practising a clinical skills examination on a patient, or a tutorial/video)

____
(Please insert a whole number)

Type of urology teaching method - clinical attachment

What type of clinical attachments in urology have you had so far in medical school?

(select all that apply)

☐ None
☐ Ward-based
☐ Outpatients
☐ Day case surgery
☐ Main operating theatre
☐ Other

Please explain 'other'

____

What is the total number of sessions that have been dedicated to clinical attachements in urology?

(an example of a session is a morning or afternoon outpatient clinic, a teaching ward round, a urology operating list session, or a morning/afternoon/day attached to a urology team)

____
(Please insert a whole number)

Urological Procedures

Have you ever observed a male genital examination

◯ Yes
◯ No

Have you ever performed a male genital examination?

◯ Never
◯ Only on a model
◯ Only on a patient
◯ On both a model and a patient

Have you ever observed digital rectal examination?

◯ Yes
◯ No

Have you ever performed a digital rectal examination?

◯ Never
◯ Only on a model
◯ Only on a patient
◯ On both a model and a patient

Copyright ©2022 BURST Research Collaborative

Have you ever observed male catheterisation?	◯ Yes<br>◯ No
Have you ever performed male catheterisation?	◯ Never<br>◯ Only on a model<br>◯ Only on a patient<br>◯ On both a model and a patient
How many have you performed?	__________________________________
Have you ever observed female catheterisation?	◯ Yes<br>◯ No
Have you ever performed female catheterisation?	◯ Never<br>◯ Only on a model<br>◯ Only on a patient<br>◯ On both a model and a patient
How many have you performed?	__________________________________
Have you ever observed a suprapubic catheter insertion or change?	◯ Yes<br>◯ No
Have you ever observed flexible cystoscopy?	◯ Yes<br>◯ No
Have you ever observed a rigid cystoscopy?	◯ Yes<br>◯ No
Have you ever observed a transurethral resection of prostate (TURP)?	◯ Yes<br>◯ No
Have you ever observed a transurethral resection of bladder tumour (TURBT)?	◯ Yes<br>◯ No
Have you ever observed a CT urogram?	◯ Yes<br>◯ No
Have you ever interpreted and discussed a CT urogram with supervision?	◯ Yes<br>◯ No
Have you ever interpreted and discussed an intravenous urogram (IVU) with supervision?	◯ Yes<br>◯ No
Have you ever observed a transrectal ultrasound (TRUS) +/- prostate biopsy?	◯ Yes<br>◯ No
Have you ever observed urodynamics?	◯ Yes<br>◯ No
Have you ever interpreted a voiding flow rate with supervision?	◯ Yes<br>◯ No

Copyright ©2022 BURST Research Collaborative

Have you ever observed any of the following operations:
(select all that apply)

- ☐ Ureteroscopy
- ☐ Laparotomy
- ☐ Circumcision
- ☐ Scrotal surgery
- ☐ None of the above

Confidence in practising urology in clinical areas

As a foundation year 1 doctor, how confident are you in clerking (history and examination) a urological patient?

Not confident — Average — Very confident

(Place a mark on the scale above)

How confident are you in clerking a urological patient (taking a history and performing an examination), as would be expected of a foundation year 1 doctor?

Not confident — Average — Very confident

(Place a mark on the scale above)

As a foundation year 1 doctor, how confident are you in initiating management for a common urological condition?

Not confident — Average — Very confident

(Place a mark on the scale above)

How confident are you in initiating management for a common urological condition, as would be expected of a foundation year 1 doctor?

Not confident — Average — Very confident

(Place a mark on the scale above)

As a foundation year 1 doctor how confident are you in inserting a male catheter?

Not confident — Average — Very confident

(Place a mark on the scale above)

How confident are you in inserting a male catheter as would be expected of a foundation year 1 doctor?

Not confident — Average — Very confident

(Place a mark on the scale above)

As a foundation year 1 doctor how confident are you in inserting a female catheter?

Not confident — Average — Very confident

(Place a mark on the scale above)

How confident are you in inserting a female catheter as would be expected of a foundation year 1 doctor?

Not confident — Average — Very confident

(Place a mark on the scale above)

Copyright ©2022 BURST Research Collaborative

Impact of COVID-19 on urology teaching

Since March 2019, has COVID-19 impacted on your urology teaching?	◯ Yes<br>◯ No
In which area(s) has your urology teaching been impacted?<br>(select all that apply)	☐ Theory based teaching (lectures, small group tutorials, problem-based learning, anatomy demonstrating/dissecting room)<br>☐ Clinical skills teaching (OSCE, patient bedside teaching, tutorials/video-based teaching)<br>☐ Clinical attachment (ward-based, outpatients, day case surgery, main operating theatre)
What percentage of your original anticipated urology timetable before COVID did you end up receiving during the pandemic period?	0% 50% 100%<br>*(Place a mark on the scale above)*
How was the impacted urology teaching provided during the COVID-19 pandemic?<br>(select all that apply)	☐ None received<br>☐ Face to face learning (in person)<br>☐ E-learning (eg lecturer led online seminar/lectures/workshop, online case based discussions)<br>☐ Online assignments (eg online case studies, essays, dissertations, coursework, self directed learning)
How satisfied were you with the urology teaching you received during the Covid-19 pandemic?	Very dissatisfied Neutral Very satisfied<br>*(Place a mark on the scale above)*

Extra self-selected urology modules

How many, if any, extra self-selected urology modules (e.g. special study components/modules) have you undertaken so far in medical school?	____________________<br>(please insert whole number)

Postgraduate career

Would you want a urology rotation in your foundation doctor training years (FY1 and FY2)	◯ Yes ◯ No
Do you have a urology rotation in your foundation year 1?	◯ Yes<br>◯ No
Would you want a urology rotation in your foundation year 2?	◯ Yes ◯ No
Have you, or are you considering a career in urology	◯ Yes ◯ No

Copyright ©2022 BURST Research Collaborative

Concluding questions

Do you feel there is sufficient teaching in your medical course on urology?	◯ Yes ◯ No
Do you feel that you have had enough career exposure/information on the pathways to a career in urology?	◯ Yes ◯ No

Copyright ©2022 BURST Research Collaborative

Appendix 3: Checklist for Reporting Results of Internet E-Surveys (CHERRIES)

Item category	Checklist item	Explanation
Design		
	Describe survey design	The target population was UK medical students and newly qualified Foundation Year 1 doctors. A variety of sample techniques were utilised. The first was probability-based by using list-based sampling via emailing surgical societies. This was combined with non-probability sampling (convenience, voluntary response and snowball sampling).
IRB (Institutional Review Board) approval and informed consent process		
	IRB approval	As per UK NHS Health Research Authority guidance, NHS Research Ethics Committees review exemption applied.
	Informed consent	Informed consent was obtained in the beginning by attaching a cover note describing the survey, the approximate time to complete, who had access to the data, and the purpose of the study.
	Data protection	The responses were stored in a password protected computer. The secure REDCap database is hosted at University College London and was only accessible by the LEARN Steering Committee who had access to the results through their password protected accounts.
Development and pre-testing		
	Development and testing	The REDCap survey was piloted prior to final release. The scope, choice of questions and format was drafted by AN and SK and edited by other authors as part of the BURST Research Collaborative peer review process involved internal peer review within the collaborative, and external peer review by invited experts in the field.
Recruitment process and description of the sample having access to the questionnaire		
	Open survey versus closed survey	This was an open survey.
	Contact mode	Initial contact with the potential participants was made on the internet.
	Advertising the survey	The survey was advertised on the BURST website, Twitter, Surgical Society pages and through newsletters.
Survey administration		
	Web/E-mail	This was an e-survey, with data captured by the REDCap platform. The survey link was posted on social media, the BURST website and surgical society pages and newsletters.
	Context	The BURST website is an online webpage detailing the activities of the BRUST group and would be visited by urologists or researchers interested in the activities of BURST.<br>Surgical society pages and newsletters would be engaging with students interested in surgery and research.
	Mandatory/voluntary	The survey was voluntary and completed by individuals who were interested in taking part.
	Incentives	There were no monetary incentives to complete the survey. All survey participants would have their name listed in the appendix should they wish to include their name.
	Time/date	Data was collected between 3rd October to 20th December 2020, approximately an 11-week period.

	Randomisation of items or questionnaires	Items were not randomised or alternated.
	Adaptive questioning	Adaptive questioning was applied using the branching logic function in REDCap.
	Number of items	The survey contained 102 items.
	Number of screens (pages)	The survey contained one page.
	Completeness check	The survey was constructed to have a mandatory response to all items. Selection of one item was enforced.
	Review step	Respondents were able to review their answers prior to submitting the survey.
Response rates		
	Unique site visitor	A unique visitor could be identified through their demographics (medical school, stage of training) and through identifier features (such as name, email address).
	View rate (ratio unique site visitors/unique survey visitors)	We are unable to identify this due to the varied ways of advertising through social media and newsletters.
	Participation rate (ratio unique survey page visitors/agreed to participate)	We are unable to identify this due to the varied ways of advertising through social media and newsletters.
	Completion rate (ratio agreed to participate/finished survey)	Out of 8,346 responses received, 7844 were completed. This gives a completion rate of 94.0%.
Preventing multiple entries from the same individual		
	Cookies used	Cookies were not used.
	IP check	IP address was not used.
	Log file analysis	Log file analysis was not used.
	Registration	This was an open survey, and therefore registration was not utilised.
Analysis		
	Handling of incomplete questionnaires	Incomplete questionnaires were excluded and not analysed.
	Questionnaires submitted with an atypical timestamp	An atypical time stamp was not used.
	Statistical correction	Propensity scores or weighting of items was not used.

Appendix 4: LEARN Protocol

BURST
British Urology Researchers in Surgical Training

LEARN

Acronym: LEARN

Long title: LEARN: uro**L**ogical t**EA**ching in b**R**itish medical schools **N**ationally - a national retrospective multi-centre audit of urology teaching across British medical schools

Protocol Version 1.1 23rd December 2020

Copyright ©2022 BURST Research Collaborative

Contents

1. Project contributors 3

2. Project timeline 4

3. Background 5

3.1. Introduction 5

3.2. The BURST Research Collaborative 5

3.3. Rationale 6

4. Funding 7

5. Study design 8

6. General methods 8

7. Study population 9

7.1. Inclusion criteria 9

7.2. Exclusion criteria 9

8. Objectives 10

8.1. Primary objective 10

8.2. Secondary objectives 10

9. Outcomes 11

9.1. Primary outcome 11

9.2. Secondary outcomes 11

10. Data 12

10.1. Registration 12

10.2. Data quality 12

10.3. Data management 12

11. Authorship 13

12. Dissemination 13

13. Ethics 13

14. Acknowledgements 13

Appendix A: UK medical schools 14

References 16

Copyright ©2022 BURST Research Collaborative

1. Project contributors

* Members are listed alphabetically by surname.

Name	Training stage	Twitter	Contribution
Aqua Asif	4th Year, Leicester Medical School	@AquaOishee	Study design<br>Advertising
Nikita Bhatt	Urology SpR, East of England	@NikitaB0709	Protocol revisions
William Cambridge	5th Year, University of Edinburgh Medical School	@William_ArnoldC	Protocol revisions<br>Advertising
Vinson Chan	4th Year, Leeds Medical School	@VinsonChan	Study design<br>Advertising
Keerthanaa Jayaraajan	5th Year, Imperial College London	-	Protocol revisions<br>Advertising
Veeru Kasivisvanathan	NIHR Academic Clinical Lecturer in Urology and Fellow in Uro-oncology, University College London and UCLH	@veerukasi	Protocol creation<br>Overall supervision
Sinan Khadhouri	Urology SpR, East of Scotland, Aberdeen Royal Infirmary, University of Aberdeen	@MrSinanK	Protocol revisions<br>REDCap survey creation
Chon Meng Lam	Foundation Doctor, Wales	@ChonMengLam	Protocol revisions
Alexander Light	Academic Foundation Doctor, Cambridge	@AJWLight	Protocol revisions<br>Advertising
Melissa Matthews	2nd Year, Hull York Medical School	@MelissaAtten	Protocol creation
Alexander Ng	5th Year, UCL Medical School	@AlexBCDNg	Protocol revisions<br>Advertising<br>LEARN lead
Ian Pearce	Consultant Urologist Editor in Chief, Journal of Clinical Urology	@IPearce82	Protocol revisions<br>Expert external reviewer

Copyright ©2022 BURST Research Collaborative

2. Project timeline

Dates	Description
2nd August 2019	Protocol version 1.0 created
3rd August 2019 - 16th July 2020	Protocol revisions and finalisation of REDCap survey
July-August 2020	REDCap survey pilot
July-August 2020	Recruitment of medical school leads
July-September 2020	Recruitment of foundation year 1 leads
August-September 2020	Recruitment of year group leads
3rd October-20th December 2020	Data collection
January-February 2021	Data analysis and cleaning
March-May 2021	Manuscript write up
June 2021	Conference and journal submissions

Copyright ©2022 BURST Research Collaborative

3. Background

3.1. Introduction

Urology is a prominent surgical specialty, with almost 10% of consultant surgeons employed in the specialty [1]. Urological conditions account for approximately 25% of acute surgical referrals, 10 to 15% of general practitioner appointments [2] and over 2% of all accident and emergency department attendances [3]. Urology is also a common rotation for Foundation Year 1 and 2 (FY1/2) doctors across the country, with a consistently competitive entry at specialty trainee (ST3) level [4]. Furthermore, ward-based urological issues for example urinary retention and subsequent requirement for catheterisation are common and may be encountered by junior doctors across various specialties.

Despite the prominence of the specialty and prevalence of urological issues, it is underrepresented within British medical school curricula compared to other specialties. In 2012, the British Association of Urological Surgeons (BAUS) published a national medical student urology curriculum as a point of reference for the "interested" individual and for medical schools to utilise and implement [2]. It required medical students to understand the presentation and management of various common urological conditions including haematuria, urological abdominal pain, and male infertility. This national curriculum also emphasises the importance of practical skills, including performing procedures like digital rectal examination and catheterisation, as well as observing surgical procedures such as laparotomies and endoscopic surgery. The national curriculum is an important reference for the skills and knowledge expected of new doctors, in addition it is vital to ensure students receive sufficient clinical exposure to allow them to consider it as their chosen career path.

Although the existence of a national curriculum is crucial for medical education, the extent of its uptake within medical schools across the United Kingdom is unclear. The aim of our study is therefore to assess the compliance with the updated BAUS curriculum amongst medical schools and to ascertain to what extent and how medical students are receiving training in urology. This will be achieved through a collaborative research model designed by BURST.

3.2. The BURST Research Collaborative

Founded in 2015, the British Urology Researchers in Surgical Training (BURST) Research Collaborative is a trainee-led United Kingdom (UK) research group comprising primarily of Urological Registrars and medical students, with a common aim to produce high impact multi-centre audits and clinical research that can improve patient care.

As an individual, it is often difficult to produce high impact research in a short space of time. However, through collaboration, the numerous tasks and workload of a study concept, design, planning and execution can be shared out amongst members of the group. All these collaborators will also benefit from any research output as they have collaborative authorship.

Moreover, the research collaborative facilitates the exchange of ideas and skills between the group, allowing for methodologically robust research by peer review, and development of research competencies which can be required for that individual's continuing professional development. Therefore, the use of a research collaborative can result in a high powered, high impact study delivered in a short space of time.

This has been demonstrated in 2 international collaborator studies that BURST has now successfully delivered:

Copyright ©2022 BURST Research Collaborative

- MIMIC – a multi-centre cohort study looking at the predictors of spontaneous stone passage using over 4,000 patient records in 71 sites, across 7 different countries in 6 months
- IDENTIFY – the largest study of haematuria in secondary care ever performed. As a global prospective observational study, it individualised investigations for haematuria using over 11,000 patient records in 110 sites, across 26 countries in 1 year.

Having done this, we are able to deliver this urology curriculum audit as a group of trainees and medical students spread across the UK. We plan to do this by enlisting the assistance of medical students and assigning medical student representative positions at the universities' training hospitals. Then using an email directory of medical schools across the UK, we will collect data from medical students in all years, as well as foundation year 1 doctors. Through this, we hope to carry out a thorough urology curriculum review.

3.3. Rationale

Medical school curriculum guidelines
At present each UK medical school (Appendix A) delivers the undergraduate urology curriculum differently and as such the teaching content and amount of time dedicated to the teaching of urology differs also. The undergraduate curriculum is generally based on that set out by BAUS in 2012 [2], the Royal College of Surgeons' National Undergraduate Curriculum in Surgery [5] and the General Medical Council's Outcomes for Graduates [6]. From this set of guidelines, UK medical schools use means such as lectures, problem based learning, seminars and clinical exposure to deliver the urology curriculum.

General medical school urology curriculum
UK medical schools deliver the BAUS undergdaute syllabus in different ways. For example in the 2018-2019 curriculum at University College London Medical School, in addition to non-urology specific surgical competencies such as surgical safety, consent and understanding wound healing, medical students are required to be familiar with [5][7][8]:

- Common presentations involving urinary symptoms such as bladder outlet conditions, incontinence and loin pain
- Urological emergencies such as testicular torsion, urinary retention, upper & lower urinary tract trauma, haematuria, urosepsis, priapism, paraphimosis and renal colic; to be familiar with the initial steps of management
- Urinary tract examination including genitals & prostate and being able to distinguish common pathologies such as hydroceles from a testicular tumour and benign prostatic enlargement from prostate cancer
- The indications for urological investigations such as CT-KUB, flexible cystoscopy, urinary flow rate study and urinary tract ultrasound
- Clinical skills such as catheterisation in both males and females and demonstrate familiarity with basic catheter care

The above is achieved through classroom sessions as well as ward-based teaching which includes clerking patients and formulating differential diagnoses and management plans which are to be discussed with clinicians to consolidate learning. In addition, the 2018-2019 University College London Medical School curriculum provides a non-exhaustive list of core conditions that students should be familiar with which include [7][8]:

- Benign prostatic hyperplasia
- Cancer - prostate, testes, bladder and urothelium, kidney and Wilms tumour
- Erectile dysfunction
- Gonadal dysgenesis
- Microscopic haematuria

Copyright ©2022 BURST Research Collaborative

- Phimosis, paraphimosis, balanitis
- Renal transplant
- Scrotal pain/swelling
- Testicular problems including maldescent and torsion
- Upper urinary tract obstruction, hydronephrosis
- Urinary calculi: renal, ureteric & vesical
- Urinary incontinence
- Urinary tract infection, pyelonephritis & epididymitis
- Vesico-ureteric reflux and nephropathy

For each of the core conditions above, the medical school requires students to be familiar with its epidemiology, aetiology and pathogenesis, presentation —– symptoms and signs, investigations and diagnosis, management options and public health and prevention issues.

4. Funding

Major costs associated with this project will be administration, data management, IT costs, statistical support/analysis, marketing, and dissemination. These costs will be covered within the BURST Research Collaborative with consideration for further funding.

Copyright ©2022 BURST Research Collaborative

5. Study design

Retrospective multi-centre audit of urology curricula across UK medical schools, compared against the BAUS 2012 undergraduate syllabus for urology.

6. General methods

During the 2020/21 academic year, Year 2 to Year 5 (final year) medical students at UK medical schools will be invited to complete a REDCap survey. Foundation Year 1 doctors across the UK will also be invited to complete the survey based on their graduating medical school. We will assess the student's exposure to urological teaching during their time in medical school thus far. The survey will be designed in REDCap – hosted at University College London through the academic position of Mr. Veeru Kasivisvanathan. This survey will be based on the national medical student urology curriculum published by BAUS in 2012 [2].

Each medical school will have a named student representative (medical school lead). With their assistance, we will invite at least one student lead per year group (year group lead). Year group leads will be responsible for recruiting a minimum of 15 students in their year (15 students for each hub site if there are multiple campuses) to complete the REDCap survey. Where there is more than one year group lead applicant per year group, each lead will be required to recruit a minimum of 15 students each. Medical school leads will also be responsible for retrieving the medical school's curriculum(s) used by different year groups as an aide memoire for the students completing the survey. Medical school leads will also act as a year group lead for their respective year group.

Foundation Year 1 leads will be responsible for recruiting at least 15 Foundation Year 1 doctors at their hospital/deanery.

We anticipate the redcap survey will take ten minutes to complete. Medical school leads will not be required to spend more than five hours of their time to contact individuals in their medical school, engage them in the audit and contact their course directors.

Copyright ©2022 BURST Research Collaborative

7. Study population

LEARN is open to any medical student at any medical school in the UK that performs under the GMC's and Medical Schools Council's guidelines. LEARN is also open to any Foundation Year 1 doctor who graduated from a UK medical school, and who is currently working at a UK hospital.

7.1. Inclusion criteria

- Students between their second and final year enrolled in a medicine course at a UK university which performs under the GMC's and Medical Schools Council's guidelines.
- Foundation Year 1 doctors that have graduated from a UK university which performs under the GMC's and Medical Schools Council's guidelines.

7.2. Exclusion criteria

- Students in their first year, enrolled in a medicine course at a UK university which performs under the GMC's and Medical Schools Council's guidelines.
- Foundation Year 2 doctors or more senior.
- Students at medical schools outside of the UK.

Copyright ©2022 BURST Research Collaborative

8. Objectives

8.1. Primary objective

- To compare current urology teaching in medical schools across the UK to the BAUS recommended undergraduate syllabus [2].

8.2. Secondary objectives

- To ascertain the method of delivery of urology modules (tutorials, lectures, bed-side teaching etc.) to medical students in the UK
- To assess the exposure to urology procedures
- To assess the confidence of students in the management of the required urology conditions at Foundation Year 1 level
- To assess the confidence of students in their ability to perform urological procedures as required by BAUS curriculum and GMC
- To quantify extra self-selected urology modules (including e.g. student selected components) available in each year at different medical schools
- To assess the likelihood of medical students choosing urology as a placement during foundation training
- To assess if the overall exposure to urology during medical school is sufficient to consider urology as a career option

Copyright ©2022 BURST Research Collaborative

9. Outcomes

9.1. Primary outcome

- The proportion of the BAUS undergraduate syllabus covered by medical schools, per year group, across the UK.

9.2. Secondary outcomes

For each year group and overall during medical school:

- The number of sessions of theory-based teaching delivered as lecture-based teaching, small group, problem-based teaching and anatomy teaching.
- The number of sessions of clinical skills teaching delivered as objective structured clinical examination (OSCE), bedside teaching and tutorial-based learning.
- The number of sessions undertaken during a clinical attachment in urology.
- The perceived confidence of students in urological examinations and in the management of urological conditions at the competence level expected of a Foundation Year 1 doctor.
- The perceived confidence of students in performing male and female urethral catheterisation
- Impact of Covid-19 on urology teaching during the pandemic.
- The number of extra self-selected urology modules undertaken by medical students.
- The proportion of medical students who would consider doing a urology placement during their foundation training.
- The proportion of students who have considered urology as a future career option.

Copyright ©2022 BURST Research Collaborative

10. Data

10.1. Registration

Once the medical school leads have registered their participation with the LEARN team and have been granted approval to take part, they may begin recruiting year group leads. Medical school leads will also source their university's curricula.

10.2. Data quality

Following data collection, data sufficient to determine the primary outcome, will be accepted for the study. If the quality of data submitted is deemed inadequate for the purposes of the study by the steering committee then it is at their discretion to exclude that data. Excluded participants will not be accredited in any academic outputs.

10.3. Data management

A member of BURST dedicated to data management will be required. The steering committee will design and produce the database. The data manager will manage user accounts, respond to data requests, check data for quality and completeness, communicate with sites regarding quality and completeness, answer queries from sites, produce data reports, modify and help design the database and undertake data cleaning and data wrangling as required.

Copyright ©2022 BURST Research Collaborative

11. Authorship

The main authorship line will be composed of individuals in the writing group or steering committee or reviewers who have contributed in a major way to the audit. We will recognise Medical School Leads, Year Group Leads and Foundation Year 1 Doctor Leads that have recruited at least 15 participants, as PubMed-indexed collaborators under 'British Urology Researchers in Surgical Training (BURST) Collaborative LEARN Study Group'. Position in the collaborator list will be determined by the number of students recruited to complete the survey. The greater the number of participants recruited, the higher up in the collaborator author list they will appear. We will invite the top recruiting Medical School, Year Group and Foundation Year 1 Doctor Leads onto the main authorship list.

If they choose, all individual respondents to the survey will be mentioned by name in the appendix of the paper. Respondents will also be issued a certificate to confirm their participation. This will allow respondents to show engagement and participation with a national audit project which is a key pillar of clinical governance, as well as early dedication to surgery and urology, which may be useful in a foundation and specialty training application form.

12. Dissemination

We aim to present our work at the BAUS Annual Scientific Meeting in 2021, as well as other national and international scientific conferences related to urology, surgery and education. We also expect medical student leads to present or disseminate results pertaining to their own medical school to their course leaders. Local data in this regard will be made freely available to the medical school leads.

Subject to successful peer review, we aim to publish our results in BJU International. We also aim to present our findings to BAUS with the aim of updating the BAUS Undergraduate Syllabus for Urology that was released in 2012.

13. Ethics

LEARN (uroLogical tEAching in bRitish medical schools Nationally) is a national audit and does not involve access to or collection of private or sensitive data. According to the Health Research Authority (HRA) toolkit, this study does not require NHS REC review (http://www.hra-decisiontools.org.uk/ethics/), "as long as the information you enter is correct, the outcome of these decision tools can be taken as authoritative, and you do not need to seek further confirmation."

14. Acknowledgements

With thanks to Mr Rami Issa and Mr Benjamin Ayres of St. George's University of London for their contribution to the knowledge of medical school urology curricula.

Copyright ©2022 BURST Research Collaborative

Appendix A: UK medical schools

Number	University long name	University short name
1	University of Aberdeen School of Medicine and Dentistry	Aberdeen
2	Anglia Ruskin University School of Medicine	Anglia Ruskin
3	Aston University Medical School	Aston
4	Barts and The London School of Medicine and Dentistry	Barts
5	University of Birmingham College of Medical and Dental Sciences	Birmingham
6	Brighton and Sussex Medical School	Brighton and Sussex
7	University of Bristol Medical School	Bristol
8	University of Buckingham Medical School	Buckingham
9	University of Cambridge School of Clinical Medicine	Cambridge
10	Cardiff University School of Medicine	Cardiff
11	University of Dundee School of Medicine	Dundee
12	Edge Hill University Medical School	Edge Hill
13	The University of Edinburgh Medical School	Edinburgh
14	University of Exeter Medical School	Exeter
15	University of Glasgow School of Medicine	Glasgow
16	Hull York Medical School	Hull York
17	Imperial College London Faculty of Medicine	Imperial
18	Keele University School of Medicine	Keele
19	Kent and Medway Medical School	Kent and Medway
20	King's College London GKT School of Medical Education	King's College London
21	Lancaster University Medical School	Lancaster
22	University of Leeds School of Medicine	Leeds
23	University of Leicester Medical School	Leicester
24	University of Liverpool School of Medicine	Liverpool
25	University of Manchester Medical School	Manchester
26	Newcastle University School of Medical Education	Newcastle
27	Norwich Medical School	Norwich
28	University of Nottingham School of Medicine	Nottingham
29	University of Nottingham - Lincoln Medical School	Lincoln

Copyright ©2022 BURST Research Collaborative

30	University of Oxford Medical Sciences Division	Oxford
31	Plymouth University Peninsula Schools of Medicine and Dentistry	Plymouth
32	Queen's University Belfast School of Medicine	Queen's University Belfast
33	University of Sheffield Medical School	Sheffield
34	University of Southampton School of Medicine	Southampton
35	University of St Andrews School of Medicine	St Andrews
36	St George's, University of London	St George's
37	University of Sunderland School of Medicine	Sunderland
38	Swansea University Medical School	Swansea
39	University of Central Lancashire School of Medicine	Central Lancashire
40	University College London Medical School	UCL
41	University of Warwick Medical School	Warwick

Copyright ©2022 BURST Research Collaborative

References

[1] NHS Digital. NHS Workforce Statistics, May 2019 Doctors by Grade and Specialty. Available from: https://files.digital.nhs.uk/BF/E935CA/NHS%20Workforce%20Statistics%2C%20May%202019%20Doctors%20by%20Grade%20and%20Specialty.xlsx [Accessed 28th August 2019].

[2] The British Association of Urological Surgeons. Undergraduate Syllabus for Urology. Available from: https://www.baus.org.uk/professionals/sections/undergraduate_syllabus_for_urology.aspx [Accessed 28th August 2019].

[3] NHS Digital. Hospital Accident and Emergency Activity, 2017-18; Tables. Available from: https://files.digital.nhs.uk/EC/2236E9/AE1718_national_data_tables.xlsx [Accessed 28th August 2019].

[4] Health Education England. 2018 Competition Ratios. Available from: https://specialtytraining.hee.nhs.uk/Portals/1/Content/Resource%20Bank/Competition%20Ratio%27s/Competition%20Ratios%202018.pdf [Accessed 29th August 2019].

[5] The Royal College of Surgeons of England. National undergraduate curriculum in surgery [Internet]. London: RCSENG - Professional Standards and Regulation; 2015 [cited 1 September 2019]. Available from: https://www.rcseng.ac.uk/library-and-publications/rcs-publications/docs/national-undergraduate-curriculum-in-surgery/ [Accessed 29th August 2019].

[6] General Medical Council. Outcomes for Graduates [Internet]. London: General Medical Council; 2018 [cited 1 September 2019]. Available from: https://www.gmc-uk.org/-/media/documents/outcomes-for-graduates-a4-6_pdf-78952372.pdf [Accessed 29th August 2019].

[7] UCL Medical School. Year 5 study guide 2018-2019. London; 2018.

[8] UCL Medical School. Year 6 study guide 2018-2019. London; 2018.

Copyright ©2022 BURST Research Collaborative

Appendix 5: List of Survey Respondents

A Atkinson, A O'Donald, A Panesar, A Park, Aadam Aziz, Aadham Mumtaz, Aadith Selvamohan, Aaditya Dayal, Aaditya Sanjay Dravid, Aaida Eghbal, Aakanksha Mahesh Shenoy, Aakriti Shah, Aalap Asurlekar, Aaleen Aizad, Aalia Ghafoor, Aanu Ajayi, Aarij Elahi, Aarinola Ayo-Ipaye, Aaro Kari, Aaron Briscoe, Aaron Campbell, Aaron Khan, Aaron Soane, Aaron W Pritchard, Aarti Patel, Aarya Ravindran Nair, Aashna Bali, Aashna sikka, Aasiya Chaka, Aastha Singh, Aathishree Vanji Paramasivan, Aayush Rai, Aayushi Singal, Abanoub Fidal, Abbas Esufali, Abbas Hussain, Abbey Bracken, Abbey Smith, Abbey-Louise Matthews, Abbi Bow, Abbie Evans, Abbie Farrow, Abbie McGuire, Abbie Parish, Abbie Tomlinson, Abbie warden, Abbie Wilding, Abby Radcliffe, Abd AlRahman AlMeshali, Abdal Qadir Zafar, Abdal-Aziz Agbabiaka, Abdallah Naji, Abdel Rahman Osman, Abdelmoaz Warayet, Abdul Ali, Abdul Wasi Siddique, Abdullah Al Sakiti, Abdullah Chaudhry, Abdullah Egiz, Abdullah El-badawey, Abdullah Raja, Abdulrahaman Alrashed, Abdulrahman Shandala, Abdurrahman Baden, Abdurrahman Hazrin Fazail, Abeera Devasar, Abeku Koomson, Abhilasha Yadav, Abhinav Balasubramanian, Abhinav Bhatia, Abhinaya Chandrasekaran, Abhishek Srivastava, Abi Butt, Abi Harrison, Abi Wallace, Abigael Joy Glover, Abigail Baird, Abigail Cheng, Abigail Clynch, Abigail Davis, Abigail Dawson, Abigail Dove, Abigail Duckett, Abigail Ezekiel, Abigail Gartland, Abigail Hodgson, Abigail Kemp, Abigail montgomery, Abigail Proctor, Abigail Rees, Abigail Reynolds, Abigail Richards, Abigail Smith, Abijan Pakiyaraja, Abin Varghese, Abinaya Lingeswaran, Abirami Kathiravelupillai, Abiramie Ravindiran, Abokor Mohomed, Abraham Selvakumar, abyrami sivanandarajah, Adam Andreani, Adam Carter, Adam Davie, Adam Gittins, Adam Hereward, Adam Heyes, Adam Hounat, Adam Ijaz Ali, Adam Ismail, Adam Khan, Adam O'mara, Adam Rajkowski, Adam Saeed adam, Adam Selway, Adam Townson, Adam Vaughan, Adam Wolowczyk, Adanna Anomneze-Collins, Adanna Ewuzie, Adar Butt, Adele Atkins, Adham Moftah, Adi Pusalkar, Adil Iqbal, Adithiya Janani, Aditi Pandey, Aditya Dhiran, Aditya Gaur, Adrian McGrath, Adriana Evans, Adriel Heilong Fung, Adrienne Ama Jonathan, Adrija Bhattacharyya, Advait Upadhyaya, Adwaith Venugopal, Adya Rai, Affan Salman, Afia Hossain, Afra Akmal, afrah shibu, Afroze Yousaf, Aftab mahmood, Afzaal Uwisekhan, Agata Oliwa, Agilandiswari Arumuga Jothi, Agiya Tadros, Agnel Aliyath, Ahaan Sanjay Gupta, Ahad Khan, Ahamed Marzoon, Ahmad abul, Ahmed Alhussni, Ahmed Awan, Ahmed Aziz, Ahmed Kambal, Ahmed Kouta, Ahmed Shatwan, Ahmer Khan Amran, Ahwab Alam, Ai Ng, Ai Shern Tan, Aidan James Murray, Aihem Johar, Aikaterini Markogiannopoulou, Ailidh Ramsay, Ailie McKinty, Ailsa Collar, Aiman Aslam, Aiman Dilnawaz, Aimee Kirkup, Aimee Lawton, Aimee Wilkinson, Airlie Mason, Aisel Abbasova, Aisha Ahmad, Aisha Anver, Aisha Chowdhury, Aisha Hussein, Aisha Zahid, Aishwarya Korsapati, Aishwarya Sharma, Aisling Barr, Aisling Brinn, Aisling O'Connell, Aislinn Toner, Aiswarya Modi, Aiswarya Modi, Ajanthan J, Ajanthiha Karunakaran, Akanksha Sarma, Akash Ajaya Gujjar, Akash Kennedy Ganesh, Akash Srinivasan, Akhila Panda, Akhila Sibi George, Akifah Mojadady, Akila Shakir, Akshat Sinha, Akshatha Daniel, Akua Crankson, Akvile Nikitina, Akwasi Bamfo-Quaicoe, Alakh Konjengbam, Alan Mathew, Alan Sigmand, Alan Yap Shao Hao, Alanna Wyncoll, Alasdair Sandland, Alastair Woodhead, Alaynah Nawaz, Alba Eisner, Alec McArdle, Alec Rapson, Alec Vinh, Aled McCluskey, Aleema Sardar, Aleena Madathiparambil Saju, Aleena Thomas, Alejandra G Ten, Alejandra Martin Segura, Alekh Thapa, Aleksander Dawidziuk, Aleksandra Goch, Aleksandra Marcinkiewicz, Aleksi Simons, Alessia Ballario, Alessia Kostiw, Alessia Tarantino, Alex Ainger, Alex Barrie, Alex Brookmyre, Alex Calder, Alex Dior, Alex Hanson, Alex Kwok, Alex Lee, Alex Michael Summerbell, Alex Moore, Alex Morelli-Batters, Alex Murigu, Alex Patterson, alex ransome, Alex Satei, Alex Shortall, Alex Teasdale, Alex Tze Kin Ng, alex whitworth, Alexander Albert Dermanis, Alexander Anderson, Alexander Bloom, Alexander Browne, Alexander Cole, Alexander David Pirmohamed, Alexander Dereham, Alexander Gonzalez-Lamberth, Alexander Hawkins, Alexander Hunt, Alexander Irving, Alexander James Matthews, Alexander Jaques, Alexander Kawalec, Alexander MacKinnon, Alexander Oh, Alexander Palmer, Alexander S C West, Alexander Smith, Alexander Stone, Alexander Thomas, Alexander Tough, Alexander Turner, Alexander Valnarov-Boulter, Alexander Watson, Alexandra Busch, Alexandra Cardoso Pinto, Alexandra Celeste Smith, Alexandra DeSarno, Alexandra Franiek, Alexandra G Irvine, Alexandra Jenkins, Alexandra Kontothanassis, Alexandra Langford, Alexandra Makrides, Alexandra Mary Irving-Wilson, Alexandra McNeil, Alexandra McNish, Alexandra Minseo Kim, Alexandra Murphy, Alexandra Rawlings, Alexandra Smyth, Alexandra Stanley, Alexandra Warren, Alexandra Zmuda, Alexandrina Braniste, Alexis Adam, Alfred Cuddeford, Alfred Yeo, Ali Abdelaal, Ali Akber Rajani, Ali Alhashimi, Ali Al-Hussaini, Ali Al-Sakban, Ali Asad, Ali Hamad, Ali Khalid, Ali Kia, Ali Yalcinkaya, Alia Sahni, Aliah Nosheen Mian, Alice Barber, Alice Bradley, Alice brown, Alice Cato, Alice Cepelowicz Lessa, Alice Churchill, Alice Curzon, Alice Dewhirst, Alice Ditchfield, Alice Furness, Alice Glen,

Alice Halliday, Alice Hanton, Alice Irving, Alice Ismail, Alice Jones, Alice Kate Lively, Alice MacNeill, Alice Maguire, Alice Marie Watts, Alice McIlveen, Alice Mines, Alice Murphy, Alice Neilson, Alice Oatham, Alice Ostojic, Alice Pryke, Alice Richens, Alice Ridley, Alice Rogers, Alice Rolph, Alice Solerod, Alice Turnock, Alice Watson, Alice Winch, Alice Yates, Alicia Aree Polley, Alicia Griffin, Alicia Halsall, Alicia Micklefield, Alicia Paessler, Alicia Schwarzenbach, Alicia Tickle, Alicia Titouche, Alif Aziz, Alika Dijkstra, Alima Yasmin, Aliraza Syed, Alireza Majlessi, Alisha Ali, Alisha Kanani, Alisha Lal, Alisha Pati-Alam, Alisha Ruparelia, Alison Marie Lee, Aliya Syahreni Prihartadi, Aliyah Ahmad, Aliyah Khan, Alizée Baumert, Alka Mary Biju, Allegra Wisking, Allen Isaac Royal, Alna Dony, Alokya Balagamage, Altay Shaw, Alvi Akter, Alyaa Mostafa, Alysha Binti Mohamed Harun, Alysha cooper, Alyssa Van den Broeck, Alzahra Kassir, Amaad beg, Amaan Abbas, Amaani Hassan, Amal Adlan, Amal Robertson, Amalie Bjurhult Kennedy, Aman Saini, Aman Singh, Amana Siddique, Amanda Godoi, Amanina Ayunni binti Ahmad Alawi, Amanpal Sidhu, Amanpreet Kaur, Amar Mann, Amar Singh Bhogal, Amarah Mirza, Amarah Saeed, Amarta Jaipal, Amber Ahmed-Issap, Amber French, Amber Glenn, Amber Johnston, Amber Knapp-Wilson, Amber Newman-Marks, Amber Oldham, Amber Pankhurst, Amber Torkington, Ame Saidy, Ameena akhtar, Ameena Suleman, Ameeta Kumar, Amelia Dawson - Kavanagh, Amelia Edmondson, Amelia Fraser-Dale, Amelia Lancaster, Amelia Redman, Amelia Seifalian, Amelia Sheppard, Amena Al Saad, Amina Adan Mulata, Amina Ali, Amina Aslam, Amina Mushtaq, Aminah Ashraf, Aminah B Mirza, Aminah Hussan, Aminah Khan, Aminath Izfa Amir, Amir Fard, Amir Habeeb, Amir Khalil, Amir Rashid, Amirah Latief, Amisha Kalra, Amit Arora, Amit Bhudia, Amit Nayak, Amit Singh Atwal, Ammaarah Burani, Ammar Siddiqui, Ammara Shaheen, Ammena Zahabi, Amolemo Keitumetse, Amon Kenna Ette, Amordei Aghanenu, Amrit Hayre, Amrit Kaur Mann, Amrit Kaur Sanghera, Amrita Badanahatti, Amrita Rai, Amrutha Koppula, Amy Bancroft, Amy Bullock, Amy Calow, Amy Coats, Amy Cox, Amy Craig, Amy Cromie, Amy Edwards, Amy Evans, Amy Fisher, Amy Gatton, Amy Gosling, Amy Grant Moreland, Amy H Morris, Amy Handley, Amy Irvine, Amy Johnston, Amy Jones, Amy Lebby, Amy Li, Amy Lillington, Amy Noble, Amy Potts, Amy Rana, Amy Szigeti, Amy Wright, Amy-Leigh King, Ana Alina Haiduc, Ana MacConnachie, Anahita Dadali, Anam Asad, Anam Choudhry, Anam Jawaid, Anandna Bhatia, Ananya, Ananya Bhardwaj, Ananya Gopalakrishnan, Ananya Nair, Anas Aboukoura, Anas Bara, Anas hatab, Anastasia Constantinou, Anastasia Efstathiou, Anastasia Oh, Anasua Das, Anavi Prakash, Anca-Mihaela Vasilica, Andra Dobromirescu, Andre Agyepong, Andre C. Q. Lo, Andre Chu, André Tulloch, Andreas Eleftheriou, Andreas Matheou, Andreas Mirallais, Andreea Gray, Andrew Allman, Andrew Brown, Andrew E Morrish, Andrew Holliman, Andrew Jackson, Andrew McGaughey, Andrew O'Neill, Andrew Thomas Brennan, Andrew Wiggam, Andrew Wilson, Andria Kantharuban, Andrzej Luckiewicz, Andy Howard, Aneesa Khatija Hameed, Aneesah Bashir, Aneesah Khan, Aneeshka Nagpaul, Anesha Javed, Angad Singh, Anganile Mercy Mwanjoka, Angela Huang, Angela Kathiraman, Angela Ogunrinu, Angelica Dahil, Angelica Sharma, Angelica Spence, Angeliki Danai Andrianopoulou, Angelina Cham, Angeline Pesala, Angelo Alonzo-Beckett, Angelus Cyrus, Angharad James, Angus Higgins, Angy Apata, Anika Madaan, Anika Sharmila, Animesh Jayaswal, Aniqa Tasnim Haque, Aniquw Burke-Robinson, Anisah Ahsan Khan, Anisah Ali, Anisah F Ali, Anish Kundu, Anisha Wakefield, Anita Adiga, Anita Ravikrishnan, Anitha Balaskandarajah, Anja Powell, Anjalee Chaurasia, Anjali Kanyaka Dias Samarawickrama Yapa, Anjali Patel, Anjali Shah, Anjali singh, Anjan Nibber, Anjana Lakshmi Narasimhan, Anjola Adeniran, Anjolaoluwa Sofuyi, Anjuli A Banerjee, Anjuli Clough, Ankeet Tanna, Ankit Gupta, Ankita Gupta, Anmol Jaiswal, Ann Alexander, Ann Curtis, Ann Minal Dominic, Ann Panjikkaran, Anna Adlem, Anna Armstrong, Anna Beak, Anna Bean, Anna Blois, Anna Campbell, Anna Cartledge, Anna Casey, Anna Chappell, Anna Dickson, Anna Douthwaite, Anna Farwah, Anna Finnegan, Anna Freedman, Anna Gallagher, Anna Jade Rama, Anna Jiang, Anna Jones, Anna King, Anna Kruczynska, Anna Kyriakidou, Anna Leigh, Anna Loader, Anna Marshall, Anna Martyna Sozanska, Anna Povall, Anna Smith, Anna Swart, Anna Tzortzi, Anna Vitória Pivotto Flávio, Anna Wakelin, Anna Wallace, Annabel Harriman, Annabel Killen, Annabel Skinner, Annabel Ward, Annabelle Hook, Annabelle Jarjis, Annabelle Smith, Anne Arputharaj, Anne Oshodi, Anne Ramos, Anneesa Malik, Anne-Marie Gabrawi, Annette Antony, Annie Berry, Annie Cheung, Annie Evans, Annie Henderson, Annie Morris, Annie Ng, Annie O'Boyle, Annie Rees, Annie Renju, Anoop Singh Sumal, Anouk Wijeratne, Anoushka Devi Bucktowar, Anoushka ramkumar, Ansa Sunil, Ansana Adam, Anshu Arora, Anshul Aich, Anthony Barlow, Anthony Feeney, Anthony Henein, Anthony Sinobas, Anthony Wijaya, Anthony Yew-Kheen Tang, Antoni Bochinski, Antonia Ashaye, Antonia Ramona Perumal, Antonio Maria Borges Neves, Antony Antypas, Antony Ben Decruz, Antony lavercombe, Anu Mathew, Anuj Bhatnagar, Anuj Neelesh Gupta, Anum Choudry, Anusha Leonard, Anushae Akhtar, Anushka John, Anushka Mohan, Anushka Pathak, Anushri Joshi, Anushruti Yadav, Anvay Deshpande, Anya Jacqueline Rutherfurd, Anya Lawrence, Anya Slowinski, Anyanna Harker, Aoibhin McKenna, Aoibhinn Murray, Aoife Bannon, Aoife Gilbride, Aoife Maya Janmohamed, Aóife

Moffatt, Aos Al Hasani, Aparna Anoop, Aparna Biju, Aparna Potluru, Aphra Luchesa Smith, Apoorva Dudani, Apoorva Govil, Aqeeb Mahmood, Aqib Chowdhury, Aqsa A Chughtai, Aqsah Tariq, Aquiline Lobo, Arabella Atkins, Arabella Zuckerman, Aradhana Topiwala, Aravind Manoj, Archit Singhal, Archith Kamath, Areeba Ansari, Areej Karim, Arfaa Butt, Argha Datta, Ari Saltmarsh, Ariadne Holmes, Ariadni Papadopoulou, Arian Rahim, Arianna Morris Gouveia, Arif Hanafi Bin Jalal, Aris Alexiadis, Arjan Jaiyeola, Arjan Singh Sehmbi, Arjun Iyer, Arjun Vyas, Arman Shahriar Haque, Armin Nazari, Arooj siddique, ARRABI VIJAYAKUMAR, Arshi Baig, Arslan Raja, Arub Aziz, Aruchana Maheswaran, Arul Lucian Arulpragasam, Arun Jassal, Aruni Mathyalakan, Arunima Basu, Arushi Ramani, Arwa Hagana, Arya Ananya Gokul, Arya Anthony Kamyab, Arya Shah, Aryan Goel, Aryan Niknam Maleki, Aryana Jizan, Asad Siddiqui, Asanish Kalyanasundaram, Asante Mbisa, Asha Chaudhry, Asha Nair Unnikrishnan, Asha Rattan, Ashani Benning, Ashka Moothoosamy, Ashleigh Blood, Ashleigh Murray, Ashleigh Reece, Ashley Balls, Ashley Berry, Ashley Coope, Ashley Elder, Ashley Solomon, Ashli Alex, Ashmal Qamar, Ashna Ashpak, Ashni Asit Badiani, Ashvanthi Sriranjan, Ashvin Kuri, Ashvin Thomas, Ashvin Virdee, Ashwin Kalyana, Ashwin Paul Bobby, Ashwin Velloresuresh, Ashwin Venkatakrishnan, Ashwin Venkatesh, Ashwini Venkatesh, Ashwitha Karnati, Asif Gardari, Asim Abbas, Asma Feroz, Asma Muhammad, Asmaa Ali, Asmithaa Prabhakaran, Astriti Vatwani, Atchchuthan Kanagasabai, Ateeq Jamil, Atilla Albatros Emin, Attam Khan, Attar Singh, Au Matthew Chun, Augustus Rottenberg , Aurisa Uchupalanun, Aurora Braka, Austin See, Austin Tse, Avani Varde, Avenie Mavadia, Avikalp Kishore, Avneet Kaur Dhandee, Avni Krupeshkumar Patel, Avni Naik, Awais Al-Hassan Ali, Awo Ahmed, Awrad Ghadanfar, Aya Asse, Aydan Rahmanova, Ayden Ismail, Ayeesha Rela, Ayesha Choudry, Ayesha Elahi, Ayesha Kazi, Ayesha Siddika, Aylin Can, Ayman Ahmed, Ayman Haider, Aynaz Dehghan, Ayolola Eni-Olotu, Ayomide Florian Nurudeen, Aysha Adil, Aysha Zahid, Ayush Gupta, Ayushi Anna Dinesh, Ayushi Singh, Azhaar, Azim Qadir, Azizah Khan, Aznavar Ahmad, B Padgett, Baasil Syed Yusuf, Badra Farah, Bailey Watson, Bako Nouri, Balamrit Singh Sokhal, Balint Borbas, Banayot Hosh, Baoying Huang, Bara'ah Hasan, Baran Firat, Baran Talajooy, Baribefe Olufemi Vite, Barirah Ashfak, Barrie Lyell, Bassant Abdelfadeel, Bavin Pathmaraj, BC, Beatrice Skene, Becky Leung, Becky Thomas, Bede Tyler, Behzad Stanekzay, Belinda Adupong, Ben Appleby, Ben Armstrong, Ben Brown, Ben Chrisp, Ben Doughty, Ben Parker, Ben Preston, Ben Sharp, Ben Shaw, Ben Tansey, Ben Turner, Ben Ward, Benedict Mallucci, Benedict Osei-Boadu, Benedict Soo, Benita Peone Thambipillai, Benjamin Drury, Benjamin Erin, Benjamin Freed, Benjamin French, Benjamin Gompertz, Benjamin Harris, Benjamin Hawkins, Benjamin Holland, Benjamin Langhorne, Benjamin Leow, Benjamin Popham, Benjamin Roobottom, Benjamin Rose, Benjamin Schoyer, Benjamin Southward, Benjamin Stainer, Benjamin Stewart, Benjamin Subhani, Benjamin Tse, Benton Pok Hei Chung, Benz Josiah, Berin NA Gorgun, Bernice Johal, Bertram Hoare, Beth Coiley, Beth Dillon, Beth Jones, Beth McCloy, Beth McMahon, Beth Webb, Bethan King, Bethan Moor, Bethan Parry, Bethany Catchpole, Bethany Clayton, Bethany Ellis, Bethany Foo, Bethany Helm, Bethany Huddleston, Bethany Jones, Bethany McLoughlin, Bethany Rose, Bethany Shiell, Bethany Stott, Bethany Sykes, Bethany Taylor, Bethany Taylor, Bethany Wildridge, Bethia Evans, Betsy Edwards, Betty Gusha, Beverley King, Bexley McCormick, Bhagyshree Patel, Bhavika Ashok Parmanand, Bhavina Patel, Bhavisha Mistry, Bhavnit Kaur, Bhumi Shukla, Bhuvana Mandadi, Bianca Botezatu, Bianca Buencamino, Bihu Malhotra, Bilaal Ghafoor, Bilal Korimbocus, Bilal Suleman, Bipima Gurung, Blanca Pamias Lopez, Blanca Sanz-Magallon, Bolin Dai, Bony Roy, Boris C Wagner, Botond Levente Kaholics, Bradley Pearce, Bradley Pittam, Branavy Somasundaram, Brandon Yeo, Brawin Kajenthra, Breanna Blackboro, Brian Moosa, Brianna Robinson, Bridget Kemball, Briony Seden, Brishti Debnath, Brittany Blackstone, Brittany Brownlee, Brogan Butler, Bronagh Donaghy, Bronwyn Woodburn, Brooke Cannon, Brooke Gerrie, Brooke Maciejewski, Brooklyn Wright, bruce craig, Bryan Chng, Bryan Sim Kwong Yee, Burhan Waheed, Buzz Gilks, C J Atkinson, Caelan Bains, Caera Douglas, Caio Guerra Hansen, Caitlin Allwin, Caitlin Blackwood, Caitlin Bryant, Caitlin Christie, Caitlin Fisher, Caitlin Foley, Caitlin Hayes, Caitlin McCleary, Caitlin Murphy, Caitlin Pollock, Caitlin Rock, Caitlin Smith, Caitlyn Jodie Warren, Caleb Hariri, Callum Alexander, Callum Edmonds, Callum Eley, Callum Leng, Callum Verran, Calum Connolly, Calum Mooney, Calum Rhys Williams, Calum Taranjit Singh, Calvin MacKinnon, Cameron Bonthrone, Cameron Carty-Barjonas, Cameron Fleming, Cameron J Stephen, Cameron Khakh, Cameron Leiper, Cameron Martin, Cameron Whitley, Camille Jenkins, Caoilin Marstrand, Caoimhe Armstrong, Caoimhe Kelly, Caprice Pawsey, Cara Beattie, Cara Lucas, Cara Wardman, Carina Cox, Carina M. Luxhoj, Carina Synn Cuen Pan, Carla Badger, Carlos Eduardo Perez Caceres, Carlyn Kennington, Carol Lo, Carolina Brealey, Carolina Maria Campana, Carolina Rosa, Caroline Brewer, Caroline Helen Jarman, Caroline Lowman, Caroline McKernan, Caroline Millett-Spicer, Carolyn Chiam, Casril Liebert, Cassidee Eccles, Cassie Bahouse, Caterina Shepherd, Catharine Kwok, Catherine A Campbell, Catherine Beeby, Catherine Cooper, Catherine Crawshaw-Brown, Catherine E. Scott, Catherine Graham, Catherine Jones,

Catherine Levell, catherine Macleod-Hall, Catherine McIlroy, Catherine Miller, Catherine Mills, Catherine Moores, Catherine Ogbechie, Catherine Otoibhi, Catherine Oxley, Catherine Palfreeman-Watt, Catherine Scriven, Catherine Smith, Catherine Williams, Catrin Buckley, Catrin Kunemund-Hughes, Catrina Nicholl-Pierso., Catriona Walker, Catriona Webb, Cecilia Cirelli, Cecilia Gray, Cecily Bloom, Celina Akhtar, Celine Perera, Ceri Gillett, Ceri John Honey-Jones, cerys barratt, Ceyhun Aksel Oztumer, Chakshu Joshi, Chan Ai Win, Chanaradh James Poonsuph, Chandar Jaipal, Chandrarajan Shah, Chaninda Dejsupa, Chantal Corbin, Chantal Salira, Chantelle Hamman, Chantelle Waddington, Charindu RANMUTHU, Charlene Ursula Khoz, Charles Brockwell, Charles Coombs, Charles Dwan, Charles Ingram, Charles lake, Charles Meeran, Charles Seddon, Charles Simpson, Charles Travers, Charles Willes, Charlie Anderson, Charlie Davies, Charlie Gill, Charlie Price, Charlie Ryan, Charlie Soon Ming Wong, Charlotte Acheson, Charlotte Allan, Charlotte Arnold, Charlotte Buchalter, Charlotte Casey, Charlotte Cassidy, Charlotte Casteleyn, Charlotte Crook, Charlotte Drewett, Charlotte Eeckelers, Charlotte Evans, Charlotte Gemmell, Charlotte Goodrum, Charlotte Hawthorne, Charlotte Hogg, Charlotte Hope, Charlotte Lee Ruo Qi, Charlotte Leigh, Charlotte Louise Smith, Charlotte Lyon-Dean, Charlotte Marsh, Charlotte Maughan, Charlotte McCluskie, Charlotte McMullan, Charlotte McQuoid, Charlotte Miles, Charlotte Muehlschlegel, Charlotte Pearson, Charlotte Pickwick, Charlotte Richardson, Charlotte Shan Ho, Charlotte Slee, Charlotte Taylor, Charlotte Waghorne, Charlotte Wilson, Charlotte Wistow, Charumathy Dhakshinamoorthi, Chavini Ranasinghe, Chee Yen Hew, Chee Yen Soon, Chelsea Agidi, Chelsea Chan, Chelsea Stubbs, Chelsie Walters, Cheng Jun Cheong, Chenghao Huang, Cher Ying Foo, Cheuk Heng Li, Cheuk Tung Kam, Cheuk Ying Peony Kan, Chevonne Risbrooke, Chew Jun Ni, Chi Hoi Lee, Chiam Jane Yi, Chiamaka C S Uwakaneme, Chiamaka Elumogo, Chiang Shu Yu, Vanessa, Chiara Jade Vedi, Chiara Pitzalis, Chiara Rizk, Chiara Zwirner, Chidera Obuah, Chie Katsura, Chien Lin Soh, Chi-ian Kuok, Ching Ching Ho, Ching Wei Ng, Chinonyerem Umelo, Chioma Onuha, Chirag Bussa Rao, Chiranth Badrinath, Chit Wong, Chizara Lock, Chloe Baird-Rayner, Chloe Baker, Chloe Cheuk Wa Wong, Chloe Flintham, Chloe Gill, Chloë Guy, Chloe Hayes, Chloe Ho, Chloe Huvenne, Chloe Leftley, Chloe Legard, Chloe Longden, Chloé Moran, Chloe Roberts, Chloé Simela, Chloe Smith, Chloe Staniland, Chloe Stevens, Chloe Vun, Chloe Ward, Chloe Watson, Chloe Wen Li Chia, Chloe Williams, Chong Jia Cheng, Chong Li Cheng, Chow Marcus Yik Hin, Chris McColm, Chris S. Tharakan, Christian Aquilina, Christian Oldfield, Christie Hall, Christie Tsang, Christina Browne, Christina Huon, Christina Quinn, Christina Swaby, Christina Taylor, Christine Daniel, Christine Pettitt, Christopher Andrew William Gunn, Christopher Anetekhai, Christopher Barrett, Christopher Bone, Christopher Brown, Christopher Ford, Christopher Holt, Christopher Jervis, Christopher Khoory, Christopher Lawrence McCloy, Christopher Madden-McKee, Christopher Marshall, Christopher Morris, Christopher Takyi, Christopher Tuttle, Christopher Yemm, Christos Charalambous, Christos Papaioannou, Chua Yizhi, Chun Fai Yip, Chyu Lai Yan Naing, Cian Neel Che Patel, Ciara Lindsay, Ciara Mulrenan, Ciaran Fallon, Ciaran Finnegan, Cigdem Isti, Claire, Claire Brunning, Claire Horsfield, Claire Huang, Claire O'Donnell, Claire Read, Claire Williams, Clare Bartholomew, Clare Chown, Clare Dempsey, Clare Hui Gin Lau, Clare Mashford, Clarissa Lister, Claudia Loggir Vidueira, Claudia Rose Grant, Claudia Santoni, Claudia-Gabriela Mitrofan, Clement Bakina, Cliona Meenan, Coco Zhang, Cody Cassidy, Colette Thorold, Collin Mattathil, Colm Kelliher, Connie Buchanan, Connie Glover, Connie McCafferty, Connor Cosgrove, Connor Parsley, Connor Tugulu, Conor J Hardacre, Conor Smith, Constance Davidson, Constantina Stavrou, Constantinos Kapetanos, Constantinos Savva, Cora Lowe, Coralie Bell, Corinna Clark, Corlyn Lee, Courtney Brick, Courtney McAdoo, Courtney Taylor, Craig George-McDowall, Craig John Fraser, Cristina Gherghisan, Crystal Mjumi, CY Cheung, Cynthia, da wit kim, Daanish Ghaffar, Dáire Magorrian, Daisy Aje, Daisy Bevis, Daisy Blaksley, Daisy Boyle, Daisy Campen, Daisy Edgerley, Daisy Taylor-Knowles, Dakshita Agrawal, Dalia Thomas, Dalitso Mwale, Dalton Barham, Damian Jeyananthan, Dana Beretta, Dana Hutton, Dana Ramzi Haddad, Dania Mann-Wineberg, Dania Quadri, Daniah Hassan, Danial Naqvi, Daniel Ahari, Daniel Andreyev, Daniel Arbide, Daniel Bastock, Daniel Chalk, Daniel Coulson, Daniel Gallo, Daniel Ghent, Daniel Humphreys, Daniel J Warrington, Daniel J. Hern, Daniel Joseph Peck, Daniel Last, Daniel Ly, Daniel McDonald-Smith, Daniel McElroy, Daniel Monaghan, Daniel Murphy, Daniel Myers, Daniel Napier, Daniel O'Flaherty, Daniel Perry, Daniel Price, Daniel Refaat, Daniel Riding, Daniel Riley Brown, Daniel Ross, Daniel Sescu, Daniel Worthington, Daniella Fahmi, Daniella MacDonald, Daniella Soussi, Daniella Wu, Danielle Ankrah-Anobah, Danielle Dean, Danielle Durant, Danielle Gabriel, Danielle Hayllor, Danielle Joyce, Danielle Oluyomi, Danielle Solomou, Danielle Thomson, Daniyal Matin Ansari, Dara Milkova, Dardan Uka, Daria Romanyuc, Darius Oraee, Darmeena Gopikrishna, Darragh Lawlor, Darshana Jeyaruban, Darya Ibrahim, Darya Pospyelova, Daud Muhammad, David Awobem, David Bourne, David Fellows, David Griffith, David Johnson, David Manson, David Nagra, David O'Connor, David Ogunyanwo, David Warren, David Wringe, Davina McLaverty, Davina Mistry, Davog McCaffrey, Dawn Chueh,

Dayna Arthur, Debby Koo, Debora Kubel, Deborah Adepoju, Deborah Albert, Deborah Ama Brown, Deborah Charlesworth-Benedict, Deborah O Alawode, Deborah Olusanya, Declan Powe, Deepa Dodia, Deepa Sharma, Deepiksana keerthy, Deepthi Balaji, Defne Artun, Delara Hashemi, Demetra Kyriakides, Dena Al-waidh, Dennis Ammann, Derrick Fernandk, Dev Gakhar, Devadharshini Kandasamy, Devajit Das, Devavratha Muthalagappan, Devika Verma, Devon Lloyd-Morris, Devon Ward, Devya Kumaresan, Dharmveer Sharma, Dharshana Panchatsharam, Dhikshitha Gokulakrishnan, Dhikshitha Nagaraj, Dhilon Selvaratnam, Dhiraj Lehru, Dhivyaa Premachandra, Digby Hopkinson-Woolley, Dijay Dave, Diksha Bhagat, Dilara Eren, Dilen Parmar, Dilogen Muraleetharan, Dilraj Singh Bhullar, Dilshi Abeywardena, Dilys Tawiah, Dima Abdelhafiz, Dimiana Salama, Dineesha Silva, Dinushika Weerasinghe, Dion-Emily Manning, Dionne Phillips, Dionysis Skiadopoulos Seimenis, Dipali Dhiru, Disha Singhania, Diva Jhaveri, Divani Narendranathan, Divanshi, Divine Adegbie, Divine Dominic, Divya Khanna, Divya radhakrishna, Divya Suresh, Divya Thankaraj, Diya Banerjee, Doha Basiouni, Dominic Coates, Dominic Fritche, Dominic Lee, Dominic O'Neill, Dominic Treloar, Dominic Weller, Dominique J. M. Al-Hindawi, Dona Mathew, Dongha Lee, Donna Maria Johnson, Donna Thomas, Dorota Zak, Doroti Csvila, Dory-Anthony Ghanem, Douglas Canning, Drew Hawtin, Drew McMichael, Duaa Ali Faruqi, Duaa Masood Ahmed, Dulan Perera, Duleni Nimaya Gunaratne, Dunya Najat, Durodoluwa Falowo, Dwynwen Spink, Dylan, Dylan Birk, Dylan Salter-Payne, Dylan Suyama, E Crichard, Éabha Lynn, Éadaoin Anderoson, EAIDRAY PHU, Eashan Patel, Ebrahim Patel, Ebtehal Moussa, EC, Ecem Mimoglu, Ed Archer, Ed Whittaker, Eda Lyuman, Eda Sansel Ustun, Eden Bogie, Eduardo Panaiotis Deliyannis, Edward Duffy, Edward Dundas, Edward Falkingham, Edward Fall, Edward Finch, Edward Hatfield, Edward Jun-Shing Lau, Edward Kenny, Edward Lockhart, Edward McKee, Edward Richardson, Edward Robinson, Edward Yan Ming Lau, Eera Sarda, Eesa ahmed, Eesaa Longden, Efua M. Abankwa, Eilidh Fraser, Eilidh Lui, Eilidh Miller, Éilis Rowan, Eimad Basit, Eimear O'Connell, Eimen Javed, Eirini (Irena) Papapetrou, Eisha Tandon, Ekaterina Kinnear, Elaina Cayrouse, Eleanor Badhams, Eleanor Barnett, Eleanor Brain, Eleanor Brewer, Eleanor Bruce, Eleanor Burnd, Eleanor Cochrane, Eleanor Davies, Eleanor Deane, Eleanor Godhard, Eleanor Goldstein, Eleanor Harvey, Eleanor Hogg, Eleanor Hope Boyle, Eleanor Kissane, Eleanor Lucy Wedgwood, Eleanor McNally, Eleanor Mitham, Eleanor Naccarato, Eleanor Patterson, Eleanor Pettitt, Eleanor Pollard, Eleanor Renton, Eleanor Ruffle, Eleanor Spence, Eleanor Swanton, Eleanor Talbot, Eleanor Yip, Electra Botsa, Eleen Inayat, Elen Sanpher, Elen Shaji, Elena Evanson, Elena Brachimi, Elena Bunola-Hadfield, Elena Missir, Elena Perez Fernandez, Elena Priestman, Eleri Lewis, Elias Jamieson, Elif Gecer, Elin Harriet Wynne, Elina Joy, Elina Stokolova, Elina Tanskanen, Elinor Clark, Elisa Shi Wei Lau, Elisa Smith, Elisabeth Bonor, Elisabeth J Hopwood, Elisabeth Rieley, Elise Davis, Elisha De-Alker, Elitsa Palenikova, Eliza Burdass, Eliza Robinson, Elizabeth, Elizabeth Araromi, Elizabeth Bamidele Ogunbayo, Elizabeth Batchelor, Elizabeth Burley, Elizabeth Daly, elizabeth dickensn, Elizabeth Faulkner, Elizabeth Good, Elizabeth Hancock, Elizabeth Hardiman, Elizabeth Haslehurst, Elizabeth Horton, Elizabeth Jackson, Elizabeth Jane Tilley, Elizabeth Johnson, Elizabeth Kay le Roux, Elizabeth Lawson, Elizabeth Lim, Elizabeth McNeil, Elizabeth Mellor, Elizabeth Morley, Elizabeth Soffe, Ella Beard, Ella Bulgen, Ella Buluwela, Ella Davidson, Ella Finigan, Ella Hobbs, Ella J Marson, Ella Kollstad, Ella Kon, Ella Maxwell, Ella Rawlins, Ella Regester, Ella Riley, Ella Snowdon, Ella Strudley, Ella Sykes, Ella Van Den Begin, Ella Walkeden, Elle Dodds, Ellen Adair, Ellen Beattie, Ellen Bickley-morris, Ellen Campbell, Ellen Nelson-Rowe, Ellen Pauley, Ellen Peters, Ellen Sheppard, Ellen Simpson, Ellen Stanley, Ellen Talbot, Ellena Cotton, Ellie Bagshaw, Ellie Buck, Ellie Chilcott, Ellie Craven, Ellie Harding, Ellie L Taylor, Ellie McIntosh, Ellie Pearce, Ellie Rowe, Ellie Russo, Ellie Shaw, Ellie Stokes, Ellinor Raby, Elliot Rogers, Elliot Sutcliffe, Elliott Bigg, Elliott Lonsdale, Elliott Shaw, Elmahdi Darbi, Elodie Oweis, Eloise Evans, Eloise Freeman, Eloise Graham, Eloise Marsland, Eloise Radcliffe, Eloise Rogers, Eloise Sludden, Eloise Young, Elsa Harte, Elwyn Dsouza, Elyas Abaris, Elysia marshall, Eman Hasan, Emanuel Thamm, Emel Yildirim, Emelia Boggon, Emilia O'Connor, Emilia Zmudzin-Manzelov, Emilie Dommett, Emilio Aliberti, Emily Alexander, Emily Bar, Emily Broad, Emily Brooks, Emily Campbell, Emily Cao, Emily Chruscikowski, Emily Clough, Emily Coyne, Emily Crawley, Emily Densham, Emily Dias, Emily Evans, Emily Finbow, Emily Findlay, Emily Galbraith, Emily Gaskin, Emily Ghio, Emily Greenaway, Emily Grimshaw, Emily Hall, Emily Hall, Emily Hall, Emily Hepburn, Emily Herbert, Emily Hu, Emily James, Emily King, Emily Lockhart, Emily Lovell, Emily Marie Nicholson, Emily Mayar, Emily Mckeown, Emily Park, Emily Pawley, Emily R Kemp, Emily Roberts, Emily Robertson, Emily Roots, Emily Rowe, Emily Shaw, Emily Sidaway, Emily Sinha-Royle, Emily Slaine, Emily Swift, Emily Taylor, Emily Taylor, Emily Thomas, Emily Tsang, Emily Tumelty, Emily Wales, Emily Wallace, Emily Williams, Emily Woodhead, Emira Mahrin, Emma Bache, Emma Bailey, Emma Boxley, Emma Brandstatter, Emma Brune, Emma Cartner, Emma Carville, Emma Chia, Emma Doherty, Emma Ford, Emma Gull, Emma Hutchings, Emma Jackson, Emma Jane McCann, Emma Jane Norton, Emma Johnston, Emma Joy Robertson, Emma Kelly, Emma Khoury, Emma

Lewin, Emma Louise Backhurst, Emma Lowe, Emma Lyons, Emma McLeod, Emma Midgley, Emma Moore, Emma Parry-Jones, Emma Poynton-Smith, Emma Preston, Emma Sharp, Emma Shorrock, Emma Smedley, Emma Stephen, Emma Stephens, Emma Tonner, Emma Warinton, Emma Watt, Emma Whiting, Emma Williamson, Emmanuella Akhionbare, Emmanuella Yeboah, Emmanuelle Godinho, Emyr Rees, Enada Pupla, Enam Ruf, Eng O-charoenrat, Eni Jeboda, Eoin Melby, Erica Colwill, Erica Tsoi, Erin Brackenbury, Erin Jensen, Erin Kamalanathan, Erin Maynard-Connor, Erin Prentice, Erin Sweeney, Erum Aafreen Matloob Ahmad, Ervehe Gashi, Erwann Le Lannou, Esha Ali, Esha Sagar, Esha Young, Esme Cross, Esme graham, Esme Poole, Esmee Dohle, Esra, Esra Karakasli, Essi Troughton, Esther Dami Okhiria, Esther Gbinigie, Esther Goh, Esther Pinto, Ethan Goh, Ethan M Coyne, Etienne Chew, Eu Fang Foo, Euan Lewis, Euan Robertson, Eugene Chen Wen Cong, Eun Yee Hew, Eunice Lee, Eurekha Ravindran, Eva Dolan, Eva Klesnik-Edwards, Eva Nagy, Eva Papaioannou, Eva Ritchie, Evangeline Letch, Eve Christine Sealy, Eve Draycott, Eve Miller, Eve Pannone, Eve Philippides, Eve Phillips, Eve Webley, Evie Gibbs, Evie Gittings, Evie O'Rourke, Evie Tselentakis, Evie Wood, Evlyn Forsyth-Muris, Ewan Andrew McAlpine Pow, Ewan Maidment, Ewan Morrison Yung, Ewan Tait, Faaizah Chishty, Fabbiha Ashad, Faduma Mohamud, Fahad Khan, Fahamida Heba, Fahim Valera, Fahmida J Miah, Faid Khopekar, Faisal Ali, Faisal Alshukri, Faisal hanif, Faisal Rehman, Faith Lee, Faith Odede, Faith Sara Luke, Faith Solanke, Faith Utulu, Faith Waidyaratna, Faiz Masood, Faizaan Hussain, Faizan shah, Falaq Syed Raheel, Fanika Saeeda Awan, Faqih Laulloo, Farah Bachar, Farah El-Sharnouby, Farah Hasan, Farhaana Surti, Farhan Miah, Farhana Mostafiz, Farhat Bibi, Fariha Rehman, Faris Iftikhar Hussain, Faris Raza, Fatema Hassanali, Fatemeh Kalabi, Fathima Islam, Fatiah Mustapha, Fatiah Mustapha, Fatima Babar, Fatima Camp, Fatima Elshams, Fatima Khan, Fatima Nasidi Mohammed, Fatima Saleh Shehu, Fatimah Naeem, Fatimah Riaz, Fatmah Hasan, Fatmata Mahdi, Faustina Nwodo, Fawwaz Alomiri, Faye Chen, Fearghal McGovern, Febi, Federica Poli, Federico Pastor-Franco, Felicite Mukeshimana, Felicity Greenfield, Felix Flechtner, Felix von Cossel, Felix Von Spreckelsen, Fellah Khan, Felyx Wong, Ferdinand Boucher, Fern Pattinson, Fernanda C Widdowson, Ffion Byrne, Ffion Hargood, Fiina Närhi, Filzah Hanis Binte Osman, Finlay Powell-Jones, Finley Dyer, Finn Galloway, Finn Jonathan Norris, Finn severwright, Finola Mankel, Fiona Abigail Joseph, Fiona Bell, Fiona Chegwidden, Fiona Craigen, Fiona Farquharson, Fiona Harris, Fiona Howells, Fiona Kehinde, Fiona Nhi Dang, Fionán McBride, Fionnuala McGrade, Firas Charfare, Firas Nasr, Firdusi Khan, Flana Barreto, Flora Chan, Flora Johnson, Flora Williams Burton, Florence Caslake Holding, Florence Kinder, Florence Sumner, Florence Williams, Fouzhan Orangian, Fran Crowe, Franca Still, Frances Arnott, Francesca Bilton, Francesca Birch, Francesca Blest, Francesca Bowles, Francesca Florida-Chapman, Francesca Hopkins, Francesca Law, Francesca Neale, Francesca Pang, Francesca Rushton, Francesca Thornton-Wood, Francesca Watson, Francesca Wright, Francesco Edwards, Francis Aggrey, Frank Thornton-Wood, Frankie Fan, Franklin Marshall-Collins, Fraser Gold, Fraser Kenny, Fraser Ritchie, Freda Ngu, Freddie Allum, Freddie Ayliffe, Frederick Shi-Hong Yeo, Frederick Tilby-Jones, Frederick Yeo, Frederika St John, Fredrika Asenius, Freya Alexandra Braddon, Freya Crispin, Freya Fagan-Bird, Freya Palmer, Fui Lin Wong, Fumi Olaifa, Furqaan Kaji, G Revolta, Gabriel Bellamy Plaice, Gabriella Birley, Gabriella Breese, Gabriella Weisz, Gabriella Williamson, Gabrielle Day, Gabrielle Moe, Gagandeep Singh Sachdeva, Gahyour Abbas syed, Gareth Davies, Gareth Hutchinson, Gareth Williams, Garikai Kungwengwe, Gar-Ling Simcock, Gaurav Binod Menon, Gayatri Kulkarni, Gemma Black, Gemma Harley, Gemma Lindsey, Gemma Penman, Gemma Turner, Gemma Whyatt, Genevieve Easingwood, Genevieve Lawrence, Genevieve Perrins, George Davies, George Dyke, George E G Hunt, George Esworthy, George Freer, George Garratt, George Hadjiyiannakis, George Harry, George Higginbotham, George Hoult, George Kulangara, George McCarron, George Mundy-Baird, George Oliver Cox, George Phenix, George Seligmann, George Shaw, George Stannard, George Taylor-Walker, Georgia Ashley, Georgia Chard, Georgia Gadsby, Georgia Gilbert, Georgia Hainsworth, Georgia Hogg, Georgia Ineson, Georgia Linas, Georgia Morgan, Georgia Parry, Georgia Robinson, Georgia Rowley, Georgia savvides, Georgia Simpson, Georgia Thomason, Georgia Williams, Georgiana Butcher, Georgina Blake, Georgina Cooper, Georgina Covell, Georgina Dalton, Georgina Ewer, Georgina Holme, Georgina Jackson, Georgina K O'Reilly, Georgina Kellie, Georgina Lewis, Georgina Lithgow, Georgina Louise King, Georgina Mae Kelly, Georgina Mae Kelly, Georgina St Pier, Geraldine O'Brien, Gethin Rhys Thomas, Ghazal Najafi, Ghazia Ahmed, Ghufran Al Sayed, Gianfranco Messina, Gianluca Bertone, Gianluca Fiorentini, Gianni Nero, Gifty Brown, Gilbert Antona, Gimeshi Jayalath, Gladily Saji, Glen J Munday, Gloria Matindi, Gofaone Mogapi, Golasa Sheikh Akbari, Gowri Lekshmi Sujathan, Gowri Pradeep, Grace Afolalu, Grace Cato, Grace Farnworth, Grace Hodgson, Grace Lee, Grace Lee, Grace Leyland, Grace Loy Ming Hooi, Grace Perring, Grace Pymm, Grace Rahmatallah, Grace Richmond, Grace Rotheram, Grace Stott, Grace Tan Chin Lay, Grace Whyman, Grace Williams, Gregor Abercromby, Gregory Knowles, Greta Safoncik, Guan Hui Tricia Lim, Guan-An Chen, Guleed Mohamed, Gunalini

Gunendran, Gurdeep Bagary, Gurdip Uppal, Gurjivan Singh, Gurkiran Bhogal, Gurleen Singh, Gurnoor Nagi, Gurpreet Gill, Gurpreet Kaur Atwal, Gurpreet Kaur Jandu, Gursharun Hayer, Guy Dunbavand, Gwen Cheong, Gwenllian Roberts, Gwilym Geraint Preest, H Amin, H Arun Khan, H Evans, H Sturgeon, Hadeeqa Jabeen Mahmood, Hadis Reyhani, Hafsa Duale, Hafsa Khan, Hafsah Khan-Cheema, Haider Ali, Haider Khan, Haider Merchant, Hajer, Hakam M. Jabouri, Halbast Awarah, Haleema S Adil, Haleema Siddique, Halima Farah Mohamud, Halima Koroma, Halima Naeem, Halima Warraich, Halimah Khalil, Halimah Yasin, Hamaas Hassan, Hamima Uddin, Hamish Kelman, Hamish Matthews, Hammaad Ali Khan-Afridi, Hammaad Khalid, Hammad, Hamza, Hamza Hussain, Hamza Shahbaz, Hamza Umar, Hamzah Hanif, Hamzah Shahid, Han Wei Xuan Oscar, Hana Fatima Panakkat, Hana Javed, Hana Yusuf, Hanaa Ali, Hanaa Amaidia, Hanaa Nawaar Mughal, Hanan Hassan, Hania Karamat, Hania Shahzad Qureshi, Hania Sohawon, Haniya Sattar, Hanna Brant, Hanna Whydle, Hannah, Hannah Adams, Hannah Ballheimer, Hannah Beattie, Hannah Breakwell, Hannah Brotherwood, Hannah Brown, Hannah Century, Hannah Cooney, Hannah Craig, Hannah Dalt, Hannah Dial, Hannah Duthie, Hannah Emerson, Hannah Freeman, Hannah Ghani, Hannah Graves, Hannah Gray, Hannah Greensmith, Hannah Grimes, Hannah Gyekye-Mensah, Hannah Holmes, Hannah Hunter, Hannah Jemphrey, Hannah Jones, Hannah Kwuo, Hannah L Rossiter, Hannah Lally, Hannah Lapin, Hannah Lawlor, Hannah Logan, Hannah Morrison, Hannah Neill, Hannah Noone, Hannah Prill, Hannah Punter, Hannah Redpath, Hannah Rowley, Hannah Royle, Hannah Rudd, Hannah Scholes, Hannah Sutton, Hannah Symcox, Hannah Victoria Della-Porta, Hannah Waheed, Hannah Waite, Hannah Willis, Hannah Woodman, Hansa Iqbal, Hansini Abeysekera, Hao Ding, Hao Gao, Haqeeqat Singh Gurm, Hardeep Gahir, Hari Bhachoo, Hari kallat, Harin Wijayathunga, Harini Suren, Haris Khan, Harjan Singh Sandhar, Harjeevan Kang, Harjevanjit Khera, Harkirt Sandhu, Harmeen Kaur Jagpal, Harnoor Khroud, Harpal Patel, Harriet Barton, Harriet Conley, Harriet Flashman, Harriet Francis, Harriet Howells, Harriet Jones, Harriet Lomholt-Welch, Harriet Louden, Harriet Morris, Harriet Ogle, Harriet Potter, Harriet Shaw, Harriet Wood, Harris Ali, Harriwin Selvarajoo, Harroop Bola, Harry Barker, Harry Brown, Harry Dudson, Harry Gething, Harry Griffiths, Harry Kyriacou, Harry Pavlou, Harry Rosen, Harry Sandford, Harry Searle, Harry Senior, Harry Smith, Harry Warren, Harry Winter-Taylor, Harsh Agarwal, Harsha Sinha, Harshaka Samarasinghe, Harshil Halai, Harun Ali, Harveer Narula, Harvey Dickerson, Harvey Stevenson, Harvey Xiang, Hasaan Aziz, Hasaan Khan, Hasan kamil, Hasan Murad, Hasan Zeb, Haseeb Ahmed, Haseeb Iqbal, Haseena Wazir, Hashaam Choudhry, Hashvi Shah, Hasini Chandrasekera, Hassan Jalil, Hassan Mohammad, Hassan Mustafa, Hasti Tarzban, Hateem Rafeeque, Hathsu Dissanayake, Haves Havishma Sreedharala, Hawawu Muazu, Haya AlAmeri, Haya Nasser, Hayley Boyom, Hayley johnson, Hayley Unwin, Hayley White, Hayley Williams, Hazel Sanghvi, Heather Bragg, Heather Craig, Heather Holden, Heather Keys, Heather Lawson, Heba S H A A Altuwaijri, Heer Trivedi, Heewon Yoon, Hei Chun Wong, Hei Man Priscilla Chan, Hei Yi Vivian Pak, Hei Yu Lam, Helen Bailey, Helen Frank, Helen Gaynor, Helen Ng, Helen Rimell, Helen Thompson, Helena Davies, Helena Ironton, Helena Martin, Helena Martin, Helena Thompson, Helena Wiles, Helene Pans, Heli Baho, Hema Rubinii Mohan Raj, Heng Chun Wong, Henley Crodden, Henna Hussain, Henry Cox, Henry Graham-Rack, Henry Lloyd, Henry Maudslay, Henry Phillips, Henry Somers, Heo Mun Ho, Hepsi Xavier, Herkiran Kaur Kambo, Hermann Jacobs, HERNG SHERN TAN, Hessa Akram, Hester Lacey, Hester Lloyd-Cox, Hester Rose Garratt, Hettie Stevens, Hetty Breed, Hetty Niblett, Hiba Al-B, Hiba Alzein, Hiba Lusta, Hibah Mirza, Hibatallah Altaher, Hikari Shida, Hilary Williams, Himaja Sakhamuri, Himal Biswas, Hira Arab, Hira Fatima Hassan, Hiral Billimoria, Hishaam Yunas, Hithin Noble, Hiu Kwan Fiona Fung, Hiu Kwong, Ho Lun Chong, Ho Yau Chloe Vun, Ho Yip Michael Lai, Hoi-Yee Wong, Hollie Chadwick, Holly Beard, Holly Butler, Holly Creighton, Holly Elizabeth Wilson, Holly Garnett, Holly Lamden, Holly Nicole Hellawell, Holly Raison, Holly Thompson, Holly Trippe, Holly Wilkins, Hong yu Ngu, Honour Okoli, Hoozaifa Master, Hope Chow, Howraa Yahya, Hozaifa Ahmed Sahi, Htet Oo, Hugh Harris, Hugo Duncan-Duggal, Hugo Ferreira, Hugo Labat, Hugo Walford, Hui Mei Wong, Hui Ping Lee, Hui Sheng Ke, Hui Yun Ng, Huili James Chong, Huma Hafeez, Huma Sailani, Humaira Maka, Humayra Piranie, Hummul-Bayyinat Mohammed-Hadi, Humza Khan, Huria Metezai, Hurooul Aain, Husain Ahmed, Husna Shinwari, Husnain Shah, Hussain Nadeem, Hussein Said, Huw Thomas, Hyerim Kwon, Hyun Park, Hyun Sung Moon, I.kalaitzis, Ïa Robert-Montaner, Iain Logan, Iakovos Theodoulou, Ian Blackburn, Ian Chung Ka Hien, Ian Gordon, Ibraheem Khalil, Ibrahim Alam, Ibrahim Ansar, Ibukunoluwa Oni, Idil Mohamed, Iestyn Fenn, Iffah Aminah Jawaheer, Iffath Javeed, Iheanyichukwu Carlton Chukwujindu, Iida Lohi, Ijaz Ahmed Choudhury, Ikra Mahmood, Ilaria Ferroni, Iman Anis, Iman Ansar, Imana Khalique, Imani Looby, Immaculata Aloysius, Immanuelle Nyamali, Imogen Cayley, Imogen Kurek-smith, Imogen Ludman, Imogen MacDermott, Imogen Parker, Imogen Robb, Imogen Young, Imran Abdullah Ashraf, Imran Karim Janmohamed, Inas Alsuhaibani, Inayat Khan, Inderpal Rajwancee, India Corrin, India Daniels, India Dickinson, India Hamilton, India Mayhook-Walker, India Rose

Barrons, India Smart, Inês Correia, Inés Murray, Ingrid Lee, Inioluwa Eniola, Innocent Ogunmwonyi, Inshara Saiyed, Insiya Alibhai, Insiya Alibhai, Intesar Nur, Intisar Moalin, Ioannis Theocharopoulos, Iona, Iona Cleer, Iona Lindsay, Iona Tatham, Ipsita Sarkar, Iqraa Jalil, Iqraa Khan, Irfaan Ahmad, Irtiqa Ahmed, Is an Ramli, Isa Hassan, Isaac Wahnon, Isaac Woods, Isabel Allison, Isabel Askey, Isabel B Broadmeadow, Isabel Boden, Isabel Bunola-Hadfield, Isabel Butf, Isabel Carter, Isabel Leach, Isabel Mason, Isabel Morgan, Isabel Munden, Isabel Rafferty, Isabel Randall, Isabel Raynaud, Isabel Rimmer, Isabella Atherley, Isabella Davies, Isabella Munford, Isabella Reid, Isabella Talbot, Isabella Taverner, Isabelle Bishop, Isabelle Butcher, Isabelle J.M. Williams, Isabelle Legood, Isabelle Mayne, Isabelle Murray, Isabelle Schiff, Isatu Bah, Isha Najeem, Ishaan Singh, Ishani Sharma, Ishika Bansal, Ishika Prachee, Ishita Bobba, Ishita Datta, Isidora Staikidou, Isla Harper, Ismah Aslam, Isobel Baxter, Isobel Bremner, Isobel Daggitt, Isobel Davis, Isobel Dawbarn, Isobel Hall, Isobel Jones, Isobel Meaton, Isra Ahmed, Isra Al Affani, Isra Jalaly, Israa Ali, Italia-Rosa Leech, Iulia Bock, Ivana Homerova, Ivie Itua, Ivy Kar Yin Ho, Izy Millward, Izzy, J Britton, J Emily McClintock, J Jones, J Saji, J Sohal, Jack Barrington, Jack Bennett, Jack Bradbury, Jack Cope, Jack Coumbe, Jack Devin, Jack Gallagher, Jack Hao, Jack Hogg, Jack Lewis, Jack London, Jack Maloney, Jack Matthewman, Jack McLachlan, Jack Merritt, Jack Miller, Jack P, Jack Roddy, Jack Starkie, Jack Tighe, Jack Tun Loh, Jack Van-Loo, Jack Wellington, Jacob Clark, Jacob Clayton, Jacob Feathers, Jacob Piechota, Jacob Spencer, Jacob Virchis, Jacob Whatmore, Jacob Williams, Jacqualyn Walsh-House, Jacqueline Lee, Jacqueline Olaifa, Jacques Laverty, Jad Traboulssi, Jade Hanley, Jade Kiersten, Jade Lopes, Jade Sangha, Jagvi Patel, Jai Chopra, Jaina Narendra Surti, Jake Baker, Jake Bickerton, Jake Cawley, Jakevir Shoker, Jakir Ahmed, Jakob Ko, Jakub Wysowski, Jamal Khudr, Jameela Bahar, Jameela Nagri, Jameela Sheikh, James Adams, James Barry, James Bell, James Celaire, James Chambers, James Clarke, James Downes, James Drake, James Flynn, James Gerard Irwin, James Henderson, James Hobson, James Hong, James Inchley, James Irwin, James Kerrison, James M Shipton, James Mason, James McHale, James McLaren, James Morbin, James Murdoch, James Newton, James O'Brien, James Okeleke, James Peaty, James Peel, James Prentice, James Pyke, James Reilly, James Sheppard, James Short, James Slaven, James Smith, James Smith, James Stock, James Stret, James Suddaby, James T Scott, James Taylor, James Tooke, James Weller, James Wing, James Young, James Young, Jami Hanif, Jamie Beverstock, Jamie Collins, Jamie McGinn, Jamie McGuigan, Jamie Monchief, Jamie Sanghera, Jamie Smith, Jamie Thompson, Jan Drmota, Jana Heinz, Janahan Suthakar, Jancis Tung, Jane Fan, Jane Poh Yi Jen, Janet Tsz Yau Wong, Janey Gregory, Janice Chow Chun Wing, Janie Bamforth, Janine Dacanay, Jannah Holmes, Jansher Petrache Mahmood, Janthula Ranchagoda, Japsimar Kaur, Jared Raistrick, Jaroslaw Liszka, Jasdeep, Jasdeep Dhaliwal, Jasin Philip Kanacherril, Jasleen Gabrie, Jasmin Kaur Rai, Jasmine Chen, Jasmine Coulthurst, Jasmine Foyzul, Jasmine Gupta, Jasmine Holder, Jasmine Hulme Kennh, Jasmine Koe Sze Ern, Jasmine Limbu, Jasmine Moroney, Jasmine Pattarukuzhyil Jose, Jasmine Simon, Jasmine Sutaria, Jasmine Virk, Jasmine Yap, Jason Armstrong, Jason Nicoletti, Jaspinder Sanghera, Jastine Rivera, Jatin Chopra, Javeria Tariq, Jawad Ahmad, Jay Anil Patel, Jay Panchal, Jay Shah, Jaya Upadhyay, Jayanth Sai Ramesh, Jayden Gittens, Jayden Patel, Jayesh Patel, Jayne Fenton, Jayne McGoldrick, Jean Claude Doukrou, Jeanette Olorunniwo, Jean-Luc Duval, Jeel Shukla, Jeevan Francis, Jeeveththaa Thirugnanasambanthar, Jeffrey Gan, Jeffrey Leung, Jemi Maliyil, Jemiliat Isadora Otun, Jemima Jones, Jemima Payne, Jemimah Arolasafe, Jemini Patel, Jemma Cook, Jemma Keefe, Jemma Scattergood, Jenardan Sellathurai, Jenna Blackaby, Jenna Fielding, Jenni Mair, Jennie Parker, Jennifer Anne Mooney, Jennifer Campbell, Jennifer Frazer, Jennifer Knight, Jennifer LaBranche, Jennifer Luu, Jennifer McCann, Jennifer McLean, Jennifer Mcloughlin, Jennifer Pewsey, Jennifer Ptt, Jennifer Tainsh, Jennifer Warwick, Jennifer Whitby, Jenny Chia, Jenny Hubball, Jenny Quang, Jenny Stephenson, Jenny Y Wang, Jeremy Chan, Jeremy Cheong, Jeremy Easow Maducolil, Jeremy Maganji, Jeremy Ng Chieng Hin, Jeremy Rison, Jeremy Samuel, Jeremy Thomas, Jeremy Warner, Jerica Kuah, Jerome Akshay Randle-Rai, Jess Appleby, Jessamyn Heung-Ying Lee, Jesse Edward Anthony, Jessica Arnold, Jessica Bales, Jessica Bialan, Jessica Boyle, Jessica Catchpole, Jessica Davies, Jessica Donaghue, Jessica Dudley, Jessica Duffy, Jessica Flint, Jessica Gladwell, Jessica Harbottle, Jessica Harris, Jessica Hill, Jessica Howes, Jessica Lam, Jessica Lane, Jessica Lily, Jessica Lythgoe, Jessica Man, Jessica Neden, Jessica Nicholas, Jessica Perrett, Jessica Pieri, Jessica Riley Green, Jessica Sinclair, Jessica Singleton, Jessica Taylor, Jessica Tidley, Jessica Upadhyay, Jessica Walker, Jessica Westwood, Jessica Wheeler, Jessica Whitney, Jessica Willis, Jessie johnson, Jesus Perdomo Lampignano, Jesvin Tom Sunny, Jetmond Ma, Jewel Joseph, Jhia Teh, Jia Sen Diong, Jiakun Yu, Jiang An Lim, Jiawen Dong, Jie Fei Lau, Jihad Salim Al Kamyani, Jilhad Ali, Jill Fraser, Jill-Charlotte Kasule, Jimena Abeledo Vilarino, Jinkun He, Jishel Mathews, Jo Haxworth, Joanna Amaranthi Somasundaram, Joanna Boxall, Joanna Livesey, Joanna Low Yi Hui, Joanna Mitchell, Joanna Park, Joanna Thorpe, Joanna Ting, Joanna Wolska, Joanne Buckingham, Joanne Curry, Joanne McCabe, Joanne Tan, Jocelyn Luveta, Jocelyn Sheung Hang

Mak, Jodi Hebson, Jodie Taylor, Joe Frost, Joe Kirkpatrick, Joe Stevenson, Joel Hunter, Joel Lee Zher Jong, Joel Matthews, Joel Thomas John, Joelle Johns, Johan Jandel, Johannah Harris, Johannes Yu, John, John Ayoola, John Creber, John Daveney, John Ferrick, John Humm, John Saganty, John Towers, Joji Verghese, Jomcy John, Jonathan, Jonathan Barton, Jonathan Brend, Jonathan Bruce, Jonathan Chan, Jonathan Chua, Jonathan Creamer, Jonathan Eaton-Hart, Jonathan Farmer, Jonathan Hansford, Jonathan Jackson, Jonathan James Musgrove, Jonathan James Smith, Jonathan K M Tobin, Jonathan McGrath, Jonathan McIntosh, Jonathan Old, Jonathan Wan, Jonathan William Sheridan, Jonny lorimer, Jonny Varma, Jordan Chow, Jordan Delong, Jordan Elliott, Jordan Foley, Jordan Halley-Murray, Jordan Hargreaves, Jordan Khoo Yew Hock, Jordan Lee Russell, Jordan Tomos Lin, Jordan Tudor, Jordon Kong, Joseph Anthony Cross, Joseph Atley, Joseph Chan, Joseph Charles Dean, Joseph Cohen, Joseph Davies, Joseph Hamlyn, Joseph Harker, Joseph Holdsworth, Joseph Iley, Joseph Karran, Joseph Kirkbright, Joseph Lake, Joseph Louis Jervis Froud, Joseph Macpherson, Joseph Norvill, Joseph Reynolds, Joseph Rona, Joseph Williamson, Josephine Chen, Josephine Isaac, Josephine Isaacs, Josephine Mollier, Josephine Norre Hogh, Josephine Rahman, Josh Beach, Josh Brown, Josh Drinkwater, Josh Matthews, Josh Pettit, Josh Pritesh Kotecha, Josh Sinclair, Joshua Chambers, Joshua Edwards, Joshua Galloway, Joshua Garg, Joshua George John, Joshua Howard-Taylor, Joshua Killilea, Joshua Li Saw Hee, Joshua Packiam Gray Jimmy Edwin Palmer, Joshua Reeves, Joshua Shaw, Joshua Sterriker, Joshua Sturgeon, Joshua Wong rui yen, Joss Digweed, Jowad F, Joy Bannister, Joyita Saha, Judith Scott, Judith Scott, Judy Tabbakh, Juin Low, Julen Bilbao, Julia Borowicz, Julia Clarke, Julia Craggs, Julia Manning, Julia Morris, Julian Aquilina, Julie Soo Fei Gan, Juliet Raphael, Juliusz O. Rogowski, Jun Chuen Hui, Junaid Ahmed Shaikh, Junaid Naveed, Juned Ahmed, Junkai Zhu, Jun-Li Tham, Justin Chow, Justin Colleen Co, Justina Cheh Juan Tai, Juwairryah Butt, K E Bailey, K. Thejasvin, Ka Ching Genie Wu, Ka Hin Karen Mak, Ka Wing Eric Wong, Ka Yu Jasmine Tang, Ka Yuet Au-Yeung, Kaa-Yung Ng, Kabyar Cho, Kaela Brook, Kageena Selvarajah, Kah Keen Kong, Kai Yin Ang, Kaif Mahmood, Kaijanee Kugavarathan, Kaitlan Hadfield, Kajal Aubeeluck, Kajani Subhaskaran, Kalina Czyzykowska, Kalp Patel, Kalyani Shinkar, Kalyisah Suada, Kam Hei Michelle Chan, Kamal Shah, Kamalakannan Arunachalam, Kamarl Rauf, Kamil Chaudhry, Kamna Karan, Kamran Basharat, Kanokpon Tanthiptham, Kapil Agarwal, Kar Yan Chong, Kar Yan Yip, Kar Yen Phoong, Karan Daga, Karan sagoo, Karanjeet Sagoo, Karanjot Chhatwal, Kareem Omran, Kareem Pabani, Karen Allen, Karina Ramdenee, Karinn Farquharson, Karis Harbertson, Karishma Hurry, Karishma Nursiah, Karishma Rajput, karishma viramgama, Karisma Sharma, Karl Stones, Karlo Jagic, Karmen Sow, Karol Basta, Karola Meunier, Karolina Futera, Karthik Kurian, Kartik Kumar, Kartikeya Bhardwaj, Kasey Thompson, Kashaf Noor-ul-ain, Kashmira Jeeva, Kasia kaluza-gilbert, Kasim Aslam, Kasim Jeena, Kaso Kareem, Katarina Kentosova, Katarzyna Minta, Kate Boardman, Kate Dobbs, Kate Francis, Kate Higgins, Kate Jones, Kate Mackay, Kate Plater, Kate Price, Kate Richardson, Kate Tindaea Naylor, Kathan Desai, Katharine Hood, Katherine Alker, Katherine Argent, Katherine Elcock, Katherine Hodge, Katherine Howick, Katherine Jackson, Katherine Jurdon, Katherine Laura Graham, Katherine Memory, Katherine O'Byrne, Katherine Rennie, Katherine Spofforth, Katherine Terence, Katherine Wallis, Katherine Wise, Kathleen Ewins, Kathleen McCaffery, Kathryn Ferguson, Kathryn Gaskell, Kathryn McGrotty, Kathryn Mitchell, Kathryn Routledge, Katie Allen, Katie Ash, Katie Birt, Katie Bland, Katie Blaylock, Katie Bloor, Katie Cheung, Katie Elizabeth Joyce, Katie Fyffe, Katie Graham, Katie Gunn, Katie James, Katie Kirby, Katie Kwan, Katie Lennon, Katie Marriott, Katie McCullough, Katie Overend, Katie Sharman, Katie Stevenson, Katie Whyte, Katie Wilkinson, Katija Hunt, Katrina Paton, Katryn Lara Schlich-Davies, Katy Crisp, Katy L. Caverly, Katy Whitley, Katya Qiao, Kausar Ghafuri, Kaval Patel, Kavin Kugan, Kavita Prashar, Kavita Shergill, Kavitha Vijayakumar, Kavyesh Vivek, Kawser Ahmed, Kaycee Child, Kayleigh MacNay, Kayleigh Ng, Kayleigh Nicholson, Kayley Griffin, Ke Xin Ong, Kee Ling Ling, Keeran Sivalingam, Keerthi Muthukumar, Kehaan Akram, Keira Bralsford, Keira Nakazato, Keiran Hylton, Keith Sai Kit Leung, Kellie Stevens, Kelly Earnshaw, Kelly Ka Yee Chu, Kelly Lee, Kelly Ogilvie, Kelsey Licudi, Kelvin Lam Thuon Mine, Kemi Ahmed, Kenal Patel, Keneth Yomal Kodituwakku, Keng Siang Lee, Kerin Borer, Kerry Canning, Kerry Leyton, Kerry Wales, Kesri Gajadhar, Kestra Dawson, Kevin Ferrao, Kevin Kodituwakku, Kevin koshy, Keya Pindolia, Kezia King, KH, Khadija Hossain, Khadija Meghrawi, Khadijah Ginwalla, Khaing Thu Thu, Khairul Ain Binti Kamarudin, Khalid Bukhashem, Khanyisa Hoshe, Kheelan, Kheerthiharan Saravanan, Khizar Abbas Khoja, Khui Chiang Wee, Khushboo Khatri, Kian Patel, Kien Hang, Kieran Dey, Kieran Howarth, Kieran Loughran, Kilanalei Bishop, Kim Alipio, Kimberley Phiri, Kimberly Punwani, Kimiya Bagheri, Kinan Wihba, Kira Sneddon, Kiran Bhandal, Kiran Bhangu, Kiran Kaur Sanghera, Kiran Khamb, Kiran Nathan, Kiran Pillai, Kirandeep Kaur, Kirsten Athawes, Kirsten Davis, Kirsten Goves, Kirsten Hunter, Kirstie Wright, Kirstin Sooriah, Kirsty Cotterill-Stavers, Kirsty Fraser, Kirsty Luo-Yng Tay, Kirsty Small, Kirthika Mohanathass, Kiruthicka Selvakumar, Kishan Patel, Kishan Vadher, Kitty Towl, Klaudia Agnieszka Figa, Klaudio

Bisha, KNB, Kofi Cox, Komal Doal, KONG YONG FAI, Krish Himanshu Shah, Krishan Haria, Krishan Nandapalan, Krishna Mehta, Krishna Savadia, Kristi Gremels, Kristin Leigh Bryce, Kriti Yadav, Kritika Yadav, Krupa Thomas, Krzysztof Malinowski, Ku Ji Yen, Kubra Shah, Kuma Nkuo, Kumarasamy Sivanu, Kumayl Bhalloo, Kunal Namjoshi, Kunal Pathak, Kunam Mukan, Kush Sharma, Kushagra Bisht, Kushal Basnyat, Kushal Manon, Kusy Suleiman, Kwarteng Opoku Sarfo, Kyi Aye, Kyla michie, Kyriacos Thoma, Kyrie Wheeler, Laareb Gul Baloch, Laetitia Jervis, Laiba Butt, Laiba Rahman, Laila Khan, Laju Gurung, Lakhan SVA Ajmeria, Lan Ge, Lap Kan Derek Yu, Lara Fossen, Lara Green, Lara Nassar, Lara Othman, Lara Yorke, Laraib Gul Khan, Larissa Boyd, Lars Hanssen, Laura, Laura Appleton, Laura Butters, Laura Chapman, Laura Cowlbeck, Laura Cunningham, Laura Giles, Laura Gilliland, Laura Hansell, Laura Hudson, Laura Inglis, Laura K Taylor, Laura Knowles, Laura Lazzari, Laura Macpherson, Laura Maynard, Laura McCafferty, Laura McCowan, Laura Miller, Laura Owler, Laura Robertson, Laura Shilston, Laura Smith, Laura Sofia Vescance Caroprese, Laura Stark, Laura Tincknell, Laureen Marie, Lauren A Glover, Lauren Bateman, Lauren Bell, Lauren Bennett, Lauren Chandler, Lauren Cunningham, Lauren dixie, Lauren Ferguson, Lauren Frame, Lauren Franklin, Lauren Gurr, Lauren Hargreaves, Lauren Hartley, Lauren Hicks, Lauren Horsburgh, Lauren Ives, Lauren James, Lauren Laird, Lauren Lee, Lauren Lee, Lauren McMullan, Lauren O'Connor, Lauren Pererson, Lauren Sells, Lauren Simmonds, Lauren Simmons, Lauren Tziganie Chandler, Lauren Wilson, Laurence Zammit, Laurensius Mainsiouw, Lavesh Mirpuri, Lavinia Marie Dillon Maes, Lawrence Hin Hai Lee, Laxsan Karunanithy, Leah Clarke, Leah Fagan, Leah Greenman, Leah Linda Brooks, Leah Mangham, Leah Sihan, Leanda Akuoko, Leanne Kade, Leanne Smith, Lee Ballard, Lee Byrne, Lee Mun Leng, Leeann Yao, Leena Ahmed, Leena Khelifa, Leena Patel, Leigh Cui, Leigh-Anne Brace, Leila Ellis, Leila White, Lelyn Osei Atiemo, Lena Smith, Leo D. Baxendale-Smith, Leo G, Leo Stallard, Leonardo Sherif, Leonora Bartlett, Leslie Chiyan Cheung, Lewis Allan, Lewis Bonsell, Lewis Greenan-Barrett, Lewis Hancock, Lewis McColm, Lewis Mitchell, Lewis Patrick, Lewis Petrie, Lewis Swan, Li San Tang, Li Wai Kei, Tommy, Lia Hart, Lia Millanaise Jones, Liam Adams, Liam Mangaru, Liam McCreadie, Liam Richards, Liam Snook, Liam Thompson, Liam Ward, Liang Zhi Wong, Lianne Boyle, Liba Aysha, Libby Bowles, Libby Gooda, Libby Griffiths, Lien Salcedo, Liew Yong Yie, Lije Johnson, Lilian Symons, Lilly Yarnell-Gafney, Lily Farakish, Lily Marrable, Lily Maynard, Lily Mills, Lily Rouhi, Lim Lu Yen, Lim Ren Jye, Lim Shan Ming, Lim Zen, Lina Al Omari, Lina Chevalier, Lina Futsum Ogbagabriel, Lina Nottrodt, Lindsey Hodges, Linnet Mensuoh, Liron Ingleby, Lispeth Abraham, Livia Cheung, Livia Walsh, Liz Baxter, Liz Wilson, Lizzie McGladdery, Llew Lloyd, Lloyd Schanzer, lochlan fisher, Loh Wan Ling, Loïc Hayois, Lola Arowoshola, Loliya Tyger, Long Ki Cheung, LOO JIE QI, Lorcan Elliott, Lorcan McMullan, Lorcan Moore, Lorelle Mault, Loren Wilkins, Lorenzo Mandolfo, Lorna Blackmord, Lorna Galbraith, Lorna J Haddon-McMillan, Lorraine Pinto, Lorraine Spotten, Lottie Jephson, Loubna Kraria, Louis Claxton, Louis Goff, Louis Lowry, Louis Silvano, Louisa Child, Louisa Sowah Quarshie, Louise Chapman, Louise E Young, Louise Edwards, Louise Jayne Brown, Louise Powell, Louise queipo, Louise Sanderson, Louise Walker, Loujin Al-Ani, Lowri Hughes Thomas, Lubna Jameel, Luc Worthington, Luca Galvani, Luca Milford, Luchiana Fernando, Luciana Nicole Crowther, Lucie Collinge, Lucie Robertson, Lucie Webber, Lucija Kovacic, Lucinda Knight, Luckshi Jegatheeswaran, Lucy Acheson, Lucy Amanya, Lucy Atherton, Lucy Bidwell, Lucy Deller, Lucy Dundas, Lucy Elliott, Lucy Hayes, Lucy Helen Belk, Lucy Jane Westwood, Lucy McCann, Lucy McGarvey, Lucy Moors, Lucy Osborne, Lucy Peters, Lucy Pulle, Lucy Rabuszko, Lucy Reason, Lucy Ryans, Lucy Sidey, Lucy Soudain, Lucy Stones, Lucy Taylor, Lucy Violet Allison, Lucy Wallace, Lucy whittaker, Lucy Wilson, Lucy-Ann O'Kane, Lueh Chien, Luisa Hofmaier, Luke Alexander, Luke Auterson, Luke Christopher Taylor, Luke Holt, Luke Murphy, Luke Robinson, Luke Sherry, Luke Thompson, Luke Zhu, Lukon Miah, Lupei Cai, Luqman Al Hakim Tenang, Lusyan Dayalan, Luthfun Nessa, Lydia Bancroft, Lydia Cullen, Lydia Hancock, Lydia Lee, Lydia Roberts, Lydia Shackshaft, Lydia Stratford, Lydia Thom, Lydia Wells, Lyndon Lo, Lynette Loi, Lynne Thornton, Lynsey Harrower, M Gupta, M Makeen Baroudi, M. Corotana, M.Ud, Maarij Mirza, Maariyah Mahmood, Maarja-Liis Ferry, Maatla Tshimologo, Maciej Kwasigroch, Maciej Pysklo, Maddie Cobbin, Maddy Wood, Maddy Young, Madelaine Dare, Madelaine Miller, Madeleine Durkan, Madeleine Foster, Madeleine Mills, Madeleine Oliver, Madeleine Upham, Madeline Izza, Madeline Louisa Smith, Madeline ward, Madeline Witcomb, Madelyn Oliver, Madhavan Hennessey, Madhavi Berks, Madison Fairey, Mae Elmes, Mae Wightman, Magd Nojoum, Magnus Okoh, Maha Ejaz, Mahad Mohamud, Maharsh Pandya, Mahaveer Sangha, Mahbubur Rahman, Mahdia Obeda, Mahdiyyah Osman, Maheen Ali, Maheen Shukir, Maher El Sahar, Mahi Islam, Mahima Pillai, Mahir Bhoora, Mahira Zahid, Mahjabin Kowser Chowdhury, Mahmood AlAbdulrazzaq, Mahmood Yousuf, Mahnoor Javed, Mai Alkari, Mai Baalbaki, Maia Brown, Maia Kaur, Maia Osborne-Grinter, Maiar Elhariry, Mairi Weir, Maisy Lopez Piggott, Makinah Haq, Makishaa Nanthakumar, Mäla Jheinga, Malachi Saccomani, Malaika Gupta, Malak El-Askary, Malak Mohamed, Malavan Sreemurthy, Malayka Jaffary, Maleehah Yusufzai, Maliha Khan, Mallaika

Viswanath, Malvika Subramaniam, Malyka Cheema, Mami Samejima, Manaal Ebrahimkutty Anwar, Manahil Tareen, Manas Soni, Manasi Mahesh Shirke, Manavi Purohit, Mandy Hui Qi Tan, Maneeha Naveed, Maneesha Aruketty, Manini Bharadwaj, Manisha Mahadeva, Manisha Santosh, Manishaa Vairavan, Manmohan Sandhu, Mannat Rana, Manu Sidhu, Maram Nabahin, Marcin P Kowalczyk, Marco David Bokobza de la Rosa, Marcus Boyd, Marcus Carslaw, Marcus Chong, Marcus Graham, Marcus Kyriakides, Marcus Rathbone, Mareen Zachariah, Margaret Andrews, Margaret Maxim, Margareta Lamackova, Marguerite O'Riordan, Mari Ivett, Maria Balac, Maria Barbot, Maria Cameron, Maria Carolina Lacerda de Castro, Maria Dobrzycka, Maria Eduarda Ferreira Bruco, Maria Gomes Rodrigues, Maria Gorniok, Maria haider, Maria Hickland, Maria Kent, maria medeiros, Maria Pador, Maria Silva Fernandes, Maria Skaria, Maria Sophia Gonsalves, Maria Stokoe, Mariam Adamu, Mariam Alireza, Mariam B Khan, Mariam Islam, Mariam Khan, Mariam Omar, Mariam Sabry, Mariam Sewaha, Mariana Pilakoutas, Marie Tolan, Marie-Claire Bradley, Mariella Fortune-Ely, Mariella Williams, Mariet Emmanuel, Marie-Therese Angus, Marina Billings, Marina Ghattas, Marina Lewis, Marina Nagiub, Marina pagaki, Marios Erotocritou, Marissa Frances Mansfield, Marius Pezard-Snell, Mariya George, Mariyam Azha Ibrahim, Mariyam Hussain, Mariyam Mujeeb, Marjorie Su Yin Teo, Mark Baker, Mark Coulson, Mark Futcher, Mark Hanna, Mark Hearn, Mark Hutchinson, Mark Irwin, Mark McCulloch, Martha Page, Martin F M Tan, Martin Kopernicky, Martin Rao, Martin Rodgers, Marwa Khan, Marwenie Petalcorin, Mary Candice Tirona, Mary Keast, Mary love Uzoma Ezekwesili, Mary Nnenna Aniaku, Mary Ovakimian, Maryam, Maryam Abdallah, Maryam Ghani, Maryam Jameal, Maryam Kashif, Maryam Patel, Maryam Rizvi, Marzieh Ezzati, Masako Shimato, Mashaad Coomaraswamy, Masooma Ali, Massimiliano Sante Fornasiero, Masuma Fazel, Matan Bone, Matei Tudor Berceanu, Mathew Kunnel Jomon, Mathew Walne, Matilda Brock, Matilda Chen Cooper, Matilda Hallett, Matt Corsellis, Matt Whitelock, Mattea Heck, Matthew Allen, Matthew Begbie, Matthew Brazkiewicz, Matthew Bye, Matthew Chakraverty, Matthew Corkill, Matthew Corns, Matthew Forrest, Matthew Hackney, Matthew Hennessy, Matthew J Kane, Matthew Kalirai, Matthew McKay, Matthew Pagan, Matthew pottage, Matthew R D Lee, Matthew Rendell, Matthew Rodgers, Matthew Russell, Matthew Singh Benning, Matthew Tucker, Matthew Wane, Matthew Williams, Matti Kalo, Maude Calveley, Maureen Cheruiyot, Maurie Kuha Kumaran, Mauzammal Tahiri, Mavish Khalid, Max Bennett, Max Hayden, Max Lenoir, Max Levy, Max Tagg, Maxim Julea, Maxim Minakov, Maximilian Ellacott, Maximo Clark, Maxine Meju, Maxx Chin, May M. Thu, May Schymczyk, Maya Baroudi, Maya Dudko, Maya H Shingadia, Maya Kaur Dhaliwal , Maya Robberstad, Mayank Mishra, Mayra Gisselle Ayala Brandao, Mayu Uemura, Maziyar Saeedifard, Mc Stephen Padilla, Meenusha Shanmuganathan, Meera kansagra, Meera Selvarajah, Meera Sodha, Meg Ormond, Meg Parkin, Megan Battenfield, Megan Benson, Megan Burn, Megan Burns, Megan Cornwell, Megan Cuerden, Megan Cuffe, Megan Guest, Megan Heague, Megan Hemingway, Megan Hollands, Megan Jegaradsan, Megan Kilburn, Megan Littmoden, Megan Mudd, Megan Parry, Megan Redcliffe McLean, Megan Rhiannon Shaw, Megan Rose Perkins, Megan Scotcher, Megan Simpkins, Megan Spearman, Megan Szekely, Megan Tinsley, Megan Waite, Megan Wallace, Megha Prakash, Meghana Ganeshan, Meghana Sivakumar, Meghna, Meghna Patel, Meghna Ray, Meghna Thakrar, Meghnaa Hebbar, Mehak Dhiman, Mehak Kakwani, Mehdi Khan, Mehjabin Rahman, Mehreen Parveen, Mehrqand Shahid, Mehul Panicker, Mehvish Jamal, Mei Chen Teresa Jiang, Mei Shin Yau, Mei-yin Gruber, Mel H S, Melanie Tran, Melika Nasr, Melina Bendz, Melissa Drake, Melody Su, Memory Moyo, Meng-Chieh Wu, Meryemnur Enver, Meshva Amin, Methushaa Suthanthirakumaran, Mi Joo Choi, Mia Davies, Mia Harada-Laszlo, Mia McDade-Kumar, Mia McLaughlin, Mia Paget, Mia Quinn, Mia Rose Stead, Mia Thomas, Micha Vidot, Michael Adebayo, Michael Ashfield, Michael Bailey, Michael Beisty, Michael Blayney, Michael Boal, Michael Chin Hwee Quah, Michael Cooke, Michael Dowling, Michael George, Michael Jiang, Michael Kerr, Michael Masekenuba, Michael Moody, Michael Ogunjimi, Michael Omosule, Michael Roberts, Michael Taylor, Michael Taylor, Michael Tomaschek, Michael Whitmore, Michael Woodmass, Michaela May Aurelio, Michaela Monaghan, Michaela Rogers, Michal Kawka, Michalis Chatzimatthaiou, Michele Chan, Michelle Abolarin, Michelle mariam varghese, Michelle Riska, Miguel Angel Rodriguez Ruiz, Milad Mossanen Parsi, Milan Jacob, Milap Kamlesh Rajpara, Milindu Wickramarachchi, Millicent Banks, Millicent Morris, Millicent Rayner, Millie Duckett, Millie Holding, Millie McDonald-Webb, Min Ju Lee, Min Su Kim, Mina Abdul Hussein, Mina Ragheb, Mingyue Ma, Minouche maki, Minura Abeykoon, Mira Patel, Mirabel Ruo-Yi Dale-Jones, Miranda Garratt, Miray Kirollos, Miriam Gill, Mirna Elghobashy, Misbah Manzoor, Misbah Shafique, Misha Iyer, Mitali Bajaj, Mitchell Rudo Tokonyai, Mithra Punniamoorthy, Mithraan Sunthara Moorthy, Mitra Rajnarinesingh, Mitveer Gill, Moa Lagenfelt, Modupe Obideyi, Moe Takenoshita, Moe Thidar Aung, Mohamad Abou-Eid, Mohamad Amin bin Mohamed Abu Baker, Mohamad Modar Kattan, Mohamed Abdi, Mohamed Baalawi, Mohamed Essa S/O Mohamed Zarook, Mohamed

Mansoor, Mohamed Rashad Ramali, Mohamed Shahin, Mohamed Suleman, Mohammad Ali Ahsan, Mohammad Alradhawi, Mohammad Emir Imran, Mohammad Hossein Asadi Fard, Mohammad Jameel Rahmatullah Suhotoo, Mohammad Momin Tassadaq, Mohammad Moobeen Salim Mudhawo, Mohammad Muneeb Abrar, Mohammad Nofal Usman, Mohammad Ramzan, Mohammad Saud Khan, Mohammad Sikder, Mohammad Yaqub, Mohammed Abiduz Zaman, Mohammed Al-Amin Abdal, Mohammed Araiz Imran, Mohammed Ehsaanuz Zaman, Mohammed Hamidul Islam, Mohammed Hassan, Mohammed Lester, Mohammed Minhajur Rahman, Mohammed Mustakim Islam, Mohammed Quhill, Mohammed Ridwan Rahman, Mohammed Salim Suleman, Mohammed Salmaan Azam, Mohammed Samiul Islam, Mohammed Saqalain, Mohammed Shoaib Sheraz, Mohammed Umar Pervez, Mohammed.A.H.Khan, Mohammod Nadim Chowdhury, Mohd Firdaus, Mohd Syafie Bin Mohd Nazley, Moheb Gayed, Mohini Bhagalia, Mohit Achanta, Mohit Sembi, Mohshin Syed, Moira Pitt, Moksh Sharma, Moliu Ren, Molli Kaur, Mollie Delaney, Mollie Spittal, Molly Allan, Molly Bradbury, Molly Clough, Molly Cumming, Molly Graham, Molly Jade Castagna, Molly Lim, Molly Reckord, Molly Reeve, Molly Thornton, Molly van der Heiden, Momin Ahmad, Momina Javid, Moniba Asgari, Monica Mekhael, Monika Johal, Monique Dingelstad, Monique Lucas, Monisah Ahmed, Monty Barker-Pilsworth, Monty Matson, Moonga Hamukoma, Morgan Evans, Morgan Harrison-Holland, Morven Findlay, Morven Stewart, Movin Peramuna Gamage, Mr Nathan Allinson, Mridul Rana, Mrinalini Panthula, Mu'Azzamah Ahmad, Muhammad Abdullah, Muhammad Ali Shah, Muhammad Arham Sahu, Muhammad Bojang, Muhammad Farhan bin Iman, Muhammad Karim, Muhammad Rafeeq Kureemun, Muhammad Shariq Rahemtoola, Muhammad Yousaf Khalid, Muhammad Zarnab Munawar, Muhammed Riyad Hasan, Muhsina Ahmed, Muin Ahmed, Mujahid Khan, Mukarma Zakria, Muna Abraham, Muna Yusuf, Munem Azad, Munib Elneser Chehade, Munir Khan, Munira Mohamud, Munsif Mansoor, Muntaha El-Haroun, Muqeet Enver, Murad Othman Elmaryami, Murray MacKay, Musab Abrar, Muskan Sapra, Muskan Sharma, Mussa Mohamed, Mustafa Khadr, Mustafa Majeed, Mustafah Kazi, mustapha el yaman, Mwaka Babasanya, Mya Bhamra-Gahle, Myles Dixon, Myles Neary, Myles Rice, Nabeela Bhaloo, Nabeela Farooq, Nada Jodeh, Nada Khalil, Nadeem Gire, Nadia Ali-Naja, Nadia Chaudhury, Nadia Crolla, Nadia Maria Fatima Benhebil, Nadia Nafakh, Nadia Saloojee, Nadia Shaik, Nadia Taha, Nadia Vryoni-Dickson, Nadia Zaman, Nadine edmunds, Nadine Formosa, Nadine Soliman, Nadir, Naeem Shah, Nafees Khan, Nafisa Barma, Nafisa Hussain, Nahid patel, Nahima Miah, Nahiyan Syed Pasha, Nahl Lusangi, Naiha Sharry-Khan, Naima Hamid, Naima Omer, Naina Narayanan, Naireen Asim, Najeeba Tahzeeb Salma Lallmahomed, Najma Ahmed, Namithaa Sunil Kumar, Namratha Kaur, Nancy Liu, Nandaja Narayanan, Nandhitha Ponsailapathi, Nandini Roy, Nantheesh Puvaneswara, Naomi, Naomi Atkins, Naomi Buddle, Naomi Bull, Naomi Knox, Naomi Morka, Naomi Tabitha Mohanarajah, Naomi Tooke, Naomi Wing-Kwan Tse, Naqea Nabilah Binte Muhammad Faishal Khan Surattee, Nargis Khatoon, Narmathan Rajeswaran, Nashra Maheen, Nasser Rachedi, Natalia Chilal, Natalia Makhdoom, Natalie Ko, Natalie Amanda, Natalie Carolan, Natalie Gill, Natalie Grundmann, Natalie Menic, Natalie Miles, Natalie Peckett, Natalka Krusche, Natania Yeshitila, Natasha Aghtarafi, Natasha Ashbridge, Natasha Chandrakant Patel, Natasha Clarke, Natasha Dixon, Natasha Elks, Natasha Hall, Natasha Limbachiya, Natasha McGowan, Natasha Mundell, Natasha Nwosu, Natasha Singh, Natasha Smyth, Natasha Tai Chaudhury, Natasha White, Natawadee Chantima, Nataysia Mikula-Noble, Nathalie Badger, Nathalie Burt, Nathan Abbey, Nathan Anorson, Nathan Carey, Nathan McNamara, Nathan Robinson, Nathan White, Nathan Willis, Nathan Yung, Natnaree Sihsobhon, Navdeep Rana, Naveed Amla, Naveen Bakshi, Naveenaa Sivanesan, Navindi Fernandopulle, Nawaz Safdar, Nazifa Anjum Chowdhury, Nazmin Rahman, Nazrin Uddin, Neal Patel, Nedah Simeen, Neera Chaudhary, Nefeli Eirini Perdikari, Negin Gholampoor, Neima Ebrahimian-Roodbari, Nekmee Gunasekera, Nelema Uddin, Neve Lawrence, Neysa Patel, Ngatangue Kahuku, Ngawang Dheden, Nia Evans, Niall Cameron, Niall Heyes, Niall O'Hagan, Niall P Burke, Niamh AS Heneghan, Niamh Downing, Niamh Mackdermott, Niamh McAllister, Niamh McConachie-Smith, Niamh McCool, Niamh McCormack, Niamh Pang, Niamh Roberts, Niamh Roberts, Niamh Sargeant, Niamh Sorcha Gibson, Niccolo Doe, Nicholas Butterworth, Nicholas Dalmon, Nicholas Edmond, Nicholas Farr, Nicholas Graves, Nicholas Honey, Nicholas Hubble, Nicholas Lowe, Nicholas Mackenzie, Nicholas Marshall, Nicholas Ng, Nicholas Ting, Nicholas Yeo, Nick Sanderson, Nick Shu Ki Lung, Nick Yapp, Nicola Aghaji, Nicola Bowman, Nicola Hall, Nicole Argent, Nicole Bryce, Nicole Cripps, Nicole Ellen James, Nicole James, Nicole Lee, Nicole Robin, Nicole Shelley Apostol Handy, Nicole Wang, Nicoleta Lotca, Nicolle Marianne Arroyave Caicedo, Nidhi Agarwal, Nidhi Desai, Nidhish Jeyin, Niha Mariam Hussain, Nihal Chanian, Nihal Mehra, Nihar Vobilisetty, Niharika J.K.Tekchandani, Niharika Jitender Kumar Tekchandani, Niharika Singh, Nikan Golesorkhi, Nikhil Agarwal, Nikhil Anand, Nikhil Anand, Nikhil Bashir, Nikhil Singh, Nikhita Moudgil, Nikita Choudhary, Nikita Cliff-Patel, Nikita Jha, Nikita Padmakumar, Nikita Subhani, Nikita Verma, Nikitha Benny, Nikki Jane May Stanley, Nikolas Muller, Nikolas

Theologis, Nila Jambulingam, Nila Ramanathan, Niloufar Farahani, Nimalesh Yogarajah, Nimisha Shaji, Nimra Ahsan, Nimra Akram, Nina Pollok, Niraja Lal, Nirvana Proag, Nisar Ahmad Sherzad, Nishaanth Dalavaye, Nishanthika Prabakaran, Nisharthika Balasubramanian, Nishat Qurashi, Nishil Patel, Nishma Bechar, Nissa Mushtaq, Nithesh Ranasinha, Nithin Balasubramanian, Nitya Anil Nair, Nivethan Ganeshalingam, Nivian Sukirthan, Nma-Elietta Abdul, Nneoma Enekwa, Nneoma Uzo, Noah Sagua, Nomi Fischer, Noor Alwan, Noor Bakhsh, Noor Haddad, Noor Uddin, Noor-e-Maham Shakeel, Nora Alali, Nora Khattab, Norah Alhamed, Noura Elgharably, Nowshin Sultana, Nuala Morgan, Nur Atiqah binti Abdul Talib, Nur Diyanah Naqiyah Merican, Nur Farah, Nûr-al-ayn Nisar, Nurul Iman Athirah binti Ahmad Fauzi, Nyein Su Tha, O H Yousef, Obada Kattan, Oceana Fernando, Octavia Colehan, Odhrán Mac Roibeaird, Oke Obiuwevbi, Okeoghene Christophe Mafeni, Ola Takruri, OLADIPO PHILLIPS, Olaf Tomala, Olajumoke Christiana Kuye, Oleia Green, Olga Karkanevatos, Oliver Adrian Lewis, Oliver Howl, Oliver Jones, Oliver Meyer-Bothling, Oliver Reynolds, Oliver Rideout, Oliver Rookes, Oliver Sargent, Oliver Seston, Oliver Winter, Olivia A. Pestrin, Olivia Allen, Olivia Ambler, Olivia Bradley, Olivia Brady, Olivia Braun, Olivia Brooke, Olivia Cahill, Olivia Crannage, Olivia Cuddy, Olivia Eguiguren Wray, Olivia Farley, Olivia frost, Olivia Green, Olivia Hobrough, Olivia Howe, Olivia Jennings, Olivia Kent, Olivia Lauryn Taylor, Olivia Martin, Olivia McConaghie, Olivia Mooney, Olivia Nagle, Olivia R Lark, Olivia Ramackers, Olivia Ray, Olivia Ross, Olivia Stevens, Olivia Tierney, Olivia Webster, Olivia Wilkinson, Ollie Agutu, Ollie Mann, Oluwademilade Bola-Ojo, Oluwafunto Ogunleye, Oluwajenrola Arawole, Oluwapelumi Osibona, Oluwasemilore Adebayo, Oluwaseun Ikotun, Oluwatobi Adegboye, Oluwatobiloba Odeyemi, Oluwatomisin Ademiju, Oluwatomisin Adewole, Oluwayemisi Oshodi, Omar Al-Rawi, Omar Elhelw, Omar Haque, Omar Khalil, Omar Marei, Omar Mubark, Omar Salim, Omar Zacharia Carney, Omar Zakieh, Omnia Osman, Omoremi Williams, Omowumi Daniella Ashaju, On Yu Lee, Ong, Ong Ling Ann, Onyenma Ekelemnna Obiejesie, Ophelia Styles, Oran McGilly, Ore Odubiyi, Oreoluwa Mohammed, Orie Amadi, Orieanna Reeve-Chen, Orla Canavan, Orla Jenkins, Orla Mantle, Orla Tiernan, Orlaith Breen, Orlaith Murray, Osama Suwar, Osamuyimen Ruth Omoragbon, Oscar Bugg, Oscar Daglish, Oscar McKeagney, Oscar Oglina, Oscar Waugh, Otisz Adam, Ovin Baratha Jayawardena, Ovini Charithma Weerasinghe, Owen Conlan, Owen Kennedy, Owen Richards, Owen Stait, Owen Thomas Leary, Oyinade Adeshina, Oyinkansola Oremakinde, P Das, Paapa Appiah-Odame, Paige Wilkins, Pak Tsz Yu Eunice, Pak Wing Leung, Palida Jantarang, Pallavi Bhatia, Pallavi Singh, Pamela Sturges, Pamith Ahangama Arachchilage, Paras Agarwal, Parisa Zamani, Parisha Blaggan, Parmis Lamei Javan, Parvathi Thara, Patricia Lapitan, Patrick Copley, Patrick Devlin, Patrick Galway, Patrick Greene, Patrick Kerr, Patrick Kielt, Patrick Lynch, Patrick Monaghan, Paul Crawford, Paul Holmes, Paul Ingley, Paul McKendrick, Paul Njoku, Paul O'Connor, Paul Sanjay Sundaram, Paul Ward, Paul Wong, Paul Zaki, Paula Adodo, Paula Grill, Paulina Cecuła, Pauline Galelemogwe, Pavan Kaur Marwaha, Pavandeep Kaur Virdee, Pavithra Sakthivel, Payal Guha, Payam Soleimani-Nouri, Pearl Jilla, Penelope P J Sucharitkul, Perina Tilija, Peru edmondson, Peter Barfoot, Peter Clements, Peter Crabtree, Peter Johnston, Peter McClung, Peter McManus, Peter Sloan, Petra Popovic, Petros Georgiou, Philip Boesch, Philip Harrison, Philip Quither, Philippa Banks, Philippa Clayton, Philippa Hatton, Philippa Jackson, Philippa Morris, Phoebe Avbulimen, Phoebe Cope, Phoebe H, Phoebe Kottler, Phoebe Langdale, Phoebe Maxwell, Phoebe Paley, Phoebe Stocker, Pinky Kotecha, Piraya Saereesithipitak, Poh Wei Quang, Pon Suek Keen, Pooja Patel, Pooja Sunildath, Pooja Swarup, Poojaa, Poppy Bowler, Poppy Brown, Poppy Gotto, Poppy Kemp, Poppy sheppard, Prabhav Singhal, Prachi Agarwal, Pragalva Khanal, Pragna Kasetti, Prakhar Kumar, Prakrit Kumar, Prakruthi Prasad, Pranav Ponvannan, Pranav Satish, Pranav Sudheesh, Prapti Gurung, Prarthana Venkatesh, Prasadini Fernando, Prashanthan Balendran, Prashasth Cheekoty, Pratheeshan Sabeshan, Pratyush Pradeep, Praveena Mahalingam, Precious Jolugbo, Precious Quaye, Preena Patel, Preethi Ann Jacob, Preminie Ellengho, Pretesh Patel, Prina Singh, Prince Jiju, Prince Josiah Joseph, Princess Imoru, Priscilla Djan, Prithvi Dixit, Prithvi Venkateswaran, Prithvirao Sonoo, Priya Chakraborty, Priya Patel, Priya Rose Babu, Priyadarshini Shomi Basu, Priyanga Biju, Priyanka Bikkannavar, Priyanka Iyer, Priyanka Kaushal, Priyanka Kissoonsingh, Priyanka Trivedi, Priyankkha Muniyappan, Priyanth Alaguraja, Priyasha Sen, Priyesh Agravat, Prudence Leung, Pubudu Piyatissa, Puneh Shahrjerdi, Puntrika Tannirandorn, Purva Balu, Puvanitha Puventhiranathan, Pyaesone Nyunt, Pylin Parkes, Qaiys Abu Qaoud, Qasim Ahmed, Qasim Hussain, Qi Zhuang Siah, Qian Ying Tang, Qing Tan, qudsia bhatti, Quentin Mak, Quinston Lee, Raashi Shah, Rabbiya Shahzadi-Sohail, Rabia Ali, Rabia Rubbab, Rachael Annandale, Rachael Henderson, Rachael Hong Pei Wen, Rachael J Trowton, Rachael Williams, Rachel Addington, Rachel Allison, Rachel Boscott, Rachel Burnley, Rachel Byrne, Rachel Casey, Rachel Crooks, Rachel Crothers, Rachel Elwood, Rachel Evnas, Rachel Graeme-Wilson, Rachel Grayson, Rachel Hubert, Rachel Jones, Rachel Kelly, Rachel Lee, Rachel Narunsky, Rachel Ng Xin Hui, Rachel Qian Feng Low, Rachel Rotrand, Rachel Shepherd, Rachel Sinha, Rachel Smyth, Rachel Strahan,

Rachel Su Min Lee, Rachel thavayogan, Rachna D Maywah, Radeyah Anjum, Radhika Tandon, Radhika Tanna, Raeesah Maqsood, Raeesah Meghjee, Raffaella Melilli, Rafi Abdullah, Rafia Ayub, Raghad Al Taie, Ragini Soni, Raha Tarighi, Raheel Faiz, Rahul Herlekar, Rahul Panikker, Rahul Patel, Raian Jaibaji, Raihan Mohammed, Raihan Pulakal, Raiyah Ismaiel, Raja Ali Chan, Rajan Pal, Rajan Phakey, Rajan Swami, Rajashree Murki, Rajinder Gill, Rajveer Singh Sehmbi, Rajwant Kaur, Rakan Al-Asa'ad, Raksha Naik, Rakshita Agrawal, Rakul Sri, Ram Aswani, Rama Jha, Rama Lakshman, Ramanish Ravishankar, Rameez Naqvi, Ramla Abdourahman Mairama, Ramneek Sahota, Ramnik Uppal, Ramsha Ahmed, Ramya Narayanan, Rana Al-Khayat, Rand Abadi, Rand Abadi, Rand Al Assadi, Randeni Arachchige Adithi Himika Randeni, RANJEET BHAKAR, Raphael Cheng, Rapik IV Mangaser, Rashna Tashfeen, Rashne Ravindran, Raunak Jain, Raunak Mukherjee, Ravanth Baskaran, Raveena K Khalsa, Ravi Mandalia, Ravi Shah, Ravinder Kaur, Rawan Al Dehailan, Rawan Ebrahim, Rayan Kamal, Rayma Risquet, Raymond Zhylin, Rayna Kaur Bhogal, Razna Ahmed, Rebecca Amy Clifford, Rebecca Black, Rebecca Bramley, Rebecca Bushell, Rebecca Byrne, Rebecca Chloe Bryant, Rebecca Clough, Rebecca Farrugia, Rebecca Hakim, Rebecca hawksworth, Rebecca Hobson, Rebecca Hotchkin, Rebecca Houghton, Rebecca J Nock, Rebecca Jackson, Rebecca Jess, Rebecca Kelly, Rebecca Kemp Arnold, Rebecca Ketteman, Rebecca Leach, Rebecca Ludden, Rebecca M Curtis, Rebecca McCullagh, Rebecca Minshull, Rebecca Naisj, Rebecca Nicholls, Rebecca O'Dell, Rebecca Osborn, Rebecca Osmond, Rebecca Pearce, Rebecca Richardson, Rebecca Stanger, Rebecca Tait, Rebecca Vitarana, Rebecca Wood, Rebecca Wright, Rebekah Ann Mathews, Rebekah Clenaghan, Rebekah Dimmock, Rebekah Kenny, Redwan Hayat, Reea Khanna, Reece Patel, Reece Smith, Reem Aboushousha, Reem Alhababi, Reem Jaafar, Reem Moussa, Reem Shamel, Reena Patel, Reeshma Jameel, Reeves Spencer Campbell, Reeza Khan, Rehana Byrne, Rekha Gurung, Remarez Sheehan, Remi Simpson, Renée Alejandra Servin Recio, Renée Defreitas, Renee Okhiria, Renee Siu Yu Ma, Renier Visser, Renske Mcfarlane, Reshma Azim, Reuben Amoaku, Rhea, Rhea Hunjan, Rhea Narang, Rhea Sarah Jacob, Rhea Shukle, Rhea Srinivasan, Rhian Davies, Rhiane Berchie, Rhianna Madden-Hansle, Rhiannon Kirk, Rhiannon Murphy, Rhiannon Tanner, Rhiannon White, Rhys Elgumati, Ria Patel, Ria Prajapati, Ria Shemar, Ria Shivam, Ria Valecha, Rianne Jasmine Patel, Riaz Nawaz, Richard Ford, Richard Huynh, Richard John Francis Metters, Richard Mifsud, Richard Molyneux, Richard Mussard, Richard Phillips, Ricky Singh, Rida Bukhari, Rida Bukhari, Riddhi Deshpande, Rifat Ershad, Rihazur Rahman, Rikesh Jagatia, Rilind Berisha, Rimsha Ali, Rimsha Aziz, Rio Sherman, Riona Linn, Ríonach McCarron, Risa Suwannaraks, Rishab Balaji, Rishabh Gupta, Rishana Thayaparan, Rishi Kaushik, Rishi Kumar, Rishi Sharma, Rishil Patel, Risma Remsudeen, Ritika Dilip Sundaram, Ritika Gera, Ritika Roy, Ritu Khanna, Rivya Mathews, Riya george, Riya Ghose, Riya Patel, Riya Thomas, Rob McFarlane, Robbie Uriarte, Robby Goh, Robert Barton, Robert Chan, Robert David Forbes Smith, Robert Ingram, Robert James Grogan, Robert Kay, Robert Smith, Robert Zhan, Robin Bendix-Hickman, Robin Caley, Robin Hext, Robyn Chilton, Robyn Jean Senior, Robyn Powell, Rogan Dean, Rohaan Shahzad, Rohan Bassi, Rohan Bhate, Rohan Mehra, Rohan Patel, Rohini Ghosh, Rohit Pawar, Roisin McKenna Favier, Róisín Peberdy, Rojan Ghaderi, Rokshan Easwarathasan, Roland Amoah, Roma Thakker, Romana Jan, Romilly Hayward, Romilly Sherrington, Ronak Shah, Ronald H.K. Nam, Ronan Eves, Ronan Harrison, Ronan Yeo, Rooksarr Beelontally, Roosindu Peris, Rory Cairns, Rory Houlihan, Rory McNicholas, Rory Purves, Rosalind Tang, Rosanna Watts, Rose Ameli, Rose Bird, Rose How, Rose Kurian Thomas, Rose Maloney, Rose Oconnell, Rose Stacey, Roseanne Ismail, Roselyne Ananda, Rosemary Bell, Rosemary Dale, Rosemary Fung, Rosemary Ho, Roshni Goodka, Roshni Johnson, Roshni Patel, Roshni Patel, Roshni Patel, Roshni Rahman, Rosie Blount, Rosie Gouldstone, Rosie Hall, Rosie Hobster, Rosie Meakins, Rosie Middleton, Rosie Mogridge, Rosie O Johnson, Rosie Osborn, Rosie Parnham, Rosie Rainer, Rosie Solomon, Rosie Wilson, Ross Carrier, Ross Gillespie, Ross Moore, Ross Roxby Simpson, Ross Wilson, rouda abdalla, Rowan Daniels, Rowan Hall, Ruairi Kelly, Ruairidh Mackay, Rubab Zafar, Ruby Hill, Ruby M Lawrence, Rufus Robinson, Ruhullah nasiri, Rukevwe Udu, RUMAISA ZUBAIRI, Rumaysa Patel, Rumman Ahmed, Rungphloy Jaroenchasri, Runil Shah, Rupert Jordan, Rupert Vicary-Watts, Ruqayyah Jabeen, Ruqayyah N Beg, Rushaa Mooniaruth, Russell Chalmers, Russell Gee, Ruth Allam, Ruth Cheryl Pravinkumar, Ruth Goh, Ruth Henderson, Ruth Muan, Ruth Oyenuga, Ruth Strain, Ruth Vanessa De Silva, Ruth Warren, Ruvini Moragoda, Ryan Beggs, Ryan Bellman, Ryan Bhalla, Ryan Chien, Ryan Duffy, Ryan H McMillan, Ryan Habermann, Ryan Hong Yue Lim, Ryan Mamun, Ryan Nolan, Ryan Turner, Ryan Wolff, S Gholamian, S Hasselder, S Yasmin, S.F. Shaida, S.Sujitha Pillay, Saad Abbas, Saad Ahmad, Saad Dosani, Saade Mohammed, Saahil Kaura Bali, Saarah Talha, Saba Azizi Torkanpour, Saba Khan, Saba naji, Saba Tasleem, Sabah Rafique, Sabah Sirajuddin, Sabea Morgan, Sabina Christou, Sabina Luszczak, Sabine Bellamy, Sabrina Ahmed, Sabrina Forrester, Sabrina Khawar, Sabrina P Sookramanien, Sacha Salamon White, Sacheth Menon, Sachin Amonker, Sachin Chacko, Sachin Chambers, Sachin Jalota, Sachin Sabu, Sachini Pattiya Gardi Hewage, Sadhana Bur, Sadia Afrin

Joulhash, Sadia Ahmed, Sadia Qalandari, Sadia Zaman, Sadie Leonard, Sadiqa Alam, Saffron Hicks, Safwan Shaikh, Sagana Easwaran, Sagar Sanadi, SAHID KAMARA, Sahil Chandra, Sahiti Kalapu, Sai Kollipara, Sai Manikanta Siddharth, Saif ali, Saif Ansari, Saife Salem, Saiful Khan, Saiiswari Sivanesan, Sailantra Raj Sivathasan, saira ahmed, Saira Hussain, Sairana abraham, Sajed Mangoo, Sajeenth Vishnu K, Sajid Ibrahim Member, Sajid Member, Sakar Salar, Salah Khogali Hassan, Saleh Alsmaieel, Salem Elias, Salihah Ahmad, Sally Chapple, Sally Joyce, Salma Abdirizak, Salma Amer, Salma Khatun, Salma Miah, Salma Yusuf, Salman Khalid, Salomé Bapt, Saloni Bhattacharyya, Saloni Nakhare, Saloni Singh, Salwa Rahman, Sam, Sam Bell, Sam Dyer, Sam Ihab Abu-Reish, Sam Slattery, Sama Bhatia, Samaa Musa, Saman Naqvi, Samanta Zahir, Samantha Haines, Samantha Irvine, Samantha Ross, Samar, Samar Al-Shamaa, Samar babiker, Samatar Osman, Samay Prakash, Sameerah Jawaid Khan, Samera Bi, Sami Raza, Samiha Bachoo, Samina Alim, Samina Khan, samir saincher, Samira Mendheria, Sammy Chan, Sammy Isabella Gharbieh, Samodani Wijetunge, Samra Abbass, Samrat Prasai, Samreen Fathema, Samual Reynolds, Samuel Addelman, Samuel Afari, Samuel Bloomer, Samuel Bradley, Samuel Charles Guymer, Samuel Goundry, Samuel James House, Samuel Kenmore, Samuel Marc Lucking, Samuel Moore, Samuel Ololade, Samuel Parker, Samuel Powell, Samuel Soete, Samuel Tushingham, Samuel Wood, Samy Cheikh Youssef, Samyak Verma, San Kim, Sana Hussain, Sana Khan, Sana Mariam Baboo, Sana Syed, Sanaa Ghori, Sandra Mathews, Sandra Shibu, Saneaah Khan, SangHyeok Lee, Sanika Nair, Sanita Sandhu, Saniya Saiyed, Sanjana Chinari, Sanjana Ilangovan, Sanjana Kamath, Sanjana Murali, Sanjana Voonna, Sanjay Sahdev, Sanjiv Sharma, Sanna Masood, Sannah Ameen, Sannah Jamil, Sannah Mahmood, Sanskrithi Sravanam, Santhosh Ayirookuzhi, Sanuja Sapkota, Saqlain, SARA AHMAD, Sara Alexandra Wain, Sara Celyn Jones, Sara Eleri Morgan, Sara Fernandes Diez, Sara Gadalla, Sara Hafiz, Sara Hussain, Sara ibzea, Sara Memon, Sara Obudi, Sara Paiva Sousa, Sara Patel, Sara Sarhadi, Sara Wasim, Sara Yahia Mohammed, Sara Zafar, Sarah Aldelemi, Sarah Allen, Sarah Brooks, Sarah Bufton, Sarah Bunnewell, Sarah Driscoll, Sarah Emerson, Sarah Ferbrache Namono, Sarah Gaier, Sarah Hamza, Sarah Hignett, Sarah Hoban, Sarah Hobbs, Sarah Hussain, Sarah Ibrahim, Sarah Ish-Horowicz, Sarah Ives, Sarah Karrar, Sarah Kelly, Sarah Khan, Sarah Knight, Sarah Kwia, Sarah Leathem, Sarah Lloyd, Sarah Madden, Sarah McCarthy, Sarah McClean, Sarah Mehmood, Sarah Michael, Sarah Mohamed-Mustak, Sarah Moin, Sarah Musbahi, Sarah Neal, Sarah O'Hagan, Sarah Pedley, Sarah Pengelly, Sarah Robinson, Sarah Rutherford, Sarah Smyth, Sarah Smyth, Sarah Stephen, Sarah Tai-MacArthur, Sarah Utulu, Sarah Venning, Sarah Walker Date, Sarah Zein, Saranya, Saranya B Rajendran, Saranya Baleswaran, Saranya Sivapalan, Sarina yao, Sasha Bill, Sasha Chard, Sasha Loeffen-Ames, Sasha Marks, Sasha Quarrington, Sashya Kandanearachchi, Saskia Oakley, Saskia Swannack, Satia Babu, Satishvaran Vijhayan, Satnam Rai, Saumiya Kesavan, Savan Shah, Savannah Roeber, Sayeda Nahar, Scarlett Marshall, Scott C Mackenzie, Scott New, Scott Ryan Booth, Sean Benjamin Lang Farrell, Sean Lobo, Sean Mahoney, Sean Williams, Sean X. Liu, Sebastian Fox, Sebastian Machin, Sebastian Priest, Sebastian Ritchie, Seena Saberi-Movahed, Sehaan Hannan, Sehar Nazir, Seifaldin Ali, Seijal Patel, Selim Arslan, Selin Tezcan, Selina Robertson, Seline Ismail-Sutton, Senula Madhavan, Serena Baker, Serena Mann, Serena Patel, Serene Batson-Patel, Seri Park, Serisha Sorby, Serra Theoharopoulos, Setthasorn Zhi Yang Ooi, Seunghee Han, Severine Matthews, Seyi Adeleye, Shaan Chhabra, Shaara Ahmed, Shabnam Tariq, Shadia Patel, Shafiah Ali, Shafiea khan, Shahid Ali, Shahnia Surendran, Shahriar Mueed, Shahzaib Ahmed, Shahzeb Ali, Shaini Subendran, Shaishab Sharma, Shakira Fleming, Shalimar Baldemor, Sham Rahman, Shamas Hussain, Shambavi Premjeyanth, Shameer Mohamed Naleer, Shamhethan Bhaskaran, Shamita Suresh, Shammah Fatimah Khadaroo, Shamus Butt, Shanen Emmanuel, Shani Brooks, Shani De Soysa, Shanie Khalid, Shankari Gnanakumar, Shanmathi Mahesh, Shannen Smyth, Shannon Butler, Shannon Devlin, Shannon Vose, Sharan Syam, Sharanniyan Ragavan, Sharika Sundar, Sharuha Kumarasamysarma, Sharuka Ravichandran, Shashini costa, Shaun Chad Lee, Shaweena Shaari, Shayaan Akhtar, Shaza Hendy, Shefali Kotecha, Shehab Ismail, Shehan Tittagala, Sheheryaar Ahmed, Shehr-Yar Durrani, Sheina Kaye, Shekinah Osuchukwu, Shen Chuen Khaw, Shenelle Wickramarathna, Shenoo Begum, Sherie Prema George, Sherilyn Joy Hsien Lin Chew, Sherin Thambu, Sheryl Higham, Sherzah Jamal, Shi Pei Loo, Shifona Kumarasingam, Shikha Nishith, Shilpa Raj, Shina Ardani, Shiny Darwin, Shiraz Shafi, Shiree Khinder, Shireen Gamadia, Shirley Yadu, Shiv Hadani, Shiva Namdeo, Shiva Pandey, Shivali Gadhia, Shivam Ghosh, Shivam Shah, Shivamuralitharan Pirapaharan, Shivang Shastri, Shivani desai, Shivani Mahapatra, Shivani Pedda Venkatagari, Shivani Rajkumar, Shivani Shukla, Shivika Sharma, Shoaib Hussain, Shona MacTavish, Shona Mitchelmore, Shoumita Bardhan, Shreesh Sinha, Shreeya Kotecha, Shrey parmar, Shreya Agrawal, Shreya Patel, Shreya Saraf, Shrina Patel, Shriya Puranik, Shrujal Jain, Shruti Attarde, Shu Hui Lee, Shubham Agwan, Shubham Gupta, shubhi ratra, Shujaa Khan, Shukry Farah abdi, Shun Hong Chiew, Shun Qi Yong, Shuyang Dai, Shuyi Zhen, Shyam Iyer, Shynica Thayalan, Siân Hopper, Sian Roberts, Sicelo Biyela, Siddartha Paul, Siddharth Tyagi, Sidiq Aboobaker,

Sihab Reza, Sikandar Khan, Silo Firat Dogan, Sim Jia Ying Sharmaine, Simi, Simi Sodeinde, Simon Barlow, Simon Nicolas Ranjithkumar, Simon Saldanha, Simon Williams, Simone Ahmed, Simran Dhaliwal, Simran Kang, Simran Kaur Athwal, Simran Kaur Longani, Simran Kaur Panesar, Simran Kaur Singh, Simran Shrestha, Simrita Agrawal, Simron Kaur, Sina Hosseinzadeh, Sina Karimian, Sindiya Siva, Sinduja Sountharamoorthy, Sinead Carton, Sinéad Foley, Sinead Mcsorley, Sinead Wright, Sinjini Basu, siobhan adeyemi, Siobhan Hazel Munro, Siôn Gilbey, Siona Mitra, Sirah Aksa Shaheen, Sita Asi, Siva S R Dwarampudi, Sivarajini Inparaj, Siyang Xu, Siyoung Rachel Lee, Skanda Rajasundaram, Smarika Tuladhar, Smita Khilar, Sneha Mallya, Sofia Breeze, Sofia Gron, Sofia Mashanovich, Sofia Tentativa, Sofia Terreros-Martin, Sohail Singh, Soham Sarkar, Sohema Moosa, Sohini Gajanan Pawar, Soliu Oyeleke, Soma Farag, Somar Albani, Somya Sharma, Sona Streather, Sonam Patel, Sonia Akther, Sony Roy, Sophanit Pepple, Sophia Boyce, Sophia Brenac, Sophia Chen, Sophia Holson, Sophia Raymond, Sophia Williams, Sophia Wong, Sophia Xia, Sophie Antonia Barclay Erdmann, Sophie Beardall, Sophie Bell, Sophie Berson, Sophie Birch, Sophie Blummers, Sophie Burgin, Sophie Coull, Sophie Davenport, Sophie Ettinger, Sophie Harris, Sophie Hebden, Sophie Heritage, Sophie James, Sophie Jenkinson, Sophie Kirby, Sophie Langdon, Sophie Lansley, Sophie Leiner, Sophie Lloyd, Sophie Maccoy, Sophie Marriott, Sophie May Gasson, Sophie McCreadie, Sophie Miles, Sophie Parker, Sophie Price, Sophie Rhys-Evans, Sophie Rivett, Sophie Rolls, Sophie Sharpe, Sophie Simmonds, Sophie Stretch, Sophie Talas, Sophie Taylor, Sophie Wakeling, Sophie Young, Soraya Albuquerque, Sorcha Heelan, Soshin Limbu, Sosipatros Bratsos, Soung Eun Choi, Sourabh Jadhav, Sowjanya Veluturla, Sowmya Prasanna Kumar Menon, Spencer Kopera, Sreedev Darsan, Sreenidhi Athi, Sreya Sam, Srikumar Murugan, Sruthi Murthy, Sruthi Ramaraju, Stacey Knight, Stanley Cole, Stavriani Bompetsi, Stephan Chee, Stephanie Bagni, Stephanie Forrest, Stephanie Griffiths, Stephanie J Larcombe, Stephanie Lee, Stephanie Murray, Stephanie Nnadi, Stéphanie Thébault, Stephanie Ying En Siew, Stephanos Ghobrial, Stephen Aguilar, Stephen Farrell, Stephen Leadbitter, Stephen McWilliams, Stephen Morrison, Stephen Oputa, Stephen smith, Stephen Tompkins, Stephie Aine, Steve Nahar, Steven Hastings, Steven Ling Fung, Steven Taylor, Steven Toh, Stevie Fong, Strathan Dino Chun, Stuart Henderson, Subhan Baig, Suchita Pandit, Sudah Ahmed, Sudiksha Devendra Kumar, Sufficient Nkomo, Sufia Laulloo, Suhaib El-Omar, Suhail Nuruddin bin Raihan, Suhir Abdulla, Sujan Sriharan, Sujata Dutta, Sujay Nataraja, Sukanya Nanchahal, Sukanya Thavanesan, Sukham Kaur, Sukhjaad Chatha, Sulaymaan Al Majid, Sulayman Ayub, Suleiman Spencer, Suliman Ahmad, Sumathi Prasad, Sumayya Manji, Sumayyah Tahsin, Sumbal Bhatti, Sumbel Khan, Sumol Sarker, Suneel Patel, Sunenah Verma, Sung Min Yun, Sunita Burgul, Sunna Ali, Sunny R. Sedani, Sunwoo Lee, Supriya Jassal, Sura H, Suraya Gafore, Surina Maharjan, Susanna Hotchkiss, Susanne Hall, Sushilkumar Keshav Jadhav, Sushma Karim, Sushobhan Adhikari, Susie Liddiard, Sut Mo Zachary Chan, Suyesh Amatya, Svetlana Lakunina, Swarna Yemparala, Swawiza Gohobur, Sweatha ananthalingam, Swetha Venkata Narayanan, Syafika Amanda Binti Ahmad Ridzuan, Syan Patel, Syed Abdullah Akhter, Syed Azhar Ali, Syed Haider Ali Bukhari, Syed Hamza Abbas, Syed Hussain, Syed Kamran Hyder, Syed Suhaib Ali, Syed Sulaiman Imam, Syed Yusuf Maududi, Syeda Anum Zahra, Syeda Maryam Zahera, Syeda Sabbah Batul, Syedah Aleena Haider, Sylvia Huang, Sylvia Misztal, Sylvia Smyth, Syon Lee, Sze Yii Liew, Sze Yin Sophia Wong, Sze Yuin Wong, Taba Khan, Tabea Winkler, Tabi Unsworth-White, Tabitha Gould, Tabitha Hill, Tabitha Walton, Tadiwanashe Mabeza, Tae-Seon Lee, Tafsir Ahmed, Tahaa Shuaib, Tahir Khan, Tahira Yasmin Malik, Tahmeed Ahmed, Tahreem Mahmud, Tahseen Idrees, Tai Hang Dominic Jasper Kwan, Taiwo Oki, Tala Hepburn, Talha Naveed, Talhah Chaudri, Tamara Shanahan, Tamika Chaora, Tamlyn Knight, Tamsin Drury, Tanaya Gandhi, Tanisha Burgher, Tanisha Patel, Tanisha Rajah, Tanith Bain, Tanmeet Chawla, Tanvi Hemant Ambulkar, Tanvi Khetan, Tanvi Sinha, Tanvir Duhra, Tanweer Kurmoo, Tanya Kalra, Tanya Obeyesekera, Tanzim Shahid, Tanzina chaudhury, Tapan Parikh, Tara Alhamami, Tara Emami, Tara Etherington, Tara McKean, Tara Pachu, Tarosha Jayasinghe, Tarun Sripadam, Tashi Choudhary, Tashi Maseland, Taslima Mona, Tasmiyah Malik, Tasnim Anwar, Tasnim Chowdhury, Tasnim Karimji, Tasnim Kouli, Tatiana Garofalidou, Tatiana Hamakarim, Tavleen wasan, Taylor Smith, Taylor Youngsmith, Tayyaba S Ali, Tazim Hoque, Tchornigh Forder, Tebogo Matsebanane, Tegen Williams, Tehseen Contractor, Tejaswi Sharma, Temi Bamkole, Temidayo Osunronbi, Temiloluwa Olawore, Temitope Fisayo, Teo Ruey Ping, Teresa Mergia, Teresa O'Brien, Tereza Eirini Takkou, Tess Blandamer, Tess Clegg, Thabia Tapadar, Thai Ha Dinh, Thalia Ballinger, Thanushan Chandraseelan, Tharun Muthu Gurunath, Tharushi Perera, Thejus George, Theo Marples, Theodora Okechukwu, Theodoros Paschalis, Theresa Awolesi, Theresa Eka, Thia Hanania, Thiara Rupasinghe, Thien Nguyen, Thieshan Godlin, Thivakar Sri Kandakumar, Thivya Thillainadarajah, Thomas Agar, Thomas Allen, Thomas B K Watkins, Thomas Baker, Thomas Brookes, Thomas Brown, Thomas Clarke, Thomas Doggett, Thomas Franchi, Thomas Georgi, Thomas Greenslade, Thomas Hall, Thomas Hall, Thomas Ho Lai Yau, Thomas Honey, Thomas Hunt, Thomas Kay, Thomas Liney, Thomas Lumsden, Thomas Mantle, Thomas Martin Murray, Thomas

McAllister, Thomas McLelland, Thomas Moreno-Stokoe, Thomas Muldoon, Thomas Proctor, Thomas Ranaboldo, Thomas Richard Williamson, Thomas Rose, Thomas Shabha, Thomas Shevlin, Thomas Westhead, Thomas Whittaker, Thonmoy Dey, Thulaseedhara Harshavardhan Macharla, Thulasi Daniel, Thushiah Krishnapillai, Tiba Qays Fadhil, Tiffany Ye, Tihami Mansoor, Tilly Hamilton, Tim Ho, Tim Neill, Tim Rutland, Tim Ruttle, Timothy Forster, Timothy Spence, Timothy Woo, Tin Chan, Tinashe Gwarada, Tinaye Gladys Mupedziswa, Tinaye Mandishona, Tinaye Mapako, Ting Yang, Titilope Jempeji, Tjasa Zaletel, Tjun Wei Leow, Tobias Corner, Tobias Smitherman-Cairns, Tobias Tan, Toby Chapman, Toby Charge-Thornton, Toby Richardson-Jones, Toby Rock, Tofunmi Sesby-Banjoh, Toluwalope Adepeju, Tom Kerfoot, Tom Langstroth, Tommy Sutton, Tomos Arfon, Tony-Harshan Linton-Jude, Tracy Lee, Travis Marshall, Trevor Pinchemain, Tricia Tay, Trisha Suji, Trishala Misra, Tung Hua Marium Yu, Tushar Hari, Tushar Rakhecha, Tyler Longbone, Tze Ming Nicholas Lau, Tzvi Reich, Uday Kataria, Uday Keshwala, Ugonna Onwuchekwa, Uma Patel, Uma Walgama, Umair Qureshi, Umar Naveed Khawaja, Umer Siddique, Umer Syed, Ummaz Nadeem, Ummulkhulsum Yahaya Ibrahim, Umnia Ahmed, Umraj Rai, Umuayman Osman, Una Doherty, Unaludo Machacha, Upjeet Mahon, Urjit Soni, Ursila Zaman, Ursula Walton, Urwaa Aleem Khan, Usaamah Ramzan, Usaamah Ramzan, Usama Ali, Usamah M Afzal, Usmaan Razzaq, Usman Nasir, Usman Shah, Utkarsha Basu, Uzair Khalid, Vaibhavee V Patel, Vaibhavi Agarwal, Vaisaly Balan, Vaishali Kiridaran, Vaishnavi hemdev, Valentina James, Valentina Silva, Valentine Uche, Valerie Aghoghovbia, Valerie Askin, Vandana Venkateswaran, Vandana Venkateswaran, Vanessa Huang, Vanessa Otti, Vani Duddella, Vanya Gurr, Vareesha Nasib, Varghese George, Varja Čučulović, Varoonan Sritharan, Vartika Garg, Vdhuja Sivabavanandan, Veda Kudva, Veena Sudarshan, Venessa Widanaralalage Don, Venson Wai Leuk Pang, Veshalee Vernugopan, Vi Nhut Truong, Vibha Shaji, Vicky tsimplis, Victor Cheung, Victor Kimani, Victoria Amy Porter, Victoria C Shaw, Victoria Diaper, Victoria England, Victoria Grace Collins, Victoria MacAskill, Victoria Maidman, Victoria Morgan, Victoria Ngai, Victoria Van Loo, Vidthya K, Vigilius Gregory Gleetus, Vignesh Surianarayanan, Vijay Kumar Gogna, Vijaya Bommireddipalli, Vina Soran, Vinay Mandagere, Vincent Ng, Vindhya Maripuri, Vineathan Perinparajah, Vinesh Sivaneswaran, Vinesha Pillai Rajendra Prasad, Vinija Thirucumaran, Vinitha Kirupaharan, Vinson Wai-Shun Chan, Vinura Kisalkumara Munasinghe, Violet Borkowska, Viraj Shah, Viral Gudiwala, Virensinh A.Rathod, Vishal Keshwala, Vishali Desai, Vishvan Naidu, Vithullan Sapaetharan, Vivek L Joshi, Vivek Mathews, Vivian Teh, Vivienne Enife Ferife, Vladimir Popa-Nimigean, Waad Ahmed Attafi, Wai Chin Ho, Wai Yu Kate Yeung, Wajid Rashid, Walid Anwar, Wan Hei Vanessa Chan, Wana Mungwala, Waqar Younas, Waqas Ali, Wares Jalil, Warren De Lima, Wasi Iftekar, Watan Chantima, Wea Gwee, Wei Yin Jessie Low, Wei Yun Teo, Weihan Ong, Wenbin Guo, Wentin Chen, Weqar Hamed Al-Wahaibi, Whitney Ihenachor, Wiktor Michalik, Will Baker, Will Bray, Will Campbell, Will Kearns, Will Madu, Will Mepham, Will Ormerod, Will Porter, William Alton, William Angwin, William Baker, William Baker, William Bell, William Caufield, William Cowmeadow, William Faux, William Fay, William Holland, William J Crawford, William John FS Shotton, William lam, William Lim, William Manifold, William Ned Harris, William Patrick Hunter, William Richards, William Riley, William van Klaveren, William White, Wilson Matsapa, Wing Kiu Chou, Wing Yu Siobhan Lau, Winny Truong, Winson Cheung, Won Young Yoon, WONG YEN YIN, Woo Yan Ting, Xane Safdar, Xavier Carr-Deed, Xin Yi Ng, Xin Yin Choo, Xiu Xian Ooi, Xutong Li, Yagmur Adalier, Yagnaseni Bhattacharya, Yajur Arora, Yaldasadat Hashemipour, Yan Jack CHUNG, Yan Lynn Ng, Yangmyung Ma, Yanika Tuli, Yanina Pecherska, Yanisa Wannasuphoprasit, Ya-ro Chen, Yasaman Niakan, Yash Bhupenkumar Kahar, Yash Sangani, Yasin Uddin, Yasmeen Al Sadek, Yasmin Awan, Yasmin Bashir, Yasmin Cantwell, Yasmin Halimah Mayouf, Yasmin Ingram, Yasmin Richardson, Yasmin Samih Sadek, Yassir Al-Mayahi, Yazan Fallaha, Yee Mon Tin Maung, Yen Jia Soh, Yeo Audrey, Yeo Zann Der, Yi Guang, Yi huen lillian lau, Yi Wah Wong, Yi Zhao, Ying Jin, YING ZHOU LIAN, Yiorgos Stathopoulos, Yip Chee Rong, Yong Yie, Liew, Yoon Soo Park, Yoon-Seo Jo, Yosra Khllat, Yosuf Sulaiman, Youngeun Lee, Yousuf Hashmi, Yousuf Khogeer, Yu Siow, Yucef Belblidia, Yue Huang, Yu-Hsuen Yang, Yuk Ting Au Yeung, Yun He, Yun Jee Koh, Yun Yan Wong, Yung Brian Lau, Yunus Ali, Yunus Hussain, Yura Shin, Yuseon Lee, Yusuf Hussain, Yusuf Kayani, Yuta Nagano, Yuxiao Alice Wang, Yvette Jaffe, Yvonne Chung, Yvonne Marsh, Yzobelle Barcelos, Z Yusuf, Zac MacLellan, Zachary Osman, Zachary Pierrepont, Zachary Santoro, Zaheer Badat, Zahira Pervaze, Zahra Cader, Zahra Faiz, Zahra Ismail, Zahra Karmally, Zahra Olateju, Zahra Ramzan, Zahra Syeed, Zahrah Anjum, Zaib Hilal, Zaid Alsafi, Zaid Haj Ali, Zain Ahmad, Zain Girach, Zain Islam, Zaina Aloul, Zaina Iqbal, Zainab Ganiyu-Dada, Zainah Fatima Moin, Zakari Francillon, Zakaria Abdullahi Gurhan, Zakeeya Munshi, Zakery Worts, Zaki Arshad, Zaki Manan, Zal Canteenwala, Zara Adil, Zara Holliday, Zara Kidwai, Zara McConnell, Zara Murdock, Zara R. Zaccariah, Zarriar Khalid, Zarshi Qaisar, Zayn Ahmad, Zayna Ahmed, Zayna Naseem, Zayna Shireen Ahmed, Zaynab Nazir Ahmed, Zaynah Naz Qamar, Zeinab Dafalla, Zeinab Mutlak, Zeynep Tugce Tugcu, Zeynep Tugcu, Zhao Xuan Tan, Zhen Xin Ong, Zheng Yang Tan, Zhin,

Copyright ©2022 BURST Research Collaborative

Zhoulu Luo, Zi Heng Tee, Zi Qi Kok, Ziad Zeidan, Zinnia Lyall, Ziyaad Surtee, Zobia Hussain, Zobia Wadi, Zoe Antonia McCarthy, Zoe Austin, Zoë Elspeth Wands, Zoë Halford, Zoe Hinchcliffe, Zoe King, Zoe Louise Moles, Zoe Matthews, Zoe Spink, Zoe Thursz, Zoe Zagorac, Zofia Maja Witkowska, Zofia Zakrzewska, Zohaib Sajid, Zohra Noori, Zunira Areeba Bhuiyan, Zurafa Sakel, Zuzanna Loboda, Zuzanna Skolik.
